# Copper assisted sequence-specific chemical protein conjugation at a single backbone amide

**DOI:** 10.1038/s41467-023-43753-7

**Published:** 2023-12-05

**Authors:** Mengzhun Guo, Kai Zhao, Liang Guo, Rui Zhou, Qiuju He, Kuan Lu, Tian Li, Dandan Liu, Jinfeng Chen, Jing Tang, Xin Fu, Jinyun Zhou, Bei Zheng, Samuel I. Mann, Yongdeng Zhang, Jing Huang, Bing Yang, Ting Zhou, Yingjie Lei, Bobo Dang

**Affiliations:** 1 Fudan University, Shanghai, China; 2 Westlake Laboratory of Life Sciences and Biomedicine, Hangzhou, Zhejiang China; 3 Research Center for Industries of the Future and Key Laboratory of Structural Biology of Zhejiang Province, School of Life Sciences, Westlake University, Hangzhou, Zhejiang China; 4 Institute of Biology, Westlake Institute for Advanced Study, Hangzhou, Zhejiang China; 5 Department of Nuclear Medicine and Positron Emission Tomography Center, The Second Affiliated Hospital of Zhejiang University School of Medicine, Hangzhou, China; 6 Zhejiang Provincial Key Laboratory for Cancer Molecular Cell Biology, Life Sciences Institute, Zhejiang University, Hangzhou, Zhejiang China; 7 Department of Pharmaceutical Chemistry and the Cardiovascular Research Institute, University of California at San Francisco, San Francisco, CA 94158 USA

**Keywords:** Peptides, Chemical modification

## Abstract

Direct, site-specific methods of protein functionalization are highly desirable for biotechnology. However, such methods are challenging due to the difficulty of chemically differentiating a single site within a large protein. Herein, we propose “metal binding targeting” strategy and develop a Copper Assisted Sequence-specific conjugation Tag (*CAST*) method to achieve rapid (second order rate 8.1 M^−1^ s^−1^), site-specific protein backbone chemical modification with pinpoint accuracy. We demonstrate the versatility of *CAST* conjugation by preparing various on-demand modified recombinant proteins, including a homogeneous antibody-drug conjugate with high plasma stability and potent efficacy in vitro and in vivo. Thus, *CAST* provides an efficient and quantitative method to site-specifically attach payloads on large, native proteins.

## Abstract (web summary)

Direct, site-specific methods of protein functionalization are of interest, but challenging due to difficulty in chemically differentiating a single site within a large protein. Here, the authors develop a Copper Assisted Sequence-specific conjugation Tag (*CAST*) method to achieve rapid, site-specific protein backbone chemical modification with pinpoint accuracy, and prepare various on-demand modified recombinant proteins using *CAST*.

## Introduction

The continued development of protein modification methods has contributed significantly to the advancement of modern chemical biology, molecular biology, and medicine. Many of these reported methods involve chemoselectivities that enable amino acid-specific modifications of Cys, Lys, Met, etc^1–4^. However, few of these methods can achieve single site specificity because chemically differentiating between one amino acid type at multiple locations within a large protein is particularly difficult, especially at neutral pH, low micromolar protein concentrations in aqueous buffers with high efficiency and selectivity^3,4^.

Site-specific protein modifications that minimally affect protein functions are often more desirable for a diverse range of applications, such as probe attachment, protein-drug conjugation, and biomaterial construction^3–5^. Currently, these single site-specific protein modifications are generally realized by incorporating unnatural amino acids or enzymatic processes^3,4,6^. Direct single site-specific chemical conjugations through natural amino acids are attractive because of their simplicity, convenience, and higher expression yields (relative to methods requiring biosynthetic incorporation of non-natural amino acids)^3,7–10^. However, achieving such single site-selective protein conjugation has been a formidable challenge in the field. To date, only a few cysteine-based methods have been reported because cysteine has distinct reactivity, and many groups have intensively studied cysteine conjugation for a long time^11–13^. Methods beyond cysteine have not been reported.

Transition metals are essential for organic catalysis. The special property of transition metals can potentially enable novel chemistries on proteins. Thus, numerous attempts have been made to apply transition metal-catalyzed reactions to protein functionalization, and some of those methods have been shown to catalyze chemoselective reactions on Cys, Tyr, Trp, and even backbone amide^3,4,8,14,15^. However, with the exception of terminal selectivity^3,16^, transition metal-catalyzed protein modifications lack single-site specificity. It remains unclear whether such single site-specific chemical modifications can be achieved using transition metal-catalyzed reactions. These single site-specific protein modifications would significantly expand the protein functionalization toolbox to enable downstream applications in protein labeling, therapeutic conjugate preparation, and beyond^17–19^.

**Fig. 1:**
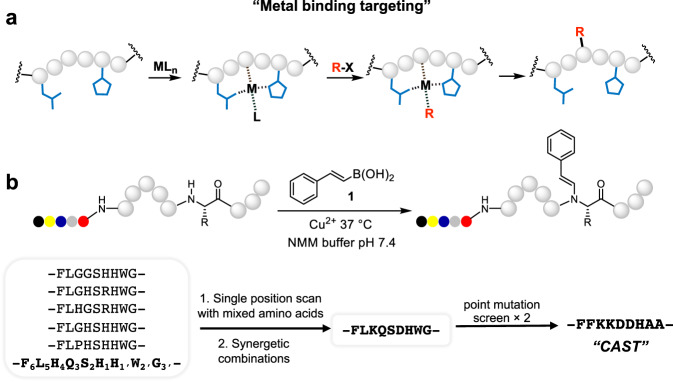
The principle of the “metal binding targeting” strategy and the development of the ***CAST*** conjugation. **a** The principle of using a “metal binding targeting” strategy to develop a unique peptide sequence for single site-specific chemical protein conjugation. **M** is a transition metal ion, **L** is a metal ion ligand, and **R-X** is a reacting small molecule. **b** The development procedure of the ***CAST*** conjugation.

We recently reported that Ni(II) can bind a specific peptide motif (SNAC-tag) to function as a protease for sequence-specific protein cleavage^20^. This SNAC-tag serves as the metal ion ligand to activate the cleavage reaction while simultaneously determining the cleavage site. We hypothesized that site-specific protein conjugations might be achieved through a similar “metal binding targeting” strategy: a peptide motif could specifically bind a metal ion that catalyzes a conjugation reaction with the bound peptide, thus simultaneously achieving chemoselectivity and site specificity (Fig. 1a). We chose a few biocompatible transition metal-catalyzed reactions, including a palladium catalyzed tyrosine-selective alkylation reaction, a dirhodium-catalyzed aromatic side chain modification reaction, and a copper catalyzed backbone amide modification reaction, to test our hypothesis^8,15,21–25^. These reactions were all shown to have chemoselectivity towards one or several types of amino acids, but they lack single site specificity because same amino acid at different sites couldn’t be differentiated. For the backbone amide modification method, N-terminal selective modification can be achieved by introducing an N-terminal pyroglutamic acid (Glp)^15,21^, however, pyroglutamic acid is rarely found in proteins and can only be generated through cascade enzymatic processes, greatly limiting its application scope^21^. In this work, we specifically sought a native amino acid sequence that undergoes the transition metal-catalyzed reaction in a sequence-specific manner to directly achieve single-site modification within large proteins.

## Results

### The development of *CAST* conjugation

We started our investigations by screening the reactions with His-containing peptides that can bind metal ions^20^. The initial results with the Chan-Lam reaction were encouraging, as similar peptide sequences gave different reaction yields (Supplementary Table 1), we then focused our attention on this reaction. The initial rate of the Chan-Lam reaction was quite slow, with the best sequence (-F_6_L_5_H_4_Q_3_S_2_H_1_H_1’_W_2’_G_3’_- at 0.43 mM) leading to ~55% product formation after 12 h (Fig. 1b and Supplementary Fig. 1). Mass spectrometry analysis revealed that the vinyl group from the boronic acid was transferred to the main chain amide nitrogen of residue H_1_ (Fig. 1b and Supplementary Fig. 2a), in which the numbering follows the convention of protease substrates (amino acid, aa, positions are designated aa_1,_ aa_2_, aa_3_ … progressing N-terminally from the reactive site, and aa_1’_, aa_2’_ … progressing toward the C-terminus). We hypothesized that residue H_1’_ served as the anchoring residue that coordinated with the Cu(II) ion via its imidazole side chain (Fig. 1b and Supplementary Fig. 2a)^15^. The H_1_ amide nitrogen should also coordinate Cu(II) since it is the reaction site. This assignment is consistent with the mechanism of the Chan-Lam reaction, in which a deprotonated anionic amide N can first serve as a ligand that then couples to an alkenyl group derived from the boronic acid.

**Fig. 2:**
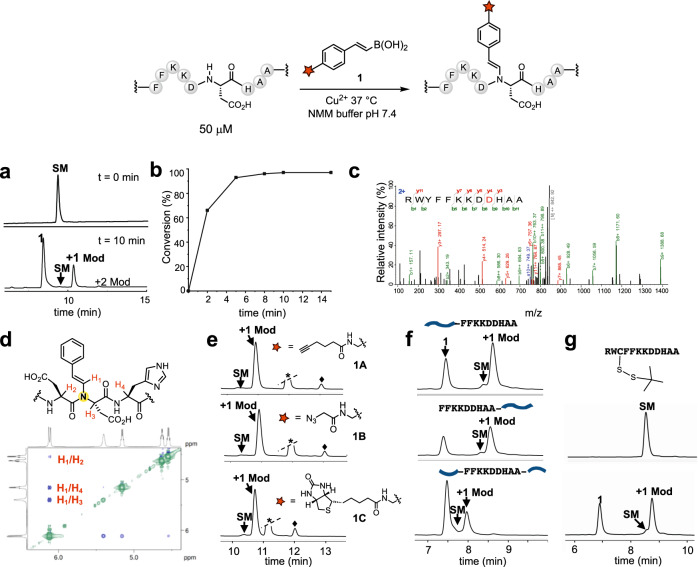
Characterizations of the ***CAST*** conjugation. **a** ***CAST*** peptide reaction with **1**. The chromatograms shown are at 214 nm absorption from LC‒MS analysis. Source data are provided as a Source Data file. **b** ***CAST*** peptide and molecule **1** reaction kinetics study. **c** LC‒MS/MS analysis of the ***CAST*** peptide conjugation product. The spectrum indicates that the modification is at residue D9 colored in red. The observed *b* ions and *y* ions are shown in green and red colors. **d** Partial NOESY spectrum of the ***CAST*** peptide conjugation product. **e** The ***CAST*** peptide maintains reactivity with functional handle-modified styrylboronic acid (**1** **A,** **1B** or **1** **C**). Peaks marked at black star, black diamond are non-peptide byproducts from **1** **A,** **1B**, or **1** **C**. Red star indicates the corresponding modification of the boronic acid. **f** The ***CAST*** peptide maintains reactivity when inserted at the C-terminus, N-terminus, or in the middle of polypeptides. **g** The ***CAST*** conjugation reaction is compatible with the disulfide bond. Note. SM starting material. Mod modified product. Reactions conditions: ***CAST*** peptide (-FFKKDDHAA-, 50 μM), CuCl_2_·2H_2_O (150 μM), trans-beta-styrylboronic acid **1,** **1** **A,** **1B** or **1** **C** (500 μM), NMM (50 mM, pH = 7.4), 37 °C, 10 min (**a**), 15 min (**e**–**g**).

We then randomized each individual residue around the anchoring H_1’_ to identify residues that boosted reaction rates (Fig. 1b and Supplementary Table 2–7). The results showed that the S_2_ position and H_1_ position strongly preferred Asp, Asn, Ser, or His, and other positions showed a range of preferences, which collectively contribute to selectivity (Supplementary Figs. 3–7). We then combined the preferred amino acids at different sites to investigate synergistic effects (Supplementary Table 8–9). One peptide (-FLKQSDHWG-) provided 72% yield within 3 h at 50 μM peptide concentration (Fig. 1b. and Supplementary Fig. 8). Next, we proceeded with two more rounds of sequence optimizations using point mutation scans and preferred residue combinations (Supplementary Table 10–15, Supplementary Figs. 9–11). These efforts resulted in the peptide -FFKKDDHAA-, which possessed the highest reactivity with styrylboronic acid (Figs. 1b and 2a). This peptide will be referred to as copper assisted sequence-specific conjugation tag (***CAST***) hereafter. A second boronic acid conjugation on ***CAST*** could be observed in trace amounts (<2%) during the reaction (Fig. 2a and Supplementary Fig. 11). MS/MS analysis indicated that the reaction site for the 2nd conjugation was on the anchoring His residue (Supplementary Fig. 2b).

A library of boronic acids was then screened against the ***CAST*** peptide; the results again showed that alkenyl and particularly vinylogous boronic acids exhibited greater reactivity than that of arylboronates, and styrylboronic acid exhibited the best reactivity (Supplementary Fig. 12). Upon screening a number of transition metal ions, it was revealed that only Cu(II) efficiently catalyzed the reaction (Supplementary Fig. 13), consistent with the mechanistic and electrochemical requirements of the aerobic Chan-Lam reaction. While 3 equivalents of Cu(II) is sufficient to facilitate the conjugation transformation within 10 min, 1 equivalent of Cu(II) can be used with only a slightly increased reaction time (Supplementary Fig. 14). Not surprisingly, noncoordinating buffers work the best, as N-methylmorpholine (NMM) buffer afforded the highest yield at pH 7.4. Moreover, higher temperatures can be used to boost the reaction rate (up to 37 °C) (Supplementary Figs. 15–17). We generally added excess boronic acid (10 equiv., 500 μM) to drive the reaction rate. Analysis of the reaction kinetics revealed a rate constant of 8.1 M^−1^ s^−1^ and a t_1/2_ of less than 2 min at a ***CAST*** peptide concentration of 50 μM (Fig. 2b, Supplementary Figs. 18–19). MS/MS and 2D NMR experiments confirmed that the reaction site is on the Asp amide nitrogen preceding the coordinating His of the ***CAST*** peptide (Fig. 2c, d)^26^.

The ability to attach functional handles to protein molecules is particularly important; therefore, we synthesized alkyne-, azide-, and biotin-modified styrylboronic acid molecules and demonstrated that these functional handles are all compatible with the conjugation reaction without impeding the reaction rate (Fig. 2e, Supplementary Fig. 20). The ***CAST*** sequence can be inserted at the C-terminus, N-terminus, or in the middle of polypeptides without loss of reactivity (Fig. 2f, Supplementary Fig. 21). The reaction exhibits activity toward unprotected Cys, which was recently reported by the Ball group (Supplementary Fig. 22)^27^. However, a disulfide bond does not undergo conjugation, and therefore, a solvent exposed Cys can be temporarily protected with a disulfide bond while performing the ***CAST*** conjugation if desired (Fig. 2g, Supplementary Fig. 23). To determine the minimum sequence necessary for the reaction, we screened a series of truncated ***CAST*** peptides and found that six amino acids (-FKKDDH-) were sufficient to retain the reaction kinetics (Supplementary Fig. 24). However, to ensure that the reactivity can be retained in different protein systems, we utilized the longer version (-FFKKDDHAA-) for further studies.

### The application of *CAST* for recombinant protein modification

**Fig. 3:**
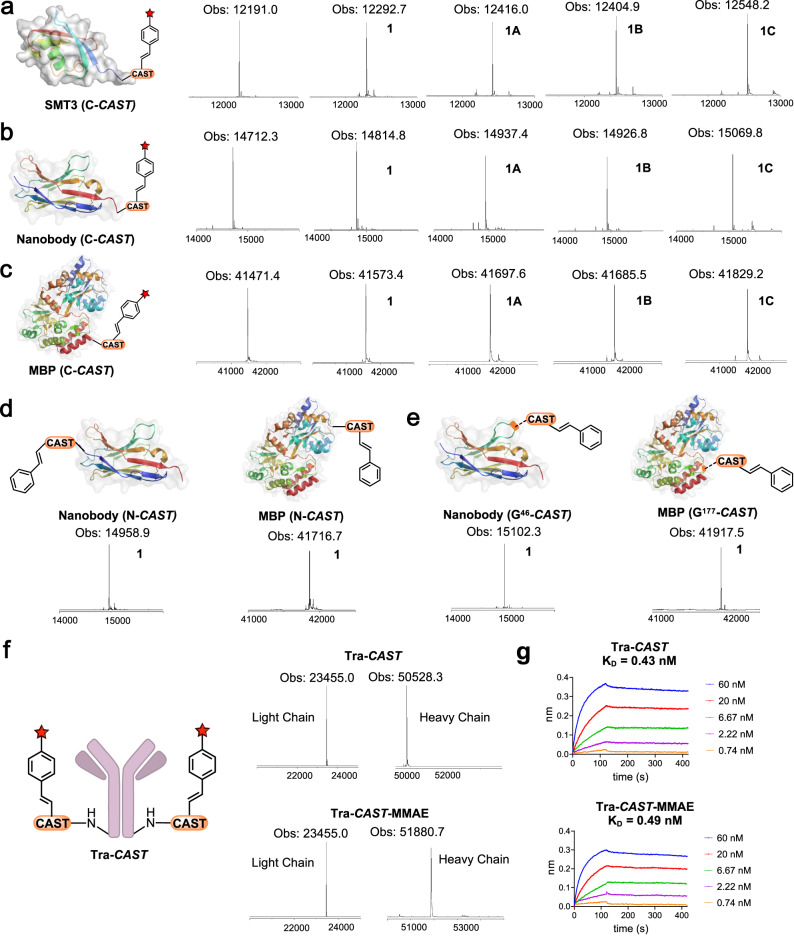
***CAST*** fusion proteins are quantitatively modified with various styrylboronic acid-derived reagents. **a** The characterizations of C-terminal ***CAST*** fused SMT3 reactions with **1,** **1** **A,** **1B** or **1** **C**. **b** The characterizations of C-terminal ***CAST*** fused nanobody reactions with **1,** **1** **A,** **1B** or **1** **C**. **c** The characterizations of C-terminal ***CAST*** fused MBP reaction with **1,** **1** **A,** **1B** or **1** **C**. **d** The characterizations of N-terminal ***CAST*** fused Nanobody or MBP reaction with **1**. **e** The characterizations of internal ***CAST*** inserted Nanobody or MBP reaction with **1**. Conditions for conjugation with **1**: protein (10 μM), CuCl_2_·2H_2_O (50–100 μM), **1** (100 μM) in NMM buffer (50 mM pH 7.4, 0.2 M NaCl), 37 °C, reaction time 10 min – 1.5 h. Conditions for conjugation with **1** **A, 1B, 1** **C:** protein (10 μM), CuCl_2_·2H_2_O (50 μM), **1** **A, 1B** or **1** **C** (500 μM) in NMM buffer (50 mM, pH 7.4, 0.2 M NaCl), 37 °C, reaction time 30 min – 2 h. **f** The characterizations of C-terminal ***CAST*** fused trastuzumab reaction with **SBA-MMAE**, the antibodies were treated with endoglycosidase (EndoS) to remove the N-linked glycans before LC‒MS analysis. Conditions: **Tra-*CAST*** (4 μM), CuCl_2_·2H_2_O (30 μM), **SBA-MMAE** (500 μM), 10% DMF in NMM buffer (50 mM, pH 7.4, 0.2 M NaCl), 37 °C, 7 h. **g Tra-*CAST*** (K_D_ = 0.43 nM) and **Tra-*CAST*-MMAE** (K_D_ = 0.49 nM) binding affinities with HER2 characterizations using biolayer interferometry. Deconvoluted mass spectra of the protein peaks are shown for the unmodified and modified proteins. Red star indicates the corresponding modification of the boronic acid.

With the superior reaction kinetics of the ***CAST*** peptide, we chose five different proteins, including SMT3 (11 kDa), nanobody (15 kDa), maltose-binding protein (MBP, 41 kDa), trigger factor (48 kDa) and sortase (17 kDa), to attach the ***CAST*** peptide at the C-terminus to demonstrate its utility in protein conjugation. For reactions involving proteins, protein concentrations were maintained at 10 μM, 5–10 fold excess copper (50–100 μM) and 10–50 fold excess boronic acid (100–500 μM) were also added when performing reactions on proteins. The protein reaction rates were observed to be slower than those observed in peptides at higher concentrations. Nonetheless, all conjugation reactions with styrylboronic acid led to >95% yield within 2 h, while control proteins lacking the ***CAST*** peptide did not result in observable activity. (Fig. 3a–c, Supplementary Figs. 25–30).

The reactivities for alkyne-, azide-, and biotin-modified styrylboronic acid were very similar for the same protein, and full conversions were achieved within 2 h (Fig. 3a–c, Supplementary Figs. 25–29). For the reactions with sortase, we observed that the single unprotected Cys reacted with styrylboronic acid, agreeing with what we observed on our Cys-containing peptide (Supplementary Fig. 22)^27^. We thus blocked Cys by protecting it with maleimide or 2-mercaptopyridine before performing ***CAST*** conjugation on sortase (Supplementary Figs. 29–31). For 2-mercaptopyridine protection, the temporary protecting group could be removed using TCEP after the conjugation reaction to restore the original Cys (Supplementary Fig. 30). For all reactions between modified styrylboronic acids and proteins, the singly modified adducts comprised over 95% of the product.

The utility of ***CAST*** conjugation can be expanded if ***CAST*** peptide can be inserted at different locations of proteins. Therefore, we first fused ***CAST*** at the N-terminus of nanobody or MBP and demonstrated the conjugation reactions were not affected (Fig. 3d). We then inserted ***CAST*** at the internal loop of nanobody or MBP and showed the conjugation can still proceed efficiently (Fig. 3e). We evaluated the functional consequence of inserting ***CAST*** at different locations of proteins. We performed binding characterizations between different ***CAST*** fused nanobodies and human serum albumin (HSA) and found ***CAST*** insertion didn’t significantly alter the nanobody binding affinity to HSA (Supplementary Fig. 32)^28^. This demonstrated that ***CAST*** could be inserted at nonfunctional regions within a protein to achieve on-demand single-site modification.

### Antibody-drug conjugate (ADC) preparation using *CAST*

Antibody-drug conjugates (ADCs) have emerged as a major class of therapeutics in recent years. To prepare ADCs, a chemical crosslinking reaction is necessary between the antibody and the cytotoxic warhead. To increase the homogeneity and stability of ADCs, site-specific chemical crosslinking methods are now highly desirable to efficiently produce homogeneous and stable ADCs. Currently, cysteine-maleimide conjugation is widely adopted for attaching drug payloads^5,18,19^. However, cysteine-maleimide conjugation products exhibit stability problems in plasma which leads to non-targeted drug release, limiting the therapeutic windows of ADCs^18,29–31^. Moreover, disrupting disulfides can affect antibody stability, and because many disulfides are present in antibodies, site-specific cysteine conjugation may be difficult to achieve, resulting in heterogeneous products^5,19^. The π-clamp and DBCO tag could achieve site specificity, but they still rely on cysteine-based reactions^12,13^. ***CAST*** conjugation is an entirely different reaction system; thus, the step of manipulating antibody disulfides is avoided, and an alternative method is provided in general for the efficient preparation of homogeneous ADCs. Furthermore, we compared the stability of the ***CAST*** conjugation product with the cysteine-maleimide conjugate and found that the ***CAST*** conjugation product is significantly more stable, and no degradation was observed even at elevated temperatures or following the addition of a thiol nucleophile (Supplementary Fig. 33). The excellent conjugation results in the recombinant proteins and the exceptional stability of ***CAST*** conjugation product suggest that the ***CAST*** has the potential for application in ADCs.

We then inserted the ***CAST*** peptide at the C-terminus of the trastuzumab (**Tra**) heavy chain to produce **Tra-*CAST***. There are 3 His residues in each light chain and 11 His residues in each heavy chain in the trastuzumab sequence, site-specifically targeting only the ***CAST*** peptide on the **Tra-*CAST*** while leaving all other sites unaffected indeed seems to be challenging. Remarkably, we found that each heavy chain of **Tra-*CAST*** can react with only one styrylboronic acid molecule while the light chain exhibited no reactivity, the trastuzumab antibody alone showed no observable reactivity with styrylboronic acid (Supplementary Figs. 34, 35). This demonstrated that ***CAST*** can indeed be employed for single site-specific conjugation on antibodies. **Tra-*CAST*** could also react with modified styrylboronic acids **1** **A, 1B** and **1** **C** with essentially the same efficiency as that of styrylboronic acid (Supplementary Figs. 36, 37). To prepare the ADC, we synthesized the styrylboronic acid-Val-Cit-PAB-monomethyl auristatin E (**SBA-MMAE**) conjugate and then performed a single-step conjugation with **Tra-*CAST*** to produce **Tra-*CAST*-MMAE** (Fig. 3f, Supplementary Fig. 37). **Tra-*CAST*-MMAE** was reduced with tris(2-carboxyethyl)phosphine (TCEP) to separate the light chain and heavy chain and further deglycosylated to analyze the conjugation reaction in detail. From the analysis, we again found that the reaction was not observed on the light chain, while the expected single modification was observed on the heavy chain (Supplementary Fig. 37). The binding affinity of **Tra-*CAST*-MMAE** to HER2 (K_D_ 0.49 nM) is the same as that of trastuzumab to HER2 (K_D_ 0.46 nM) (Fig. 3g). For in vitro cellular toxicity, **Tra-*CAST*-MMAE** exhibited no toxicity toward HER2-negative MCF-7 cells but effectively led to cell death of HER2-positive SK-BR-3 cells with EC_50_ values of 0.2 nM (Supplementary Fig. 38)^32,33^. **Tra-*CAST*** showed minimal toxicity in all cell lines at the tested concentrations, **SBA-MMAE** displayed toxicity and could not effectively distinguish HER2-negative MCF7 cells or HER2-positive SK-BR-3 cells (Supplementary Fig. 38).

### In vitro and in vivo efficacy of ADCs prepared using *CAST*

**Fig. 4:**
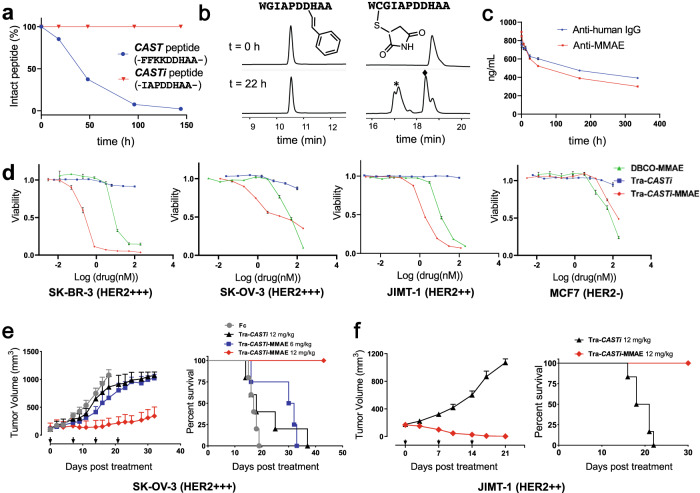
Characterizations of ***CASTi*** and antitumor activity evaluation of **Tra-*CASTi*-MMAE** in vitro and in vivo. **a** ***CAST*** peptide (-FFKKDDHAA-) and ***CASTi*** peptide (-IAPDDHAA-) in vitro stability in mouse plasma. **b 1** modified ***CASTi*** stability comparison with cysteine-maleimide conjugate, incubation conditions: 37 °C, 20 mM glutathione, PBS (pH 7.4), 22 h. Peaks at * are the maleimide hydrolysis byproducts, and the peak at ♦ corresponds to the released free peptide WCGIAPDDHAA. Source data are provided as a Source Data file. **c Tra-*CASTi*-MMAE** stability test in mouse plasma using anti-human IgG or anti-MMAE. *n* = 3 independent experiments, error bars represent s.e.m. **d Tra-*CASTi-*MMAE** (red diamond) effectively killed HER2-positive SK-BR-3 (HER2 + + + ), SK-OV-3 (HER2 + ++) and JIMT-1 (HER2 + +) cells but only showed minimal toxicity against HER2-negative MCF7 (HER2-) cells, **Tra-*CASTi*** (blue square) displayed no obvious toxicity towards all cells, **DBCO-MMAE** (green triangle) showed minimal toxicity towards all cells. *n* = 3 independent experiments, error bars represent s.e.m. **e** In vivo efficacy study on SK-OV-3 xenograft tumor models. Left: tumor volume measurement; right: percent survival (female Balb/c nude mice, *n* = 5 for 12 mg/kg Fc isotype control, *n* = 5 for 12 mg/kg **Tra-*CASTi***, *n* = 4 for 6 mg/kg **Tra-*CASTi-*MMAE**, *n* = 6 for 12 mg/kg **Tra-*CASTi-*MMAE**), black arrow indicates i.v. drug administration, error bars represent s.e.m. **f** In vivo efficacy study on JIMT-1 xenograft tumor models. Left: tumor volume measurement; right: percent survival (female NSG mice, *n* = 6 for **Tra-*CASTi*-MMAE**, *n* = 6 for **Tra-*CASTi***), black arrow indicates i.p. drug administration, error bars represent s.e.m.

To evaluate whether the ***CAST*** peptide was compatible with in vivo applications, we assessed the in vivo efficacy of ***CAST*** peptide-based ADC against xenograft tumor models in mice. Initial assessment revealed that the plasma stability of ***CAST*** peptide (-FFKKDDHAA-) was not optimal (Fig. 4a, Supplementary Fig. 39). To improve the plasma stability, we used our sequence optimization data and generated an additional 15 similar peptide sequences with reaction kinetics comparable to those of ***CAST*** (Supplementary Table 16). The plasma and serum stability were evaluated, and we discovered that the -IAPDDHAA- (***CASTi***) sequence had superior stability in plasma and serum and remained intact following incubation in plasma for 144 h (Fig. 4a, Supplementary Fig. 39 and Supplementary Table 17). ***CASTi***-conjugate also exhibits exceptional stability in the presence of excess thiol-nucleophile (glutathione) while cysteine-maleimide conjugate degraded significantly under the same conditions which again highlights the stability advantage of ***CASTi*** conjugate (Fig. 4b). We then inserted -IAPDDHAA- (***CASTi***) into the C-terminus of the Fc domain to provide **Tra-*CASTi***. To produce the ADC in large quantities, we reacted **Tra-*CASTi*** with **1B** (Supplementary Figs. 40–42), followed by DBCO-PEG_3_-Glu-Val-Cit-PAB-MMAE **(DBCO-MMAE)** to produce **Tra-*CASTi*-MMAE** for further in vitro and in vivo studies (Supplementary Fig. 40). We used the -Glu-Val-Cit- linker to construct **Tra-*CASTi*-MMAE** because the -Val-Cit- linker was reported to exhibit poor stability in the mouse plasma^34^. **Tra-*CASTi*-MMAE** indeed exhibited excellent stability in mouse plasma (Fig. 4c). We confirmed that **Tra-*CASTi*-MMAE** can effectively kill HER2-positive SK-BR-3 cells (HER2 + + + , EC_50_ = 0.2 + /−0.02 nM), SK-OV-3 cells (HER2 + + + , EC_50_ = 1.3 + /−0.5 nM) and JIMT-1 cells (HER2 + + , EC_50_ = 1.7 + /−0.1 nM), while it exhibits minimal toxicity to HER2-negative MCF-7 cells (Fig. 4d)^32,33^. We also quantified the residue copper ion in **Tra-*CASTi*-MMAE** using ICP-MS, we found that the copper concentration detected was ~0.29 μg/L (4.6 nM) when **Tra-*CASTi*-MMAE** (0.208 mg/mL) was submitted for analysis (Supplementary Fig. 43, Supplementary Table 18–19). Since free human serum copper concentration is in the low μM range, and total human serum copper concentration is around 10–20 μM^35,36^. The residue copper left in **Tra-*CASTi*-MMAE** will not cause toxicity for in vivo studies.

We continually evaluated the in vivo antitumor efficacy of **Tra-*CASTi*-MMAE** in HER2-positive human ovarian cancer SK-OV-3 (HER2 + ++) and human breast ductal adenocarcinoma JIMT-1 (HER2 + +) xenograft models^32,33^. For the SK-OV-3 xenograft models, **Tra-*CASTi*-MMAE** (12 mg/kg or 6 mg/kg), **Tra-*CASTi*** (12 mg/kg), and the Fc isotype control (12 mg/kg) were intravenously (i.v.) administered to tumor-bearing mice weekly for four doses. Tumors in mice receiving **Tra-*CASTi*-MMAE** were greatly suppressed, as evidenced by a strong dose-dependent tumor inhibition and survival rate (Fig. 4e). Tumors in the Fc or **Tra-*CASTi*** control group mice rapidly reached 1000 mm^3^, which is the humane end point of the study (Fig. 4e). For the JIMT-1 xenograft models, three doses of intraperitoneally (i.p.) administered **Tra-*CASTi*-MMAE** (12 mg/kg) induced significant tumor regression, while no obvious tumor suppression was observed in mice receiving **Tra-*CASTi*** (12 mg/kg) (Fig. 4f). No obvious toxicity was observed in any group throughout the course of the study, as determined through monitoring the body weight (Supplementary Fig. 44). These results demonstrate that ***CASTi*** is indeed compatible with in vivo therapeutic conjugate applications.

## Discussion

There have been increasing demands for selective and efficient methods of chemical protein modification in chemical biology, bioengineering, and medicine in recent years. Although a handful of chemoselective protein modification methods have been reported, only a limited number of these methods can proceed at neutral pH with high conversion at low micromolar concentrations, while few of them also possess single-site specificity. Direct site-specific chemical protein conjugations through canonical amino acids are highly attractive because they minimally affect protein functions after modification. However, such transformations are difficult to achieve because the same amino acid type exhibits a similar chemical environment at different protein sites, thus making them difficult to be differentiated.

Transition metal-catalyzed reactions have been widely adopted in protein modifications in recent years. However, such reactions lack single-site specificity in general. Herein, we proposed the “metal binding targeting” strategy as the guiding principle to develop novel site-specific modification methods of native proteins through sequence-specificity. Following this principle, we successfully developed ***CAST*** to achieve single site-specific modification on the presumed inert backbone amides for applications in protein conjugation. We demonstrated that ***CAST*** peptide can be inserted at different locations of proteins, including N-terminus, internal loop, and C-terminus, to modify proteins efficiently and quantitatively. We were also able to prepare a homogeneous and stable antibody drug conjugate (**Tra-*CASTi*-MMAE**) using plasma stable ***CASTi*** and showed that **Tra-*CASTi*-MMAE** exhibits potent efficacy in vitro and in vivo. Furthermore, ***CAST*** conjugation is orthogonal to all currently reported chemical or enzymatic conjugation methods, which enables ***CAST*** to be used in combination with other methods when desired. For example, dual-payload antibody drug conjugates are currently desired to overcome tumor resistance^37,38^, ***CAST*** conjugation could be used in conjunction with other methods, including Cys-based methods, to readily prepare such dual-payload antibody drug conjugates.

As a potential go-to option for protein labeling, we compared ***CAST*** conjugation with the most widely used sortase ligation utilizing the fully evolved sortase by Liu and colleagues^39^. We found that ***CAST*** conjugation is indeed more efficient than sortase ligation under similar conditions for protein labeling (Supplementary Figs. 45, 46). In addition, we compared ***CAST*** conjugation with the reported π-clamp conjugation since π-clamp is also a Cys-based sequence-specific chemical conjugation method (Supplementary Figs. 47–49)^12^. In our hands, we found π-clamp conjugation reaction rates on peptide, sortase or trastuzumab are all similar to the previous report, with a peptide reaction rate of 0.73 M^−1^ s^−1^, which is significantly slower than ***CAST*** conjugation rate (8.1 M^−1^ s^−1^). We further compared ***CAST*** with thiobridge technology even though thiobridge is only a chemoselective reaction and cannot distinguish Cys at different sites. Thiobridge could produce stable ADC, and one ADC prepared using thiobridge (OBI-999) has entered phase II clinical trial^5^. We found thiobridge reaction generally required more than 16 h to complete (Supplementary Fig. 50). Furthermore, we noticed the presence of incomplete conjugated antibodies in the final conjugation product, even with 100-fold excess small molecule added (Supplementary Fig. 50). This phenomenon is commonly observed when employing the disulfide rebridging strategy^5^. In comparison, ***CAST*** conjugation can achieve near quantitative reaction conversion within 4 h to produce homogeneous products (Supplementary Fig. 40, Supplementary Fig. 50).

Although we showed that ***CAST*** conjugation can achieve quantitative labeling in general, there could still be background labeling on proteins. Thus, we assessed these background labeling on SMT3 (C-***CAST***), MBP (C-***CAST***) and nanobody (C-***CAST***) using proteomics analysis. We indeed found labeling on peptides beyond ***CAST***, this is not surprising because proteomics analysis is highly sensitive, and even <1% modification can be readily identified (Supplementary Table 18). We synthesized these peptides identified by proteomic analysis and performed the labeling reaction under similar conditions used for their parent proteins labeling, only 1 out of the 21 peptides had ~1% modification (Supplementary Table 20), and all the other 20 peptides had no observable modifications. Thus, the fast reaction kinetics of ***CAST*** ensured the success of selective labeling. To further evaluate the specificity of ***CAST*** conjugation, we assessed ***CAST***-mediated single protein labeling in BL21 bacteria cell lysate or 293 F mammalian cell lysate (Supplementary Fig. 51). We carried out MBP(C-***CAST***) and Tra-***CASTi*** conjugation with **1** **C** in the cell lysate and then used western blot analysis to evaluate the labeling specificity. We were able to demonstrate that the conjugation of MBP(C-***CAST***) and Tra-***CASTi*** with **1** **C** could indeed proceed cleanly according to western blot analysis, which indicated that single protein labeling using ***CAST*** conjugation in cell lysate is feasible, highlighting its potential applications beyond purified protein modifications (Supplementary Fig. 51).

Through this study, we demonstrated that direct site-specific chemical protein modification can indeed be achieved using transition metal-catalyzed reactions in a sequence-specific manner on natural amino acids. We believe this work will encourage more chemists to generate new ideas and explore a diverse range of reactions for novel chemical protein modifications and their further applications in chemical biology, biotechnology, and medicine.

## Methods

### Peptide synthesis

All peptides were synthesized on a 0.01 mmol scale using automated parallel peptide synthesizer (Syro II, Biotage). All reactions were carried out at room temperature unless otherwise stated. Each amino acid synthesis cycle includes 12 min coupling with 200 µL Fmoc-protected amino acid (0.53 M in DMF), 200 µL HCTU (0.5 M in DMF) and 100 µL *N, N*-diisopropylethylamine (2 M in NMP) twice, 3 min wash with DMF three times, deprotection with 20% (v/v) 4-methyl piperidine in DMF once and 3 min wash with DMF three times. After completion of the stepwise SPPS, the resins were washed thoroughly with DCM and dried under vacuum. The peptides were then cleaved off the resins and side-chains were deprotected by treatment with 2% (v/v) water, 2% (v/v) triisoproprylsilane and 1% (m/v) DTT in neat trifluoroacetic acid (TFA) for 2 h at room temperature. The resulting solution containing peptide was precipitated and washed with cold diethyl ether three times. The obtained solid was dissolved in 50% H_2_O: 50% acetonitrile containing 0.1% TFA and lyophilized.

### General procedure for peptide modification

To a solution of peptide RWYFFKKDDHAA (1 µL of 5 mM stock solution in water, 0.05 mM final concentration) in NMM buffer (97 µL of 50 mM stock solution, pH 7.4), boronic acid (1 µL of 50 mM stock solution in DMSO, 0.5 mM final concentration) and CuCl_2_·2H_2_O (1 µL of 15 mM stock solution in water, 0.15 mM final concentration) were subsequently added. The mixture was vortexed and shaken for 15 min at 37 °C. Na_2_-EDTA (2 µL of 500 mM stock solution in H_2_O, 20 mM final concentration) was added to quench the reactions. Then, the crude reaction mixture was centrifuged to remove any precipitates, and the supernatant was analyzed by LC‒MS to determine the reaction yield.

### General procedure for the preparation of Tra-*CAST*-MMAE

To a solution of **Tra-*CAST*** (4 μM final concentration) in NMM buffer (50 mM, 0.2 M NaCl, pH 7.4), **SBA-MMAE** (1 µL of 25 mM stock solution in DMF, 0.5 mM final concentration) and CuCl_2_·2H_2_O (1.5 µL of 1.0 mM stock solution in water, 30 μM final concentration), 5 µL DMF were added subsequently. The total reaction volume is 50 µL. The mixture was incubated at 37 °C for 7 h. After reaction is completed, Na_2_-EDTA (2 µL of 500 mM stock solution in H_2_O, 20 mM final concentration) was added the crude reaction mixture was directly injected onto LC-MS for analysis.

### General procedure for the preparation of Tra-*CASTi*-MMAE

To a solution of **Tra-*CASTi*** (4 μM final concentration) in NMM buffer (50 mM, 0.2 M NaCl, pH 7.4), boronic acid reagent **1B** (20 µL of 12.5 mM stock solution in DMF, 0.25 mM final concentration) and CuCl_2_·2H_2_O (20 µL of 1.0 mM stock solution in water, 20 μM final concentration) were subsequently added. The total reaction volume is 1 mL. The mixture was incubated at 37 °C for 4 h. After the reaction was completed, Na_2_-EDTA (40 µL of 500 mM stock solution in H_2_O, 20 mM final concentration) was added. Excessive **1B** is removed during dialysis. **Click reactions for MMAE installation: DBCO-MMAE** (**h**) (4 µL of 20 mM stock solution in DMSO, 20 equivalents) was added to a solution of the **1B** modified **Tra-*CASTi*** (4 μM, total reaction volume is 1 mL) conjugate in PBS, and the mixture was incubated at 37 °C for 2 h. The reaction was monitored using LC-MS. After the reaction was completed, excess **DBCO-MMAE** was removed during dialysis.

### BioLayer interferometry binding assay

In vitro binding assays were performed using Fortebio Octet BioLayer Interferometry system at room temperature^40^. Briefly, AHC tips were dipped into 200 μL of antibody solution (10 μg/mL **Tra**-***CAST***or **Tra-*CAST-*MMAE** in PBS with 0.1% BSA and 0.02% tween20) for the loading of antibodies. The tips loaded with antibody were sampled with recombinant HER2 (Sino biological 10004-H08H1-50) at various HER2 concentrations in PBS with 0.1% BSA and 0.02% tween 20 to obtain the association curve, buffer only serves as the reference. After association, the tips were dipped into PBS with 0.1% BSA and 0.02% tween 20 to obtain the dissociation curve. Following the protocols provided by Fortebio Biosystems, the association and dissociation curves of each sample were manually fitted using ForteBio DataAnalysis 12 to obtain the K_D_. The final K_D_ was reported as the average of the K_D_ obtained from experiments with serially diluted HER2.Our selected nanobody binds to HSA^28^, The functional impact of inserting ***CAST*** to nanobody was evaluated by measuring the affinity to HSA using Biolayer Interferometry at room temperature. The anti-His tips dipped into 200 μl of human HSA solution (10 μg/ml HSA with his-tag in PBS with 0.02% tween 20) for the loading of HSA. The tips loaded with HSA were sampled with ***CAST***-nanobodies at various concentrations in PBS with 0.02% tween 20 to obtain the association curve. The buffer served as the reference. After association, the tips were dipped into PBS with 0.02% ween 20 to obtain the dissociation curve. After the emperiments, the association and dissociation curves of each sample were manually fitted to obtain the K_D_. The final K_D_ was reported as the average of the K_D_ obtained from experiments with serial diluted nanobodies.

### Cell assays

Cells were seeded in a 96-well white opaque plate at a density of 5 × 10^3^/well (CHO, ATCC, CCL-61) or 1 × 10^4^/well (MCF7 (ATCC, HTB-22), SK-BR-3 (ATCC, HTB-30), SK-OV-3 (ATCC, HTB-77) or JIMT-1(Beyotime, C6453)). Cells were allowed to attach for 24 h at 37 °C and 5% CO_2_ in humidified atmosphere. Cells were then treated with serial dilutions of **Tra-*CAST***, **Tra-*CAST-*MMAE** and **SBA-MMAE** or **Tra-*CASTi***, **Tra-*CASTi-*MMAE** and **DBCO-MMAE** for 96 h (BT474, MCF7, SK-BR-3). Cell viability was determined using CellTiter Glo reagents (G7571) and was normalized to the control cells. Data was analyzed by Graphpad software, and the half-maximal effective concentration (EC_50_) value was calculated by fitting with the log(inhibitor) vs. response module.

### Tra-*CASTi*-MMAE plasma stability determination

**Tra-*CASTi*-MMAE** (100 µg/mL, 1.2 µL in PBS) was added to BALB/c mouse plasma (98.8 µL). The sample was then incubated at 37 °C and aliquoted at different time points to store at −80 °C for later use. The **Tra-*CASTi*-MMAE** stability was then assessed using a sandwich ELISA assay. In the sandwich ELISA assay, homemade HER2 protein (100 ng per well) was used to coat the high-binding 96-well plate (Corning) overnight at 4 °C. 200 µL PBST (2% BSA, 0.05% Tween 20) was then used to do the blocking at room temperature for 2 h. The aliquoted ADC samples (100 µL in PBS-T containing 2% BSA) were then added to incubate at 4 °C overnight. PBS-T buffer was then used to wash the plate four times. 100 µL of rabbit anti-human IgG antibody (1:5000) was used to detect trastuzumab (room temperature, 2 h incubation). After washing it four times. 100 μL secondary goat anti-rabbit (1:5000) was added and then washed four times with 200 μL PBS-T before adding 100 μL the TMB substrate. After color was developed for 10–30 mins, 100 μL of 2 M HCl was added to each well to stop the reaction. Then the absorbance at 450 nm was recorded using a plate reader (Thermo Varioskan LUX). Concentrations were calculated based on a standard curve. MMAE conjugate stability assays were performed in the same manner using homemade human HER2 (100 ng per well) for plate coating, mouse anti-MMAE antibody (1:5000), and goat anti-mouse IgG–HRP conjugate (1:5000) as secondary detection antibodies, respectively. Antibodies information: Mouse anti-MMAE (primary antibody; from ACRO; Cat. MME-M5252; clone name: N/A from a hybridoma resulting from fusion of SP2/0 myeloma and B-lymphocytes obtained from a mouse immunized with MMAE; Lot S104-218QF1-Y9.) Rabbit anti-human IgG primary antibody; from Abcam; Cat. ab181236; clone name: EPR12700; Lot GR3345859-5. Goat anti-Mouse IgG (secondary antibody; from Beyotime; Cat. A0216; clone name: N/A; Lot N/A).

### Peptide in vitro serum/plasma stability assay

Fresh blood was obtained from Male BALB/c mice (8 weeks old) from the Laboratory Animal Resources Center of Westlake University. The serum was prepared by centrifugation at 1500 g for 10 min after standing at room temperature for 30 min. The plasma was prepared by centrifugation at 1500 g for 20 min. Peptide was individually incubated with fresh serum at 2 mM at 37 °C respectively. Samples were taken at 0 h, 3 h, and 21 h. Then acetonitrile at 75% final concentration was added to serum samples to precipitate plasma proteins, precipitates were removed by centrifugation at 12,000 g for 5 min. The supernatant was diluted 20 times with 0.1%TFA/H_2_O (v/v) and analyzed by LC/MS.

### SDS-PAGE and western blot analysis of single protein labeling in cell lysates

After quenching the copper reaction by the addition of EDTA, the reaction mixture was mixed with 4×LDS loading buffer (Life Technologies NP0008). Protein denaturation was performed at 98 °C for 5 min. After cooling to r.t., the sample was loaded to a 4%–20% bis-tris gel (Genscript). Electrophoretic separation was conducted at 180 V for 40 min. For protein staining, Coomassie soln was applied to the gel for 1 h and subsequently destained with water.

For Western blot analysis, the protein gel was transferred to a blot membrane using Bio-rad Tran-Blot SD Semi-Dry Electrophoretic Transfer Cell at 25 V for 25 min. The blot membrane was subjected to a blocking process with 5% w/w aq skim milk powder in TBST buffer (150 mM NaCl, 25 mM Tris, 0.1% Tween-20, pH 7.4) at 4 °C overnight. Anti-Biotin antibody [Hyb-8] (20 µL, ab201341) was added to the membrane in 4 mL of the blocking solution, and the membrane in the solutionwas shaken at r.t. for 1 h. After discarding the liquid, the membrane was washed with TBS buffer (150 mM NaCl, 25 mM Tris, pH 7.4) three times. HRP-labeled Goat Anti-Mouse IgG(H + L) (20 µL, Beyotime, A0216) was added to the membrane in 4 mL of the blocking solution, and the membrane in the solutionwas shaken at r.t. for 1 h. The membrane was washed with TBS buffer (150 mM NaCl, 25 mM Tris, pH 7.4) three times. Membrane imaging was performed on Amersham Imager 680 using 2 mL Super ECL Detection Reagent (Yesen Biotechnology). Antibodies information: Rabbit anti-biotin (primary antibody; from Abcam; Cat. ab201341; clone name: Hyb-8; Lot N/A). Goat anti-Rabbit IgG (secondary antibody; from Beyotime; Cat. A0208; clone name:N/A; Lot N/A).

### Animal model preparation and in vivo antitumor efficacy validation experiments

All procedures were approved by the Institutional Animal Care and Use Committee of Zhejiang University or Westlake University. Mice are required to be sacrificed when the xenograft tumor size reaches 1000 mm^3^. Female Balb/c nude mice (age, 4 weeks) purchased from Ziyuan Laboratory Animal lnc. (Hangzhou, China). Mice were acclimated for 1 week before the experiment and kept under standard laboratory conditions with food and water provided ad libitum. HER2-positive human ovarian xenograft tumor model was used to evaluate the antitumor effect of ADC compounds (**Tra-*CASTi*-MMAE**). Briefly, cultured SK-OV-3 cells were suspended in DMEM medium without serum and antibiotics. Mice received 100 μL subcutaneous injection of SK-OV-3 cell suspension (1 × 10^7^/100 μL). Tumor volume was calculated by using the following formula: Tumor volume = 0.52 × Length × Width^2^. When the volume of xenograft tumor reached average of 100–150 mm^3^, the mice were randomly divided into four groups. The **Tra-*CASTi*-MMAE** protein (12 or 6 mg/kg) and control (**Tra-*CASTi***, 12 mg/kg; Fc, 12 mg/kg) were administered to mice via the tail vein on days 0, 7, 14 and 28. Tumor volume and body weight were monitored three times a week. When xenograft tumors grew to 1000 mm^3^, the mice were killed. Female NSG mice (age, 4 weeks) were purchased from Shanghai Jihui (Shjh) Laboratory Animals Care Co. Ltd. Briefly, cultured JIMT-1 cells were suspended in DMEM medium without serum and antibiotics. NSG mice received 100 μL subcutaneous injection of JIMT-1 cell suspension (1 × 10^7^/100 μL). Tumor volume was calculated by using the following formula: Tumor volume = 0.52 × Length × Width^2^. When the volume of xenograft tumor reached average of 100–150 mm^3^, the mice were randomly divided into two groups. The **Tra-*CASTi*-MMAE** protein (12 mg/kg) and control (**Tra-*CASTi***, 12 mg/kg) were intraperitoneally administered to mice on days 0, 7 and 14. Tumor volume and body weight were monitored twice a week. When xenograft tumors grew to 1000 mm^3^, the mice were killed.

### Reporting summary

Further information on research design is available in the Nature Portfolio Reporting Summary linked to this article.

### Supplementary information

Supplementary information

Reporting Summary

### Source data

Source Data

## Data Availability

The authors declare that all the data supporting the findings of this study are available within the paper, its supplementary information file, or from the corresponding author upon request. Source data are provided with this paper.
