# Supplementary material for: Copper assisted sequence-specific chemical protein conjugation at a single backbone amide
Source: Nat Commun. 2023 Dec 5;14:8063. doi: 10.1038/s41467-023-43753-7 (PMC10698186; doi:10.1038/s41467-023-43753-7)
Supplement: Supplementary file 1 — Supplementary information [file 41467_2023_43753_MOESM1_ESM.pdf]

Supplementary Information

Table of Contents

Supplementary Materials.. 1

1. General information 2

   a. Chemicals.. 2

   b. Peptide synthesis.. 2

   c. HPLC and LC-MS analyses.. 3

2. Sequence optimization 3

3. Experimental procedures and reaction optimizations 14

4. Preparation of boronic acid derivatives.. 27

5. Preparation of Proteins 33

   a. Expression and purification of recombinant proteins:.. 33

   b. Expression and purification of antibodies 38

6.Reactions on proteins 40

7.Protein raw data 46

8.Exploration of similar ***CAST*** sequence with high stability in vitro 57

   a.Reaction efficiency of similar ***CAST*** sequences... 57

   b.Plasma/Serum stability determination.. 58

9. Determination of copper concentration in ***Tra-CASTi-MMAE*** 62

10.BioLayer interferometry binding assay.. 64

11. Cell assays 64

12.Animal model preparation and in vivo antitumor experiment 64

13. Supplementary figures and tables 66

14.Protein reaction in cell lysate 69

15.NMR spectra 71

16. Supplementary References.. 81

1. General information

a. Chemicals

All chemicals were purchased from commercial vendors and used without further purifications. 1-[Bis(dimethylamino)methylen]-5-chlorobenzotriazolium 3-oxide hexafluorophosphate (HCTU), Fmoc-L-Gly-OH, Fmoc-L-Leu-OH, Fmoc-L-Ile-OH, Fmoc-L-Val-OH, Fmoc-L-Lys(Boc)-OH, Fmoc-L-Ala-OH, Fmoc-L-Cys(Trt)-OH, Fmoc-L-Gln(Trt)-OH, Fmoc-L-Asn(Trt)-OH, Fmoc-L-Glu(O^{t}Bu)-OH, Fmoc-L-Asp(O^{t}Bu)-OH, Fmoc-L-Arg(Pbf)-OH, Fmoc-L-Phe-OH, Fmoc-L-Trp(Boc)-OH, Fmoc-His(Boc)-OH, Fmoc-L-Ser(^{t}Bu)-OH, Fmoc-L-Thr(^{t}Bu)-OH, Fmoc-L-Tyr(^{t}Bu)-OH, Fmoc-L-Pro-OH, Fmoc-L-Met-OH were purchased from GL Biochem. Fmoc-Rink amide resin were purchased from tjhecheng. *N, N*-dimethylformamide (DMF), N-Methylpyrrolidone (NMP), dichloromethane (DCM), diethyl ether, Trifluoroacetic acid, 4-methyl pepiridine and HPLC-grade acetonitrile were obtained from Energy Chemical.

Buffers

NMM, *N*-Methylmorpholine (50 mM, 0.2 M NaCl, pH 7.4)
CHES, *N*-Cyclohexyltaurine (0.2 M, 0.2 M NaCl, pH 7.4)
Tris, Tris(hydroxymethyl)aminoethane (50 mM, 0.2 M NaCl, pH 7.4)
HEPES, 4-(2-hydroxyethyl)-1-piperazineethanesulfonic acid (0.1 M, 0.2 M NaCl, pH 7.4)
PBS, Phosphate buffered saline (10 mM, 0.2 M NaCl, pH 7.4)

NMR

NMR spectra were recorded on Bruker AVANCE 500 or AVANCE 600 spectrometer in $CDCl_3$ or DMSO-d_6 using tetramethylsilane (TMS) as internal standard unless otherwise stated. Data for ^{1}H NMR are recorded as follows: chemical shift (δ, ppm), multiplicity (s = singlet, d = doublet, t = triplet, m = multiplet, q = quartet, dd = doublet of doublets, dt = doublet of triplets, td = triplet of doublets, and br = broad signal, coupling constant (s) in Hz, and integration. Data for ^{13}C NMR are reported in terms of chemical shift (δ, ppm)

b. Peptide synthesis

All peptides were synthesized on a 0.01 mmol scale using automated parallel peptide synthesizer (Syro II, Biotage). All reactions were carried out at room temperature unless otherwise stated. Each amino acid synthesis cycle includes 12 minutes coupling with 200 µL Fmoc-protected amino acid (0.53 M in DMF), 200 µL HCTU (0.5 M in DMF) and 100 µL *N, N*-diisopropylethylamine (2 M in NMP) twice, 3 minutes wash with DMF three times, deprotection with 20% (v/v) 4-methyl piperidine in DMF once and 3 minutes wash with DMF three times. After completion of the stepwise SPPS, the resins were washed thoroughly with DCM and dried under vacuum. The peptides were then cleaved off the resins and side-chains were deprotected by treatment with 2% (v/v) water, 2% (v/v) triisoproprylsilane and 1% (m/v)

DTT in neat trifluoroacetic acid (TFA) for 2 hours at room temperature. The resulting solution containing peptide was precipitated and washed with cold diethyl ether three times. The obtained solid was dissolved in 50% H_2O : 50% acetonitrile containing 0.1% TFA and lyophilized.

c. HPLC and LC-MS analyses.

LC-MS analyses were performed using an Agilent 1260-6230 single TOF LC/MS. The column used was Agilent ZORBAX 3.5 μm 300SB-C18 and Agilent ZORBAX 5 μm 300SB-CN.

2. Sequence optimization

Standard reaction conditions for sequence optimizations： To a solution of peptide (8.6 μL of 5 mM stock solution in water, 0.43 mM final concentration) in NMM buffer (82.4 μL of 5 mM stock solution, pH 7.4), boronic acid reagent (2 μL of 50 mM stock solution in DMSO, 1.0 mM final concentration) and $CuCl_2 \cdot 2H_2O$ (7 μL of 5 mM stock solution in water, 0.35 mM final concentration) were added subsequently. The mixture was vortexed and shaken at 37 °C. Ethylenediaminetetraacetic acid tetrasodium salt (Na_4-EDTA, dihydrate) (2 μL of 500 mM stock solution in H_2O, 20 mM final concentration) was added to quench the reactions, then the crude reaction mixture was centrifuged and analyzed by LC-MS to determine the reaction yield.

a. Initial screen of boronic acid reagents[1]

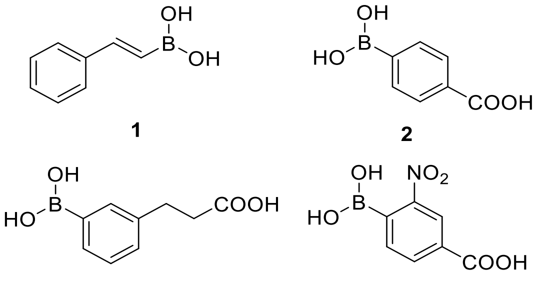

Supplementary Table 1. Reactions with metal recognizing peptides

Sequence	**1**	**2**	**3**	**4**
YFLGGSHHWG	Trace	0%	Trace	0%
YFLGHSRHWG	0%	0%	0%	0%
YFLHGSRHWG	Trace	0%	Trace	0%
YFLGHSHHWG	21%	0%	11%	0%
YFLPHSHHWG	50%	0%	12.5%	0%
YFLHQSHHWG	55%	0%	9%	0%

Conjugation conditions： Following the standard conditions, the mixture was vortexed and shaken for 12 hours at 37 °C.

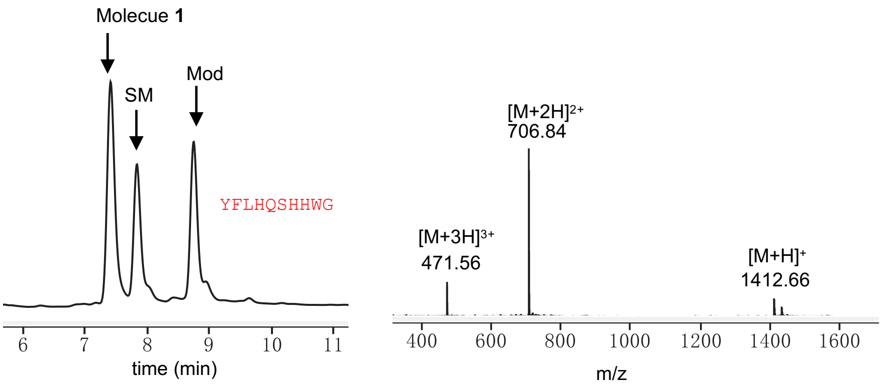

Supplementary Figure 1. Peptide `YFLHQSHHWG` reaction analysis using LC-MS. Starting material abbreviated as SM, modified product abbreviated as Mod.

MS/MS analysis. (Thermo Scientific Q Exactive HE X)

a

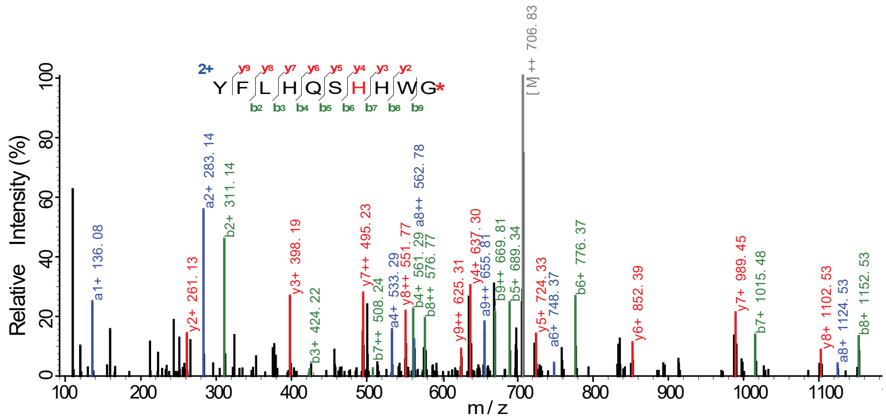

b

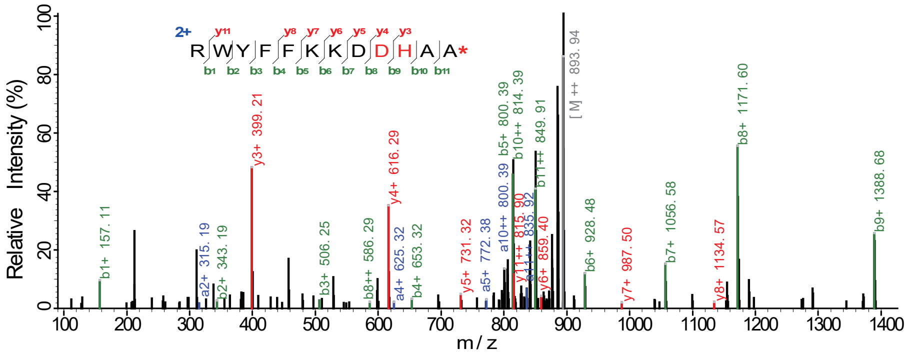

Supplementary Figure 2. Q-Exactive MS/MS spectra of (E)-styrylboronic acid-modified peptides `YFLHQSHHWG` and `RWYFFKKDDHAA`. *b* Ion and *y* ion fragments observed in the MS/MS spectrum are labeled accordingly. (a) Fragmentation ladder for (E)-styrylboronic acid-modified `YFLHQSHHWG`. The modification site is at the His residue in red. (b) Fragmentation ladder for (E)-styrylboronic acid-modified `RWYFFKKDDHAA`. The modifications observed at the Asp and His residues are shown in red.

b. Screening of mixed peptides

Amino acids mixture: A, D, E, H, L, K, M, P, S, N, Q, Y, W, I, R.

These amino acids were mixed in a certain proportion[2] to ensure equal distribution of the synthesized peptides. The amino acid mixture was coupled at different peptide positions to evaluate the amino acid identity influence on the conjugation yield of the corresponding peptide.

Supplementary Table 2. Amino acid ratio in the mixture[2]

Amino acid	Molar ratio
A	0.75
D	1.2
E	1.00
H	0.78
L	0.77
K	1.00
M	0.71
P	0.86
S	0.72
N	1.20
Q	1.00
Y	0.71
W	0.70
I	2.29
R	1.10

1. RWYFLHQ**M_1**HHWG

Supplementary Table 3: Sequences included in the test.

1	RWYFLHQAHHWG	6	RWYFLHQKHHWG	11	RWYFLHQQHHWG
2	RWYFLHQDHHWG	7	RWYFLHQMHHWG	12	RWYFLHQYHHWG
3	RWYFLHQEHHWG	8	RWYFLHQPHHWG	13	RWYFLHQWHHWG
4	RWYFLHQHHHWG	9	RWYFLHQSHHWG	14	RWYFLHQIHHWG
5	RWYFLHQLHHWG	10	RWYFLHQNHHWG	15	RWYFLHQRHHWG

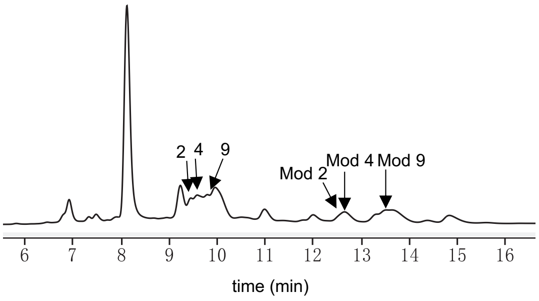

Supplementary Figure 3. LC-MS analysis of the conjugation reaction on peptide mixture RWYFLHQM_1HHWG.

Conjugation conditions: Following the standard conditions, the mixture was vortexed and shaken for 4 hours at 37 °C.

Preferred amino acids at the M_1 site: D, S, H

2. RWYFLHQSHHM_2G

Supplementary Table 4: Sequences included in the test.

16	RWYFLHQSHHAG	21	RWYFLHQSHHKG	26	RWYFLHQSHHQG
17	RWYFLHQSHHDG	22	RWYFLHQSHHMG	27	RWYFLHQSHHYG
18	RWYFLHQSHHEG	23	RWYFLHQSHHPG	28	RWYFLHQSHHWG
19	RWYFLHQSHHHG	24	RWYFLHQSHHSG	29	RWYFLHQSHHIG
20	RWYFLHQSHHLG	25	RWYFLHQSHHNG	30	RWYFLHQSHHRG

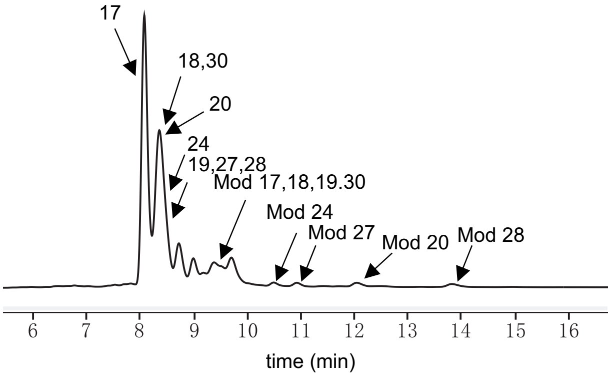

Supplementary Figure 4. LC–MS analysis of the conjugation reaction on peptide mixture RWYFLHQSHHM_2G.

Conjugation conditions: Following the standard conditions, the mixture was vortexed and shaken for 2 hours at 37 °C.

Preferred amino acids at the M_2 site: D, E, H, L, S, Y, W, R

3. RWYFLHQSM_3HWG

Supplementary Table 5: Sequences included in the test.

31	RWYFLHQSAHWG	36	RWYFLHQSKHWG	41	RWYFLHQSQHWG
32	RWYFLHQSDHWG	37	RWYFLHQSMHWG	42	RWYFLHQSYHWG
33	RWYFLHQSEHWG	38	RWYFLHQSPHWG	43	RWYFLHQSWHWG
34	RWYFLHQSHHWG	39	RWYFLHQSSHWG	44	RWYFLHQSIHWG
35	RWYFLHQSLHWG	40	RWYFLHQSNHWG	45	RWYFLHQSRHWG

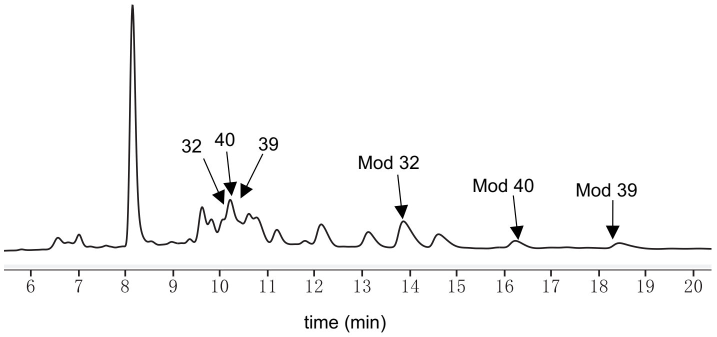

Supplementary Figure 5. LC–MS analysis of the conjugation reaction on peptide mixture RWYFLHQSM_3HWG.

Conjugation conditions: Following the standard conditions, the mixture was vortexed and shaken for 2 hours at 37 °C.

Preferred amino acids at the M_3 site: D, N, S

RWYFLHM_4SHHWG

Supplementary Table 6: Sequences included in the test.

46	RWYFLHASHHWG	51	RWYFLHKSHHWG	56	RWYFLHQSHHWG
47	RWYFLHDSHHWG	52	RWYFLHSSHHWG	57	RWYFLHYSHHWG
48	RWYFLHESHHWG	53	RWYFLHPSHHWG	58	RWYFLHWSHHWG
49	RWYFLHHSHHWG	54	RWYFLHMSHHWG	59	RWYFLHISHHWG
50	RWYFLHLSHHWG	55	RWYFLHNSHHWG	60	RWYFLHRSHHWG

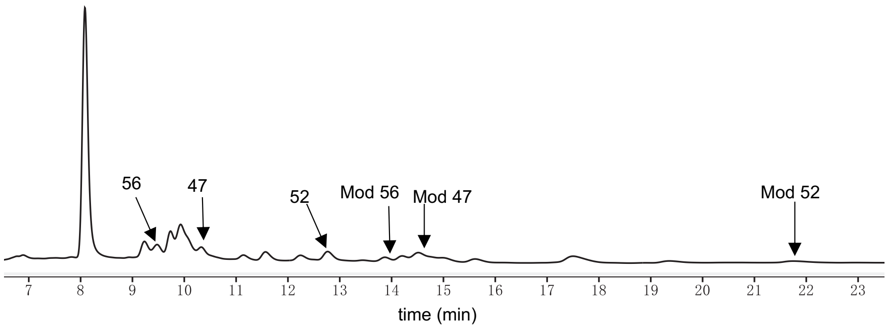

Supplementary Figure 6. LC–MS analysis of the conjugation reaction on peptide mixture RWYFLHM_4SHHWG.

Conjugation conditions: Following the standard conditions, the mixture was vortexed and shaken for 2 hours at 37 °C.

Preferred amino acids at the M_4 site: Q, D, S

4. RWYFLM_5QSHHWG

Supplementary Table 7: Sequences included in the test.

61	RWYFLAQSHHWG	66	RWYFLKQSHHWG	71	RWYFLQQSHHWG
62	RWYFLDQSHHWG	67	RWYFLMQSHHWG	72	RWYFLYQSHHWG
63	RWYFLEQSHHWG	68	RWYFLPQSHHWG	73	RWYFLWQSHHWG
64	RWYFLHQSHHWG	69	RWYFLSQSHHWG	74	RWYFLIQSHHWG
65	RWYFLLQSHHWG	70	RWYFLNQSHHWG	75	RWYFLRQSHHWG

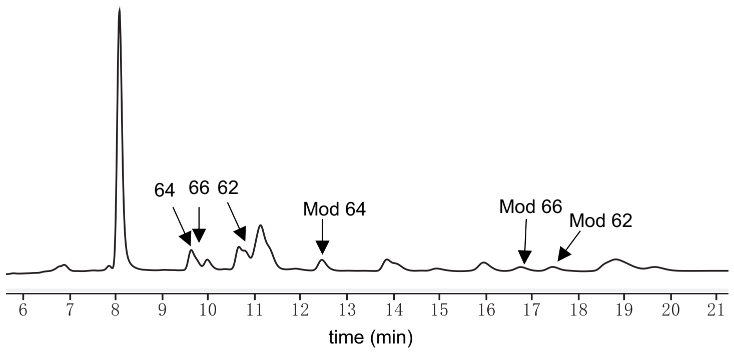

Supplementary Figure 7. LC–MS analysis of the conjugation reaction on peptide mixture RWYFLM_5QSHHWG.

Conjugation conditions: Following the standard conditions, the mixture was vortexed and shaken for 2 hours at 37 °C.

Preferred amino acids at the M_5 site: D, K, H

c. Prefered amino acids at M_1-M_5 were combined to screen for peptides that have improved yields

According to the mixed peptide screening results described above, the preferred amino acids at the M_1-M_5 sites were combined, and five peptides were synthesized to test the conjugation yields.

Supplementary Table 8: Peptide sequences tested and the conjugation yields.

Sequence	3 h	6 h	18 h
RWYFLHDSDHWG	30%	40%	40%
RWYFLKMSNHWG	5%	10%	30%
RWYFLHDHDHWG	5%	5%	5%
RWYFLKQSDHWG	72%	84%	84%
RWYFLDSDHHWG	30%	50%	50%

Conjugation conditions: Boronic acid **1** (1 µL of 50 mM stock solution in DMSO, 0.5 mM final concentration) and $CuCl_2 \cdot 2H_2O$ (1 µL of 5 mM stock solution in water, 0.05 mM final concentration) were subsequently added to a solution of peptide (1 µL of 5 mM stock solution in water, 0.05 mM final concentration) in NMM buffer (97 µL of 5 mM stock solution, pH 7.4). The mixture was vortexed and shaken at 37 °C. Reaction yields were monitored at different time points.

Supplementary Table 9: Further reaction characterizations of peptide RWYFLKQSDHWG**.**

RWYFLKQSDHWG			
	4 °C	r.t.	37 °C
30 minutes	20%	33%	58%
1 hour	24%	46%	60%
2 hours	36%	56%	65%
3 hours	42%	58%	72%
18 hours	60%	68%	84%

Conjugation conditions: To a solution of peptide (1 µL of 5 mM stock solution in water, 0.05 mM final concentration) in NMM buffer (97 µL of 5 mM stock solution, pH 7.4), boronic acid **1** (1 µL of 50 mM stock solution in DMSO, 0.5 mM final concentration) and $CuCl_2 \cdot 2H_2O$ (1 µL of 5 mM stock solution in water, 0.05 mM final concentration) were subsequently added. The mixture was vortexed and shaken at 4 °C, 25 °C, and 37 °C. The reaction yields were monitored at different time points.

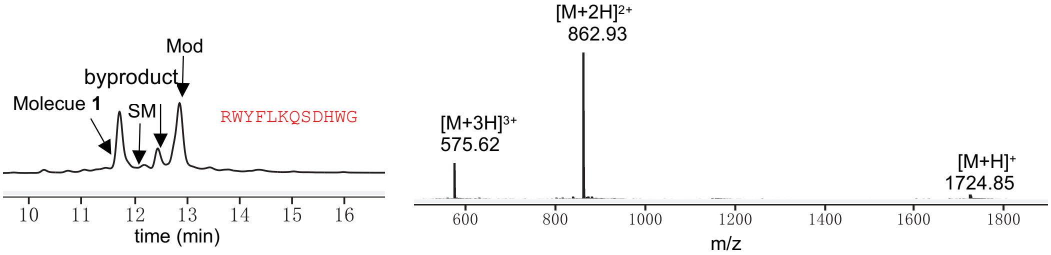

Supplementary Figure 8. LC–MS analysis of the conjugation reaction on peptide RWYFLKQSDHWG at 37 °C for 3 hours following the above-described conditions.

Supplementary Table 10. Positional scan with single mutation at P_6, P_7, P_8, P_9, P_{11} of RWYFL$P_6P_7P_8P_9$HP_{11}G based on peptide RWYFLKQSDHGW

Sequence	Yield%	Sequence	Yield%
RWYFLKQSDHDG	20%	RWYFLKQSDHWG	60%
RWYFLKQSDHEG	65%	RWYFLKESDHWG	40%
RWYFLKQSDHLG	65%	RWYFLKLSDHWG	60%
RWYFLKQSDHSG	60%	RWYFLKWSDHWG	75%
RWYFLKQSDHYG	62%	RWYFLKQDDHWG	80%
RWYFLKQSDHMG	56% Messy	RWYFLKQHDHWG	Trace
RWYFLKQSDHNG	60%	RWYFLKQNDHWG	30%
RWYFLKQSDHQG	65%	RWYFLKQQDHWG	Trace
RWYFLDQSDHWG	75%	RWYFLKQTDHWG	50%
RWYFLHQSDHWG	45%	RWYFLKQEDHWG	Trace
RWYFLEQSDHWG	75%	RWYFLKQLDHWG	Trace
RWYFLQQSDHWG	75%	RWYFLKQWDHWG	Trace
RWYFLNQSDHWG	75%	RWYFLKQSNHWG	Trace
RWYFLRQSDHWG	75%	RWYFLKQSHHWG	45%
RWYFLLQSDHWG	25%	RWYFLKQSQHWG	Trace
RWYFLWQSDHWG	25%	RWYFLKQSRHWG	Trace
RWYFLKDSDHWG	25%	RWYFLKQSEHWG	Trace
RWYFLKMSDHWG	56% Messy	RWYFLKQSSHWG	Trace
RWYFLKKSDHWG	50%	RWYFLKQSLHWG	Trace
RWYFLKNSDHWG	16%	RWYFLKQSWHWG	Trace

Conjugation conditions: To a solution of peptide (1 µL of 5 mM stock solution in water, 0.05 mM final concentration) in NMM buffer (97 µL of 5 mM stock solution, pH 7.4), boronic acid **1** (1 µL of 50 mM stock solution in DMSO, 0.5 mM final concentration) and $CuCl_2 \cdot 2H_2O$ (1 µL of 5 mM stock solution in water, 0.05 mM final concentration) were subsequently added. The mixture was vortexed and shaken for 2 hours at 25 °C.

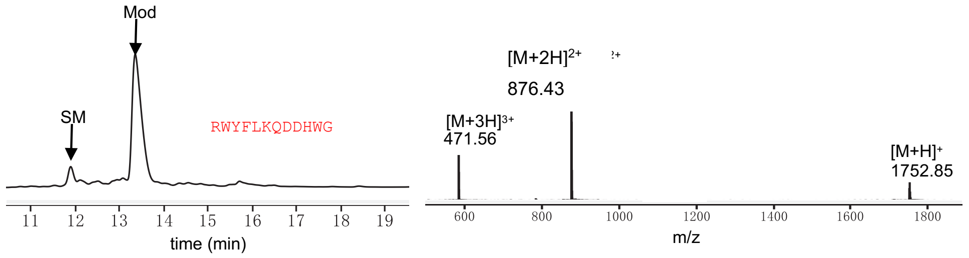

Supplementary Figure 9. LC-MS analysis of the conjugation reaction on peptide RWYFLKQDDHWG at 25 °C for 2 hours following the above-described conditions.

Supplementary Table 11. Positional scan with single mutations at P_7 of RWYFLKP_7DDHWG

Sequence	**Yield%**
RWYFLKDDDHWG	55%
RWYFLKMDDHWG	35%
RWYFLKKDDHWG	75%
RWYFLKQDDHWG	65%
RWYFLKLDDHWG	40%
RWYFLKGDDHWG	75%
RWYFLKPDDHWG	60%
RWYFLKNDDHWG	75%
RWYFLKEDDHWG	45%
RWYFLKSDDHWG	75%

Conjugation conditions: Boronic acid **1** (2 μL of 50 mM stock solution in DMSO, 1 mM final concentration) and $CuCl_2 \cdot 2H_2O$ (2 μL of 5 mM stock solution in water, 0.1 mM final concentration) were subsequently added to a solution of peptide (2 μL of 5 mM stock solution in water, 0.1 mM final concentration) in NMM buffer (94 μL of 5 mM stock solution, pH 7.4). The mixture was vortexed and shaken for 1 hour at 25 °C.

Supplementary Table 12. Alanine mutation scan of peptide RWYFLKGDDHWG

Sequence	**Yield%**
RWYFLKGDDHWG	75%
RWYFLKGDAHWG	0%.
RWYFLKGADHWG	0%.
RWYFLKADDHWG	61%
RWYFLAGDDHWG	0%.
RWYFLKGDDAWG	0%.
RWYFLKGDDHAG	92%
RWYFLKGDDHWA	29%

Conjugation conditions: To a solution of peptide (1 μL of 5 mM stock solution in water, 0.05

mM final concentration) in NMM buffer (97 µL of 5 mM stock solution, pH 7.4), boronic acid **1** (1 µL of 50 mM stock solution in DMSO, 0.5 mM final concentration) and $CuCl_2{\cdot}2H_2O$ (1 µL of 5 mM stock solution in water, 0.05 mM final concentration) were added subsequently. The mixture was vortexed and shaken for 1 hour at 37 °C.

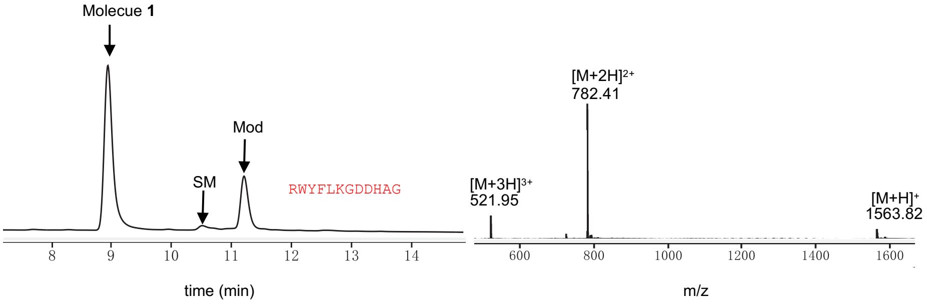

Supplementary Figure 10. LC-MS analysis of the conjugation reaction on peptide RWYFLKGDDHAG at 37 °C for 1 hour following the above-described conditions.

Supplementary Table 13. Positional scan with single mutations at P_{11}, and P_{12} of RWYFLKGDDH$P_{11}P_{12}$ based on peptide RWYFLKGDDHAG

Sequence	Yield%	Sequence	Yield%
RWYFLKGDDHAA	95%	RWYFLKGDDHIG	92%
RWYFLKGDDHAI	93%	RWYFLKGDDHLG	94%
RWYFLKGDDHAL	90%	RWYFLKGDDHMG	93%
RWYFLKGDDHAV	13%	RWYFLKGDDHVG	0%
RWYFLKGDDHAF	5%	RWYFLKGDDHFG	22%
RWYFLKGDDHAW	60%	RWYFLKGDDHYG	90%
RWYFLKGDDHAY	12%	RWYFLKGDDHNG	93%
RWYFLKGDDHAN	93%	RWYFLKGDDHQG	92%
RWYFLKGDDHAQ	45%	RWYFLKGDDHSG	90%
RWYFLKGDDHAS	82%	RWYFLKGDDHTG	92%
RWYFLKGDDHAT	57%	RWYFLKGDDHDG	90%
RWYFLKGDDHAD	92%	RWYFLKGDDHRG	10%
RWYFLKGDDHAE	41%	RWYFLKGDDHHG	1%
RWYFLKGDDHAR	92%	RWYFLKGDDHKG	91%
RWYFLKGDDHAH	2%	RWYFLKGDDHPG	68%
RWYFLKGDDHAK	75%	RWYFLKGDDHGG	45%

Conjugation conditions: To a solution of peptide (1 µL of 5 mM stock solution in water, 0.05 mM final concentration) in NMM buffer (97 µL of 5 mM stock solution, pH 7.4), boronic acid **1** (1 µL of 50 mM stock solution in DMSO, 0.5 mM final concentration) and $CuCl_2{\cdot}2H_2O$ (1 µL of 5 mM stock solution in water, 0.05 mM final concentration) were added subsequently. The mixture was vortexed and shaken for 1 hour at 37 °C.

Supplementary Table 14. Positional scan with single mutations at P_4, P_5, P_6, and P_7 of RWY$P_4P_5P_6P_7$HAA based on peptide RWYFLKKDDHAA

Sequence	**Yield%**	Sequence	**Yield%**
RWYDLKKDDHAA	25%	RWYFLDKDDHAA	85%
RWYELKKDDHAA	5%	RWYFLEKDDHAA	88%
RWYKLKKDDHAA	67%	RWYFLFKDDHAA	80%
RWYHLKKDDHAA	80%	RWYFLKKDDHAA	85%
RWYILKKDDHAA	72%	RWYFLHKDDHAA	86%
RWYMLKKDDHAA	46%	RWYFLIKDDHAA	84%
RWYNLKKDDHAA	27%	RWYFLMKDDHAA	50%
RWYPLKKDDHAA	21%	RWYFLNKDDHAA	76%
RWYQLKKDDHAA	39%	RWYFLPKDDHAA	76%
RWYRLKKDDHAA	89%	RWYFLQKDDHAA	64%
RWYSLKKDDHAA	70%	RWYFLRKDDHAA	88%
RWYWLKKDDHAA	87%	RWYFLSKDDHAA	42%
RWYYLKKDDHAA	70%	RWYFLKADDHAA	70%
RWYFAKKDDHAA	50%	RWYFLKDDDHAA	30%
RWYFDKKDDHAA	66%	RWYFLKEDDHAA	60%
RWYFFKKDDHAA	89%	RWYFLKFDDHAA	80%
RWYFKKKDDHAA	70%	RWYFLKHDDHAA	15%
RWYFHKKDDHAA	87%	RWYFLKIDDHAA	65%
RWYFMKKDDHAA	80%	RWYFLKKDDHAA	87%
RWYFNKKDDHAA	75%	RWYFLKMDDHAA	50%
RWYFPKKDDHAA	38%	RWYFLKNDDHAA	35%
RWYFQKKDDHAA	50%	RWYFLKPDDHAA	85%
RWYFRKKDDHAA	70%	RWYFLKQDDHAA	93%
RWYFSKKDDHAA	43%	RWYFLKRDDHAA	75%
RWYFWKKDDHAA	40%	RWYFLKSDDHAA	88%
RWYFYKKDDHAA	92%	RWYFLKWDDHAA	66%
RWYFLAKDDHAA	88%	RWYFLKYDDHAA	87%

Conjugation conditions: To a solution of peptide (1 µL of 5 mM stock solution in water, 0.05 mM final concentration) in NMM buffer (97 µL of 5 mM stock solution, pH 7.4), boronic acid **1** (1 µL of 50 mM stock solution in DMSO, 0.5 mM final concentration) and $CuCl_2 \cdot 2H_2O$ (1 µL of 5 mM stock solution in water, 0.05 mM final concentration) were added subsequently. The mixture was vortexed and shaken for 30 minutes at 25 °C.

Supplementary Table 15. Positional scan with single mutations at P_8, P_9 of RWYFLKKP_8P_9HAA based on peptide RWYFLKKDDHAA

Sequence	Yield%	Sequence	Yield%
RWYFLKKADHAA	0%	RWYFLKKDAHAA	5%
RWYFLKKEDHAA	trace	RWYFLKKDEHAA	10%
RWYFLKKFDHAA	0%	RWYFLKKDFHAA	0%
RWYFLKKKDHAA	0%	RWYFLKKDKHAA	0%
RWYFLKKHDHAA	2%	RWYFLKKDHHAA	40%
RWYFLKKIDHAA	0%	RWYFLKKDIHAA	0%
RWYFLKKKDHAA	0%	RWYFLKKDKHAA	0%
RWYFLKKMDHAA	0%	RWYFLKKDMHAA	0%
RWYFLKKNDHAA	72%	RWYFLKKDNHAA	16%
RWYFLKKPDHAA	0%	RWYFLKKDPHAA	0%
RWYFLKKQDHAA	0%	RWYFLKKDQHAA	0%
RWYFLKKRDHAA	0%	RWYFLKKDRHAA	0%
RWYFLKKSDHAA	75%	RWYFLKKDSHAA	14%
RWYFLKKWDHAA	0%	RWYFLKKDWHAA	0%
RWYFLKKYDHAA	0%	RWYFLKKDYHAA	0%

Conjugation conditions: To a solution of peptide (1 μL of 5 mM stock solution in water, 0.05 mM final concentration) in NMM buffer (97 μL of 5 mM stock solution, pH 7.4), boronic acid **1** (1 μL of 50 mM stock solution in DMSO, 0.5 mM final concentration) and $CuCl_2·2H_2O$ (1 μL of 5 mM stock solution in water, 0.05 mM final concentration) were added subsequently. The mixture was vortexed and shaken for 30 minutes at 25 °C.

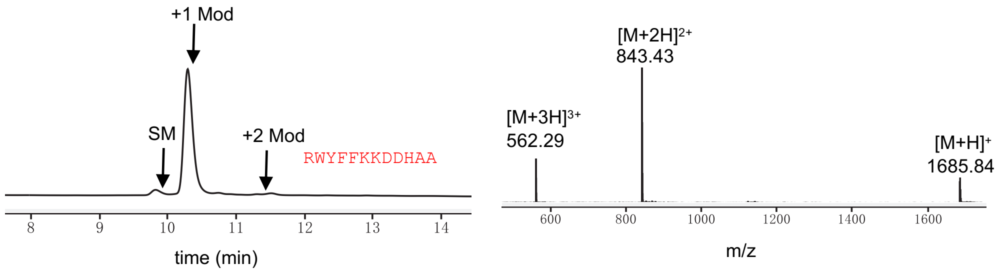

Supplementary Figure 11. LC-MS analysis of the conjugation reaction on peptide RWYFFKKDDHAA.

Conjugation conditions: To a solution of peptide (1 μL of 5 mM stock solution in water, 0.05 mM final concentration) in NMM buffer (97 μL of 5 mM stock solution, pH 7.4), boronic acid **1** (1 μL of 50 mM stock solution in DMSO, 0.5 mM final concentration) and $CuCl_2·2H_2O$ (1 μL of 15 mM stock solution in water, 0.15 mM final concentration) were added subsequently. The mixture was vortexed and shaken for 15 minutes at 25 °C.

3. Experimental procedures and reaction optimizations

a. General reaction conditions:

General reaction conditions: To a solution of peptide (1 μL of 5 mM stock solution in water, 0.05 mM final concentration) in NMM buffer (97 μL of 50 mM stock solution, pH 7.4), boronic acid listed below (1 μL of 50 mM stock solution in DMSO, 0.5 mM final concentration) and $CuCl_2 \cdot 2H_2O$ (1 μL of 15 mM stock solution in water, 0.15 mM final concentration) were subsequently added. The mixture was vortexed and shaken for 15 minutes at 37 °C. Na_4-EDTA (2 μL of 500 mM stock solution in H_2O, 20 mM final concentration) was added to quench the reactions. Then, the crude reaction mixture was centrifuged to remove any precipitates, and the supernatant was analyzed by LC–MS to determine the reaction yield.

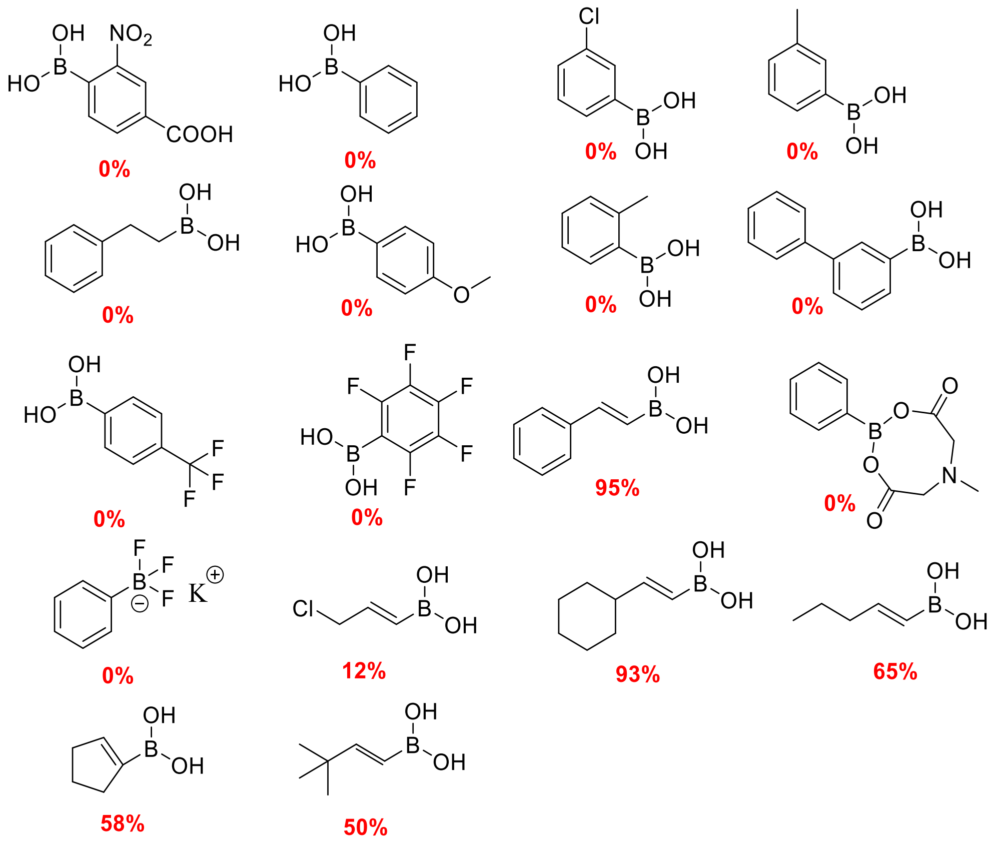

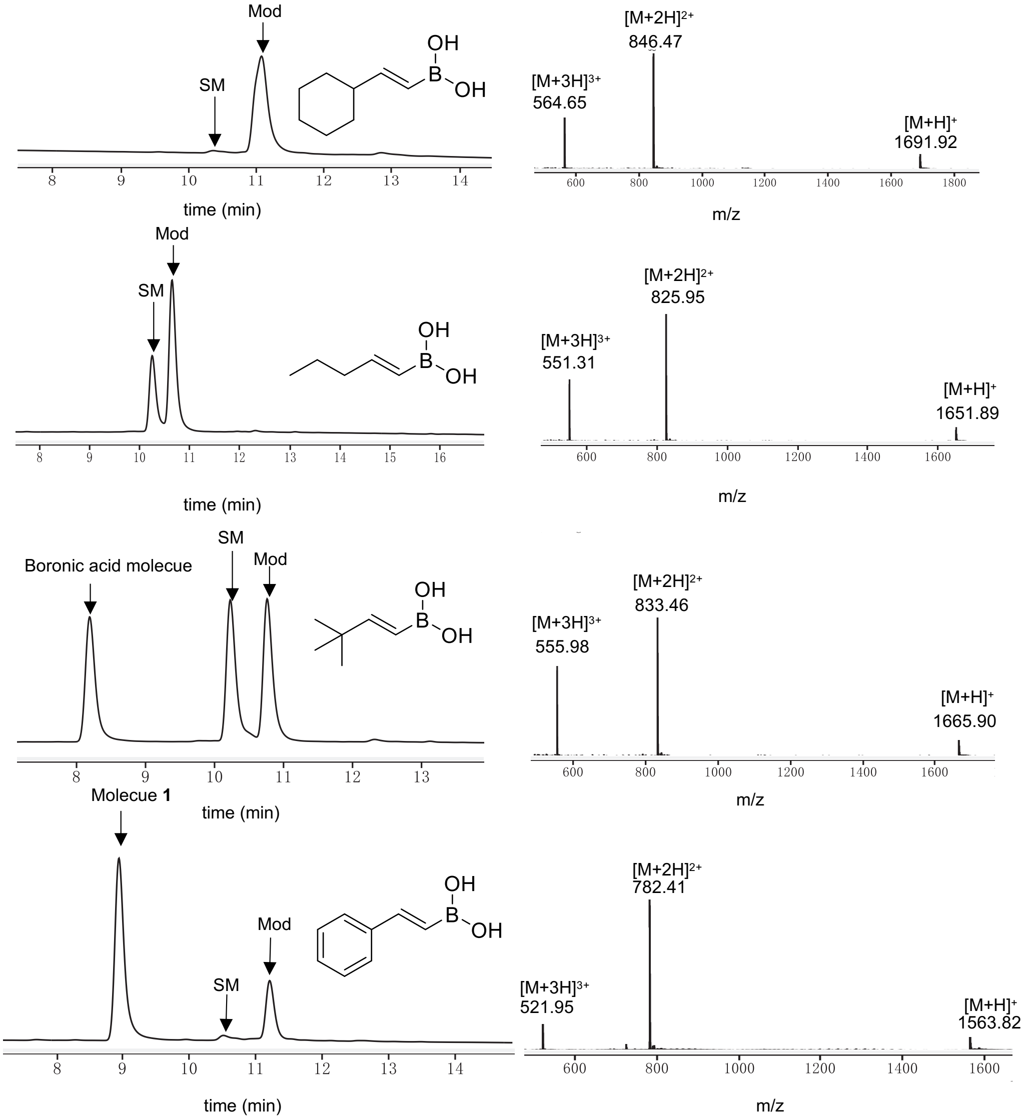

Supplementary Figure 12. The screen of boronic acid reagent reactions with peptide RWYFFKKDDHAA. Conjugation conditions: Boronic acid reagent (1 μL of 50 mM stock solution in DMSO, 0.5 mM final concentration) and $CuCl_2 \cdot 2H_2O$ (1 μL of 15 mM stock solution in water, 0.15 mM final concentration) were subsequently added to a solution of peptide RWYFFKKDDHAA (1 μL of 5 mM stock solution in water, 0.05 mM final concentration) in NMM buffer (97 μL of 50 mM stock solution, pH 7.4). The mixture was vortexed and shaken for 15 minutes at 37 °C.

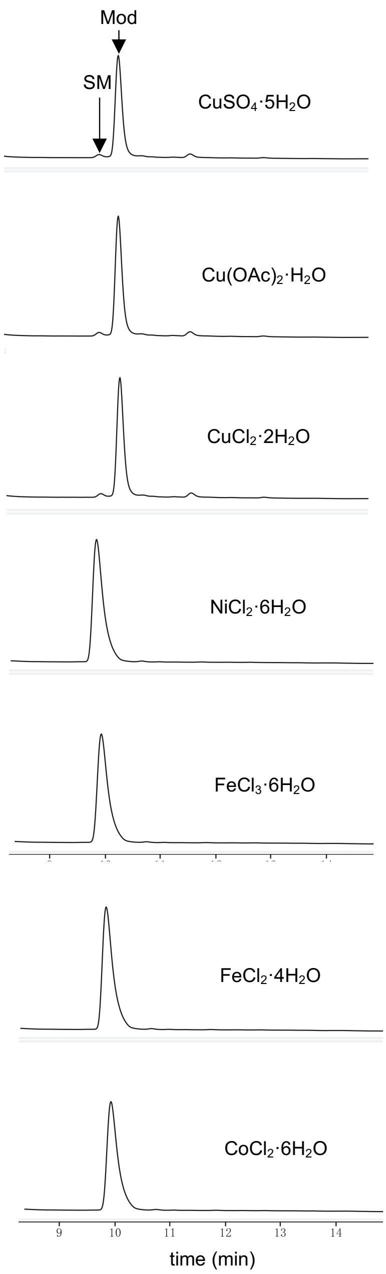

Supplementary Figure 13. Screening of other transition metal ions. Cu^{2+} is capable of mediating the reaction efficiently, and Fe^{3+}, Ni^{2+}, Fe^{2+} and Co^{2+} are not suitable for this reaction. Conjugation conditions: Boronic acid **1** (1 µL of 50 mM stock solution in DMSO, 0.5 mM final concentration) and different metal ions (1 µL of 15 mM stock solution in water, 0.15 mM final concentration) were subsequently added to a solution of peptide `RWYFFKKDDHAA` (1 µL of 5 mM stock solution in water, 0.05 mM final concentration) in NMM buffer (97 µL of 50 mM stock solution, pH 7.4). The mixture was vortexed and shaken for 15 minutes at 37 °C.

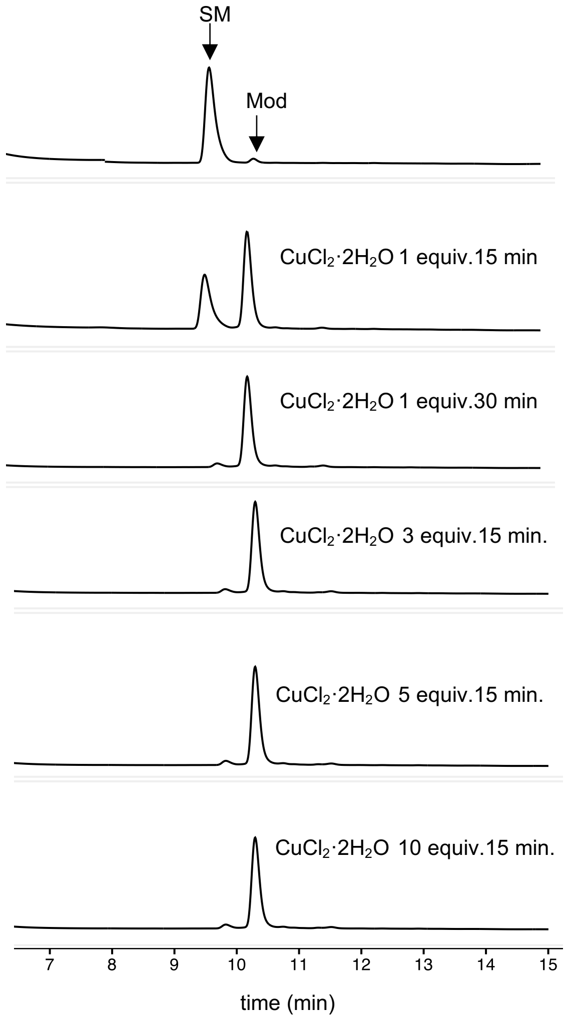

Supplementary Figure 14. Screening of $CuCl_2 \cdot 2H_2O$ ratios. Conjugation conditions: To a solution of peptide RWYFFKKDDHAA (1μL of 5 mM stock solution in water, 0.05 mM final concentration) in NMM buffer (97 μL of 50 mM stock solution, pH 7.4), boronic acid **1** (1 μL of 50 mM stock solution in DMSO, 0.5 mM final concentration) and $CuCl_2 \cdot 2H_2O$ (0.025 mM, 0.05 mM, 0.15 mM, 0.25 mM, 0.5 mM final concentration) were added subsequently. The mixture was vortexed and shaken for 15 minutes at 37 °C.

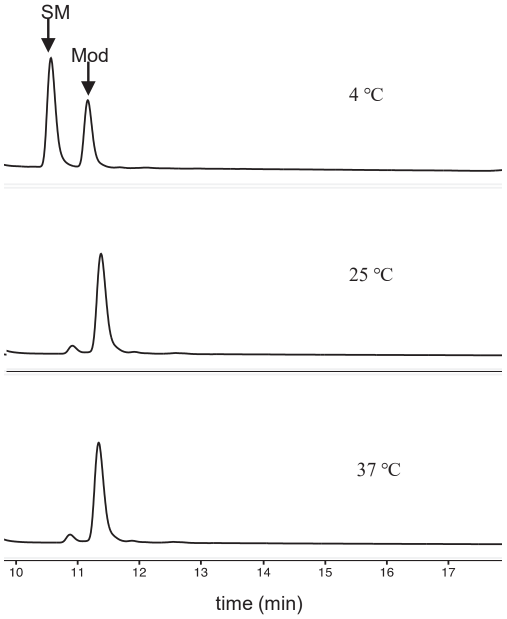

Supplementary Figure 15. Screening of temperature. Conjugation conditions: To a solution of peptide `RWYFFKKDDHAA` (1 µL of 5 mM stock solution in water, 0.05 mM final concentration) in NMM buffer (97 µL of 50 mM stock solution, pH 7.4), boronic acid **1** (1 µL of 50 mM stock solution in DMSO, 0.5 mM final concentration) and $CuCl_2 \cdot 2H_2O$ (1 µL of 15 mM stock solution in water, 0.15 mM final concentration) were added subsequently. The mixture was vortexed and shaken for 15 minutes at 4 °C, 25 °C, 37 °C.

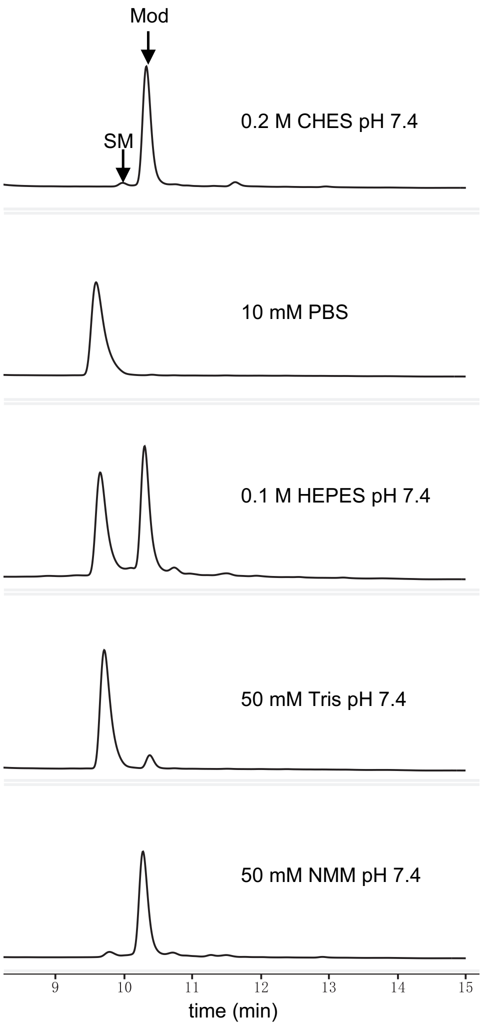

Supplementary Figure 16. **Screening different buffers. Conjugation conditions:** Boronic acid **1** (1 µL of 50 mM stock solution in DMSO, 0.5 mM final concentration) and $CuCl_2 \cdot 2H_2O$ (1 µL of 15 mM stock solution in water, 0.15 mM final concentration) were subsequently added to a solution of peptide `RWYFFKKDDHAA` (1 µL of 5 mM stock solution in water, 0.05 mM final concentration) in various buffers (97 µL of various buffers). The mixture was vortexed and shaken for 15 minutes at 37 °C.

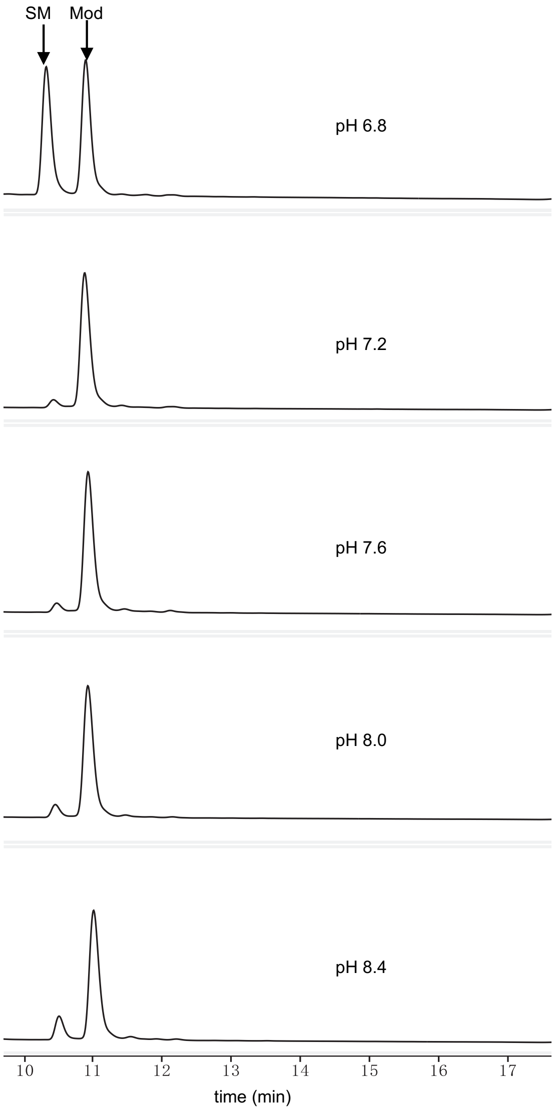

Supplementary Figure 17. **Screening of pH. Conjugation conditions:** Boronic acid **1** (1 µL of 50 mM stock solution in DMSO, 0.5 mM final concentration) and $CuCl_2{\cdot}2H_2O$ (1 µL of 15 mM stock solution in water, 0.15 mM final concentration) were subsequently added to a solution of peptide `RWYFFKKDDHAA` (1 µL of 5 mM stock solution in water, 0.05 mM final concentration) in NMM buffer (97 µL of 50 mM stock solution, pH 6.8, 7.2, 7.6, 8.0 8.4). The mixture was vortexed and shaken for 15 minutes at 37 °C.

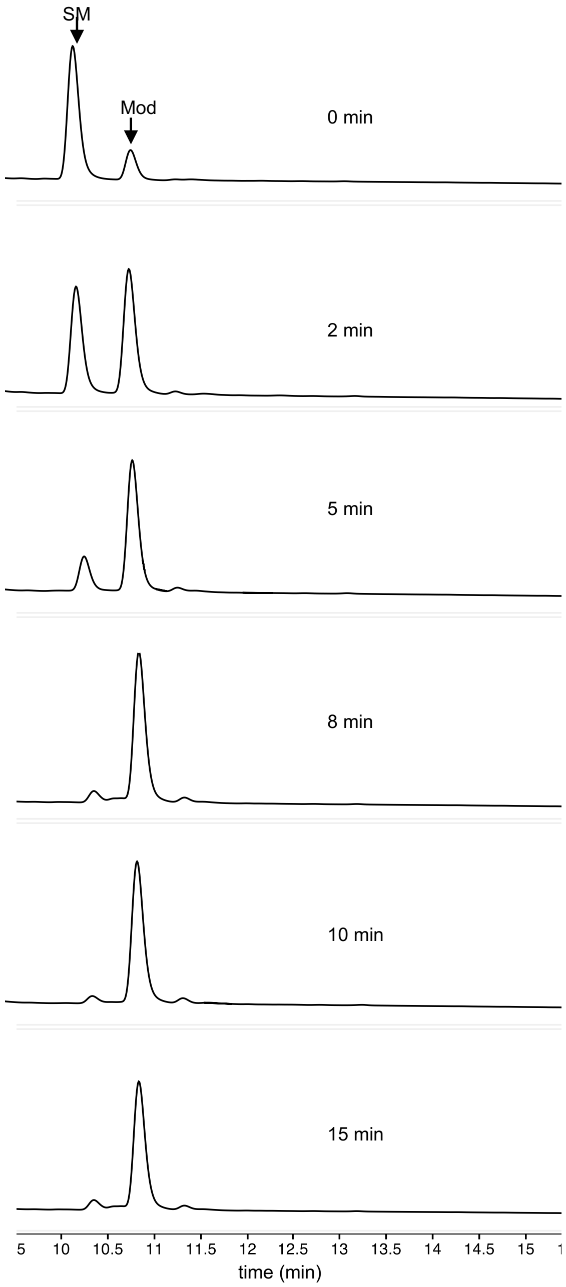

Supplementary Figure 18. Peptide `RWYFFKKDDHAA` reaction kinetics. Conjugation conditions: To a solution of peptide `RWYFFKKDDHAA` (1 µL of 5 mM stock solution in water, 0.05 mM final concentration) in NMM buffer (97 µL of 50 mM stock solution, pH 7.4), boronic acid **1** (1 µL of 50 mM stock solution in DMSO, 0.5 mM final concentration) and $CuCl_2 \cdot 2H_2O$ (1 µL of 15 mM stock solution in water, 0.15 mM final concentration) were added subsequently. The mixture was vortexed and shaken for 0 minutes, 2 minutes, 5 minutes, 8 minutes, 10 minutes, and 15 minutes at 37 °C.

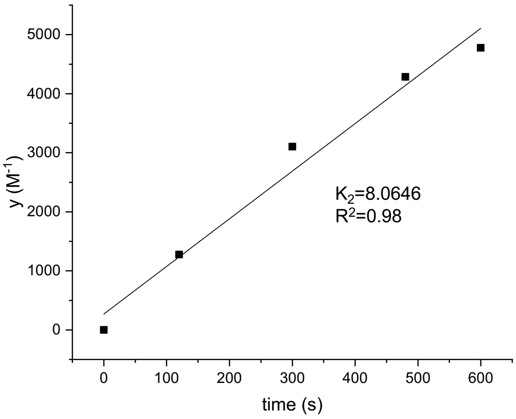

Supplementary Figure 19. Linear fitting of kinetics data for *CAST* peptide reaction. We assume Cu(II) binding to peptide is much faster than the conjugation reaction, therefore, we assume the reaction is pseudosecond order reaction between Cu((II) bound peptide and styrylboronic acid. The second-order reaction rate was determined by fitting the data to the following equation[9]:

$$\mathrm{y} = \frac{\ln\left(\frac{[peptide]_0[probe]_t}{[peptide]_t[probe]_0}\right)}{([probe]_0 - [peptide]_0)} = k_2 t$$

t= 0 s, 120 s, 300 s, 480 s, 600 s. $[\text{peptide}]_0$ and $[\text{probe}]_0$ are the initial concentrations of the peptide and the probe; and $[\text{peptide}]_t$ and $[\text{probe}]_t$ are the concentrations of the peptide and the probe at time t.

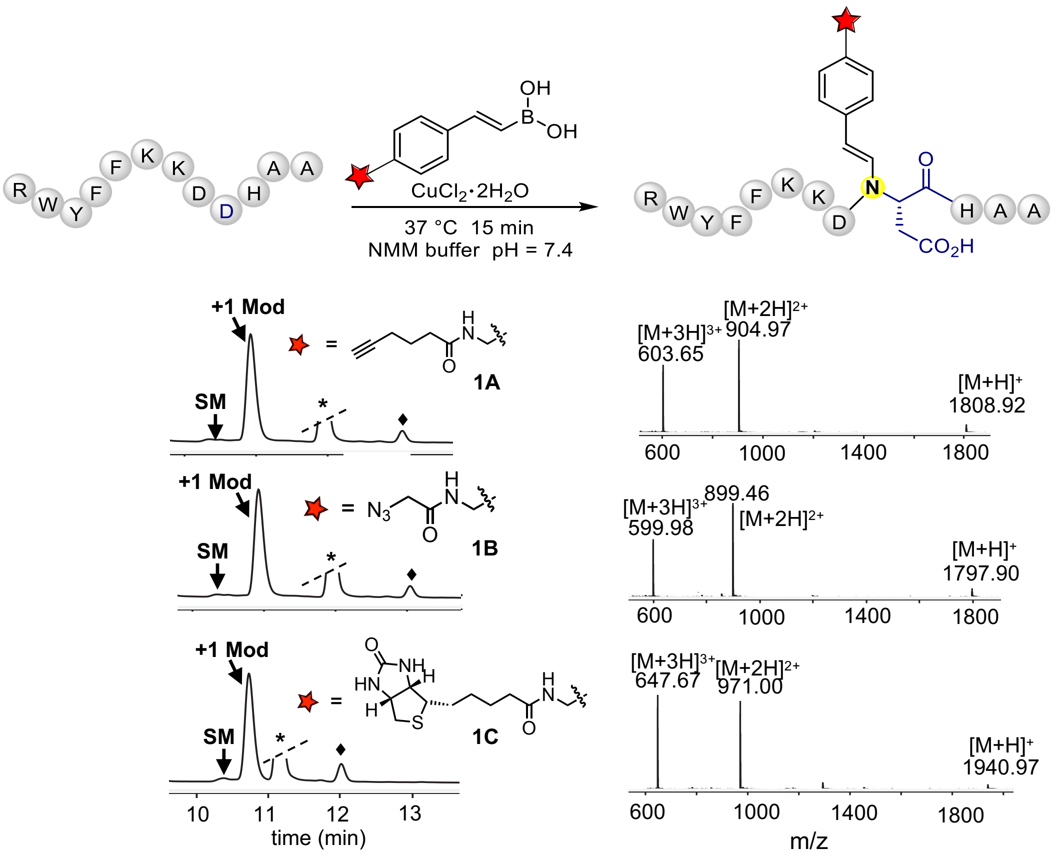

Supplementary Figure 20. Peptide RWYFFKKDDHAA reactions with boronic acid derivatives. Left: HPLC analysis of the conjugation reaction; Right, mass-spectrum of the corresponding modified product. Peaks marked at *, ♦ are non-peptide byproducts from **1A**, **1B** or **1C**. **Conjugation conditions:** To a solution of peptide RWYFFKKDDHAA (1µL of 5 mM stock solution in water, 0.05 mM final concentration) in NMM buffer (96 µL of 50 mM stock solution, pH 7.4,), boronic acid derivatives (2 µL of 25 mM stock solution in DMSO, 0.5 mM final concentration) and $CuCl_2 \cdot 2H_2O$ (1 µL of 15 mM stock solution in water, 0.15 mM final concentration) were subsequently added. The mixture was incubated for 15 minutes at 37 °C.

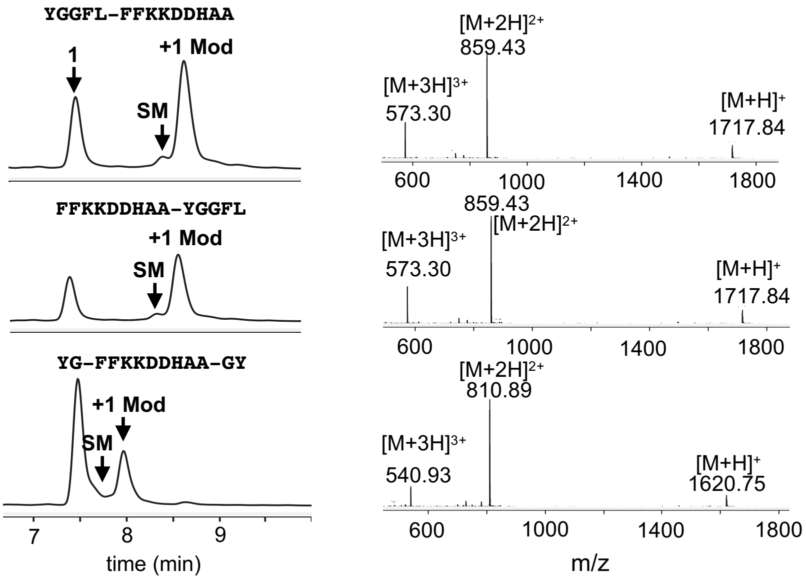

Supplementary Figure 21. **Screening of *CAST* positions.** Left: HPLC analysis of the conjugation reaction; Right, mass-spectrum of the corresponding modified product. CAST-tag was inserted into the C-terminal, N-terminal or in the middle of a peptide. Conjugation conditions：To a solution of peptide (1μL of 5 mM stock solution in water, 0.05 mM final concentration) in NMM buffer (97 μL of 50 mM stock solution, pH 7.4), boronic acid **1** (1 μL of 50 mM stock solution in DMSO, 0.5 mM final concentration) and $CuCl_2{\cdot}2H_2O$ (1 μL of 15 mM stock solution in water, 0.15 mM final concentration) were subsequently added. The mixture was incubated for 15 minutes at 37 °C.

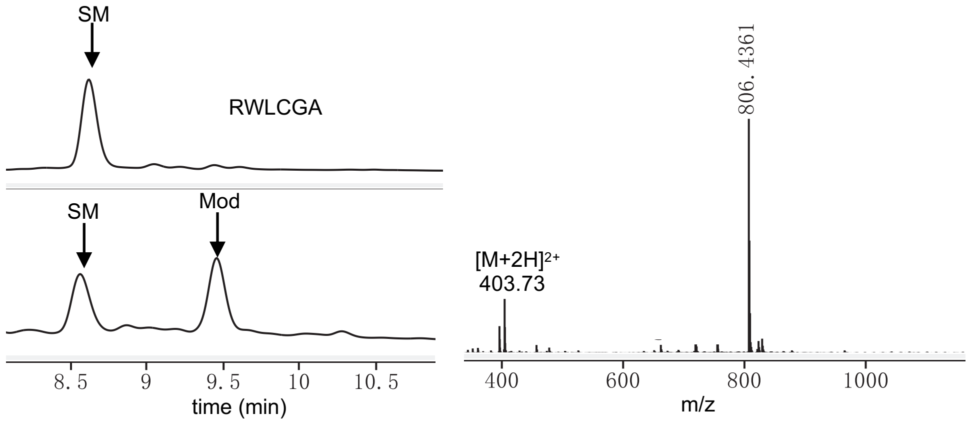

Supplementary Figure 22. The reaction of unprotected Cys and boronic acid 1. Left: HPLC analysis of the conjugation reaction; Right, mass-spectrum of the modified product. **Conjugation conditions:** To a solution of peptide RWLCGA (2 µL of 5 mM stock solution in water, 0.1 mM final concentration) in NMM buffer (94 µL of 50 mM stock solution, pH 7.4), boronic acid **1** (2 µL of 50 mM stock solution in DMSO, 1 mM final concentration) and $CuCl_2 \cdot 2H_2O$ (2 µL of 25 mM stock solution in water, 0.5 mM final concentration) were subsequently added. The mixture was incubated for 1 hour at 37 °C.

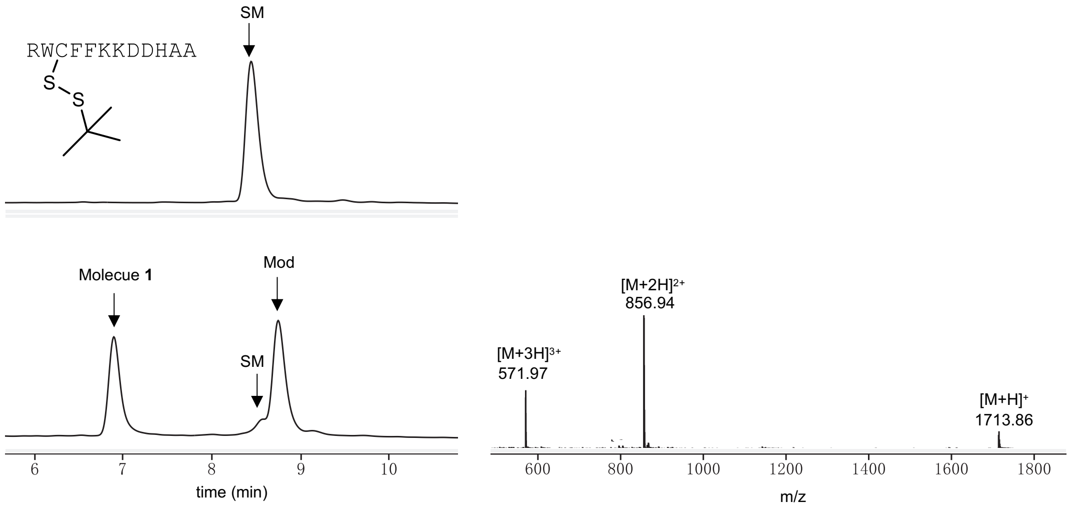

Supplementary Figure 23. The disulfide bond has no impact on *CAST* conjugation reaction. Left: HPLC analysis of the conjugation reaction; Right, mass-spectrum of the modified product. **Conjugation conditions:** To a solution of peptide (1 µL of 5 mM stock solution in water, 0.05 mM final concentration) in NMM buffer (97 µL of 50 mM stock solution, pH 7.4), boronic acid **1** (1 µL of 50 mM stock solution in DMSO, 0.5 mM final concentration) and $CuCl_2 \cdot 2H_2O$ (1 µL of 25 mM stock solution in water, 0.25 mM final concentration) were subsequently added. The mixture was incubated for 15 minutes at 37 °C.

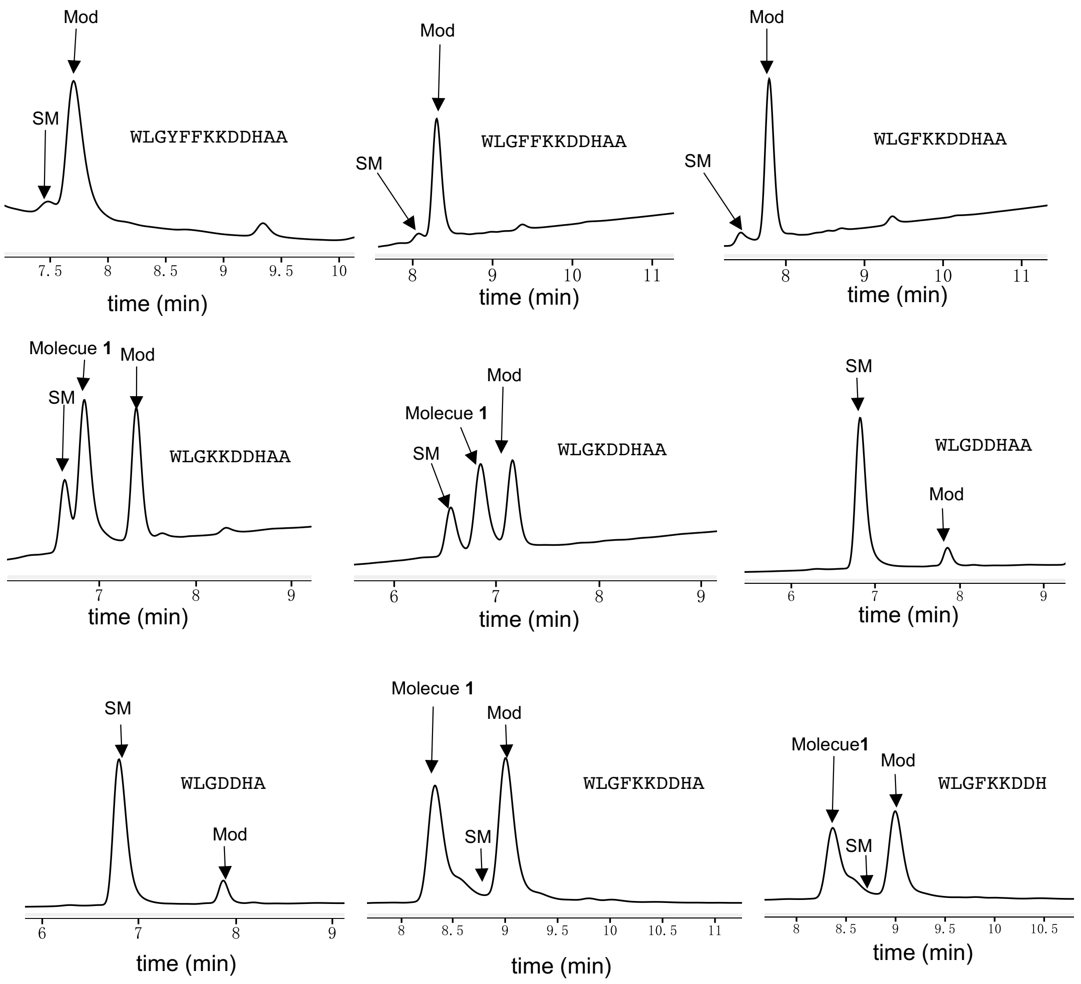

Supplementary Figure 24. **Screening of minimal sequence length**. **Conjugation conditions:** To a solution of peptide (1 µL of 5 mM stock solution in water, 0.05 mM final concentration) in NMM buffer (97 µL of 50 mM stock solution, pH 7.4), boronic acid **1** (1 µL of 50 mM stock solution in DMSO, 0.5 mM final concentration) and $CuCl_2·2H_2O$ (1 µL of 15 mM stock solution in water, 0.15 mM final concentration) were subsequently added. The mixture was incubated for 15 minutes at 37 °C.

4. Preparation of boronic acid derivatives

a. Synthesis of boronic acid derivatives

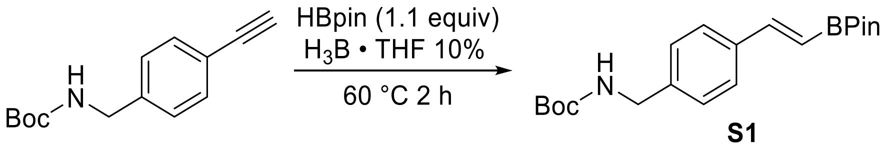

S1

tert-butyl(E)-(4-(2-(4,4,5,5-tetramethyl-1,3,2-dioxaborolan-2-yl)vinyl)benzyl) carbamate (S1). Starting material tert-butyl (E)-(4-(2-(4,4,5,5-tetramethyl-1,3,2-dioxaborolan-2-yl)vinyl)benzyl)carbamate was synthesized following literature procedures[3]. Tert-butyl (4-ethynylbenzyl)carbamate (4.00 mmol), pinacolborane (4.40 mmol, 0.640 mL) and H_3B•THF (0.40 mmol, 1 M in THF, 0.40 mL) were added sequentially to a 25 mL sealed reaction vial flushed with nitrogen, reaction mixture was stirred at 60 °C for 4 hours. Upon completion, the solvents were removed under reduced pressure. The crude reaction mixture was purified by basified flash column chromatography (SiO_2: hexane/EtOAc: 15:1~10:1, yellow oil, yield: 70%).

^{1}H NMR (600 MHz, Chloroform-*d*) δ 7.37 (d, J = 8.0 Hz, 2H), 7.30 (d, J = 18.4 Hz, 1H), 7.17 (d, J = 7.9 Hz, 2H), 6.07 (d, J = 18.4 Hz, 1H), 4.23 (d, J = 6.0 Hz, 2H), 1.38 (s, 9H), 1.24 (s, 12H). ^{13}C NMR (150 MHz, $CDCl_3$) δ 156.01, 149.14, 139.84, 136.75, 127.78, 127.42, 83.48, 79.65, 44.53, 28.53, 24.93. HRMS calculated for $C_{20}H_{31}BNO_4$ $[M+H]^+$ 360.2341, observed 361.2568.

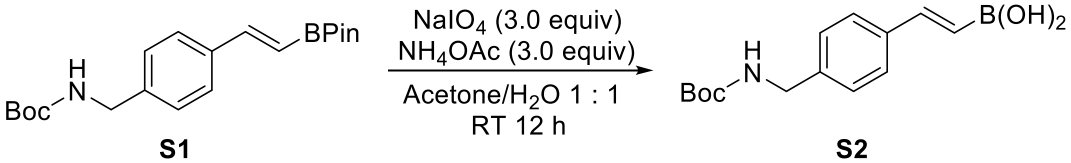

S1 **S2**

(E)-(4-(((tert-butoxycarbonyl)amino)methyl)styryl)boronic acid (S2). Tert-butyl (E)-(4-(2-(4,4,5,5-tetramethyl-1,3,2-dioxaborolan-2-yl)vinyl)benzyl)carbamate (4.00 mmol), $NaIO_4$ (12 mmol) and NH_4OAc (12 mmol) were added to reaction vial, then acetone (32 mL) and H_2O (16 mL) were added. The reaction mixture was stirred at room temperature for 12 hours, then quenched with brine and extracted with EtOAc (3×40 mL). The organic phase was collected, Na_2SO_4 was added and solvents were removed under reduced pressure. The product was used without further purification (White solid, yield: 90%).

^{1}H NMR (500 MHz, DMSO-d_6) δ 7.75 (s, 2H), 7.42 (d, J = 8.1 Hz, 2H), 7.22 (m,3H), 6.08 (d, J = 18.4 Hz, 1H), 4.11 (d, J = 6.1 Hz, 2H), 3.34 (m, 1H), 1.39 (s, 9H). ^{13}C NMR (125 MHz, DMSO-d_6) δ155.69, 145.48, 140.43, 136.06, 127.16, 126.42, 122.58, 77.69, 43.04, 28.14. HRMS calculated for $C_{14}H_{20}BNO_4$ $[M+Na]^+$ 300.1383, observed 300.1377.

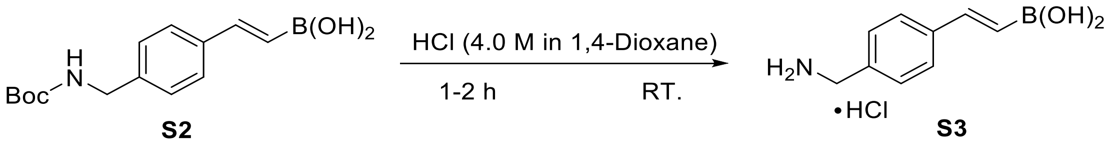

S2 **S3**

(E)-(4-(aminomethyl)styryl)boronic acid hydrochloride (S3). (E)-(4-(((tert-butoxycarbonyl)amino)methyl)styryl)boronic acid was added to reaction vial and HCl (4.0 M in 1,4-Dioxane) was added to fully dissolve the boronic acid substrate. The reaction mixture was stirred at room temperature for 1-2 hours. Upon completion, the solvents were removed under reduced pressure. (E)-(4-(aminomethyl)styryl)boronic acid hydrochloride was obtained quantitatively (White solid).

^{1}H NMR (500 MHz, DMSO-d$_6$) δ 8.51 (s, 4H), 7.81 (s, 1H), 7.50 (m, 4H), 7.26 (d, J = 18.4 Hz, 1H), 6.16 (d, J = 18.4 Hz, 1H), 4.00 (m, 2H).^{13}C NMR (125 MHz, DMSO-d$_6$) δ 144.99, 137.63, 134.03, 129.22, 126.56, 123.94, 41.74. HRMS calculated for $C_9H_{10}BO_2$ [M-NH$_2$]$^+$ 161.0768, observed 161.0757.

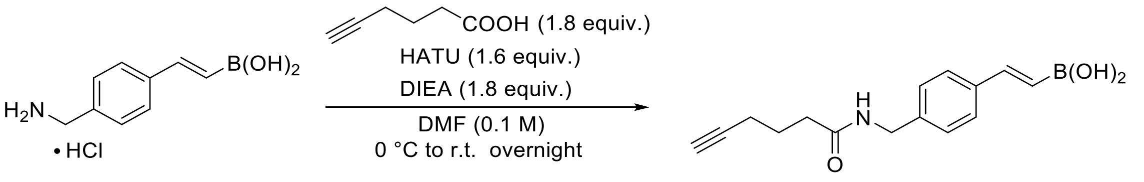

S3 1A

(E)-(4-(hex-5-ynamidomethyl)styryl)boronic acid (1A). 5-Hexynoic acid (20.2 mg, 0.18 mmol) and HATU (60.8 mg, 0.16 mmol) were dissolved in DMF (1.0 mL). *N, N*-Diisopropylethylamine (31.3 μL, 0.18 mmol) was added at 0 °C, and the mixture was stirred at room temperature for 10 minutes. The reaction was cooled by ice cold bath and **S3** (21.3 mg, 0.1 mmol) was added. The mixture was stirred at room temperature overnight, and all of the volatiles were removed by vacuum pump. The crude product was purified by reverse-phase HPLC (5.0–65% MeCN over 40 minutes). Lyophilization of collected fractions gave the product.

^{1}H NMR (500 MHz, DMSO-d$_6$) δ 8.35 (t, J = 5.9 Hz, 1H), 7.75 (s, 2H), 7.42 (d, J = 8.1 Hz, 2H), 7.21-7.24 (m, 3H), 6.08 (d, J = 18.4 Hz, 1H), 4.25 (d, J = 5.9 Hz, 2H), 2.79 (t, J = 2.6 Hz, 1H), 2.21-2.24 (m, 2H), 2.15-2.17 (m, 2H), 1.73 -1.67 (m, 2H).^{13}C NMR (125 MHz, DMSO-d$_6$) δ 171.36, 145.44, 139.90, 136.12, 127.42, 126.44, 122.65, 83.96, 71.42, 41.67, 33.97, 24.15, 17.29. HRMS calculated for $C_{15}H_{18}BNO_3$ [M+H]$^+$ 272.1413, observed 272.1474.

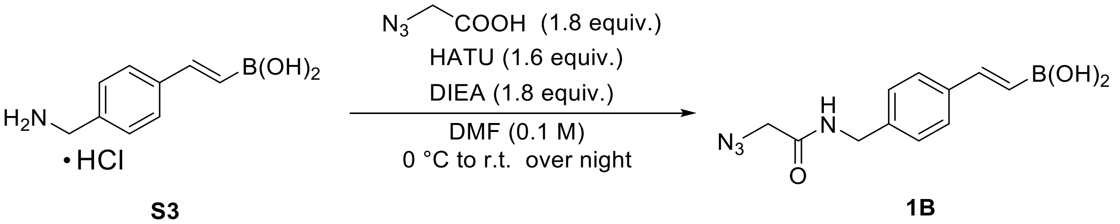

S3 1B

(E)-(4-((2-azidoacetamido)methyl)styryl)boronic acid (1B). 2-azidoacetic acid (18.2 mg, 0.18 mmol) and HATU (60.8 mg, 0.16 mmol) were dissolved in DMF (1.0 mL). *N, N*-Diisopropylethylamine (31.3 μL, 0.18 mmol) was added at 0 °C, and the mixture was stirred at room temperature for 10 minutes. The reaction was cooled by ice cold bath and **S3** (21.3 mg, 0.1 mmol) was added. The mixture was stirred at room temperature overnight, and all of the volatiles were removed by vacuum pump. The crude product was purified by reverse-phase HPLC (5.0–65% MeCN over 40 minutes). Lyophilization of collected fractions gave the

product.

^{1}H NMR (500 MHz, DMSO-d_6) δ 8.62 (t, J = 5.9 Hz, 1H), 7.76 (s, 2H), 7.43 (d, J = 8.1 Hz, 2H), 7.21-7.27 (m, 3H), 6.09 (d, J = 18.4 Hz, 1H), 4.30 (d, J = 5.9 Hz, 2H), 3.89 (s, 2H).^{13}C NMR (125 MHz, DMSO-d_6) δ 167.24, 145.37, 139.11, 136.34, 127.62, 126.49, 122.82, 50.68, 41.88. HRMS calculated for $C_{11}H_{13}BN_4O_3$ $[M+H]^+$ 261.1114, observed 261.1170.

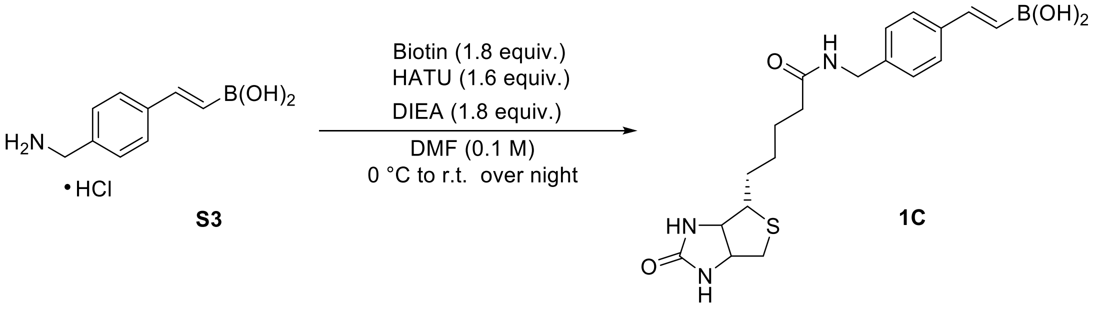

((E)-4-((5-((4S)-2-oxohexahydro-1H-thieno[3,4-d]imidazol-4-yl)pentanamido)methyl)styryl)boronic acid (1C). Biotin (44.0 mg, 0.18 mmol) and HATU (60.8 mg, 0.16 mmol) were dissolved in DMF (1.0 mL). *N, N*-Diisopropylethylamine (31.3 µL, 0.18 mmol) was added at 0 °C, and the mixture was stirred at room temperature for 10 minutes. The reaction was cooled by ice cold bath and **S3** (21.3 mg, 0.1 mmol) was added. The mixture was stirred at room temperature overnight, and all of the volatiles were removed by vacuum pump. The crude product was purified by reverse-phase HPLC (5.0–65% MeCN over 40 minutes). Lyophilization of collected fractions gave the product.

^{1}H NMR (500 MHz, DMSO-d_6) δ 8.30 (t, J = 5.8 Hz, 1H), 7.75 (s, 2H), 7.42 (d, J = 8.0 Hz, 2H), 7.23 (m, 3H), 6.43 (s, 1H), 6.36 (s, 1H), 6.08 (d, J = 18.4 Hz, 1H), 4.29 - 4.32 (m, 1H), 4.25 (d, J = 5.8 Hz, 2H), 4.11 - 4.13 (m, 1H), 3.07 - 3.11 (m, 1H), 2.81- 2.84 (m, 1H), 2.57 - 2.60 (m, 1H), 2.14 (t, J = 7.3 Hz, 2H), 1.47 - 1.60 (m, 4H), 1.30 - 1.36 (m, 2H).^{13}C NMR (125 MHz, DMSO-d_6) δ 171.94, 162.59, 145.45, 139.99, 136.09, 127.41, 126.43, 122.62, 60.93, 59.07, 55.33, 41.64, 39.74, 35.04, 28.13, 27.93, 25.20. HRMS calculated for $C_{19}H_{26}BN_3O_4S$ $[M+H]^+$ 404.1771, observed 404.1836.

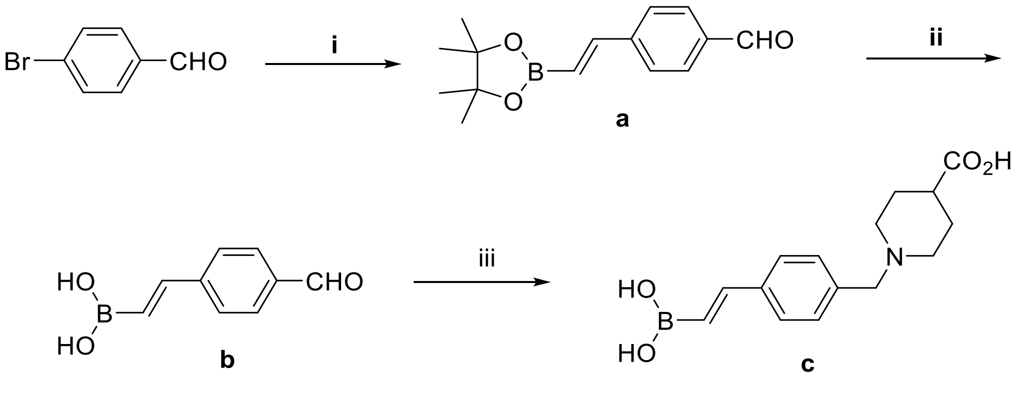

Reagents and conditions: (i) Pd(P^tBu_3)$_4$, Pinacol vinylboronate, TEA, toluene, 90 °C, 16 h; (ii) $NaIO_4$, HCl, THF/H_2O, r.t., 2 h; (iii) piperidine-4-carboxylic acid, NaBH(OAc)$_3$, DCE, 30 °C, overnight.

(*E*)-4-(2-(4,4,5,5-tetramethyl-1,3,2-dioxaborolan-2-yl)vinyl)benzaldehyde (a) To a mixture of 4-bromobenzaldehyde (2 g, 10.9 mmol) and 4,4,5,5-tetramethyl-2-vinyl-1,3,2-dioxaborolane (2.2 mL, 13 mmol, 1.2 equiv.) was added toluene (20 mL), Et_3N (3 mL, 21.8 mmol, 2 equiv.), followed by $Pd(P^tBu_3)_4$ (0.28 g, 1.1 mmol, 10 mol%). The resulting mixture was purged with nitrogen, then heated at 90 °C for 18 hours. After cooling to room temperature, it was quenched with sat. $NaHCO_3$ (20 mL), extracted with EtOAc (30 mL × 3). After evaporation of the solvents, the residue was purified by Biotage column system (EtOAc/Petroleum ether: 0-10%) to afford a white solid. Yield: 60 %.^{1}H NMR (500 MHz, DMSO-d_6) δ 10.01 (s, 1H), 7.90 (d, *J* = 8.2 Hz, 2H), 7.81 (d, *J* = 8.2 Hz, 2H), 7.38 (d, *J* = 18.5 Hz, 1H), 6.36 (d, *J* = 18.5 Hz, 1H), 1.26 (s, 12H). ^{13}C NMR (125 MHz, DMSO-d_6) δ 192.52, 147.68, 142.31, 136.11, 129.78, 127.57, 120.47, 83.19, 24.53. HRMS calculated for $C_{15}H_{19}BO_3$ $[M+H]^+$ 259.1461, observed 259.1489.

(*E*)-(4-formylstyryl)boronic acid (b) (*E*)-4-(2-(4,4,5,5-tetramethyl-1,3,2-dioxaborolan-2-yl)vinyl)benzaldehyde (500 mg, 1.9 mmol) was dissolved in 20 mL mixed solvent of THF:water (4:1). To this solution, $NaIO_4$ (1.2 g, 3.0 equiv.) was added and stirred for 5 minutes. Then an aqueous solution of HCl (2.0 M, 2.0 mL) was added and stirred for 1 hour until the boronic esters were completely consumed as monitored by TLC. The reaction mixture was extracted with EtOAc (20 mL × 3), the combined organic layers were washed with H_2O. After evaporation of the solvents, the residue was purified by Biotage column system (EtOAc/Petroleum ether: 0-20%) to afford a pale-yellow solid. Yield: 88 %.^{1}H NMR (500 MHz, DMSO-d_6) δ 10.00 (s, 1H), 7.93 (s, 2H), 7.90 (d, *J* = 8.2 Hz, 2H), 7.69 (d, *J* = 8.2 Hz, 2H), 7.32 (d, *J* = 18.4 Hz, 1H), 6.32 (d, *J* = 18.4 Hz, 1H). ^{13}C NMR (125 MHz, DMSO-d_6) δ 192.48, 144.31, 143.18, 135.65, 129.91, 127.38, 127.09. HRMS calculated for $C_9H_9BO_3$ $[M+H]^+$ 177.0678, observed 177.0703.

(*E*)-1-(4-(2-boronovinyl)benzyl)piperidine-4-carboxylic acid (c) Piperidine-4-carboxylic acid (100 mg, 0.84 mmol, 1.5 eq) was suspended in dry DCE(15 ml), then (E)-(4-formylstyryl)boronic acid (100 mg, 0.56 mmol) was added. The suspension was stirred for 30 minutes and then sodium triacetoxyborohydride (240 mg, 1.12 mmol, 2 equiv.) was added portionwise and the suspension was stirred overnight at 30 °C. After evaporation of DCE, water (10 ml) was added to quench the reaction and the solution was adjusted to pH 10 by 1 M NaOH. The solution was washed with DCM (10 mL × 3), EtOAc (10 mL × 3) and adjusted to pH 2 by 2 M HCl. The residue was lyophilized and purified by HPLC with Agilent C18 column. Then the residue was lyophilized to afford the title product trifluoroacetate as a white solid. Yield: 42 %.^{1}H NMR (500 MHz, DMSO-d_6) δ 12.56 (s, 1H), 9.44 (s, 1H), 7.85 (s, 2H), 7.57 (d, *J* = 8.2 Hz, 2H), 7.48 (d, *J* = 8.2 Hz, 2H), 7.28 (d, *J* = 18.4 Hz, 1H), 6.19 (d, *J* = 18.4 Hz, 1H), 4.28 (d, *J* = 4.6 Hz, 2H), 3.40 (m, 2H), 3.26-3.28 (m, 1H), 2.91- 2.98 (m, 2H), 2.04 - 2.07 (m, 2H), 1.66-1.74 (m, 2H). ^{13}C NMR (125 MHz, DMSO-d_6) δ 174.45, 158.16, 144.74, 138.74, 131.66, 129.30, 126.82, 124.84, 116.93,58.76, 50.67, 37.74, 25.17. HRMS calculated for $C_{15}H_{20}BNO_4$ $[M+H]^+$ 290.1519, observed 290.1559.

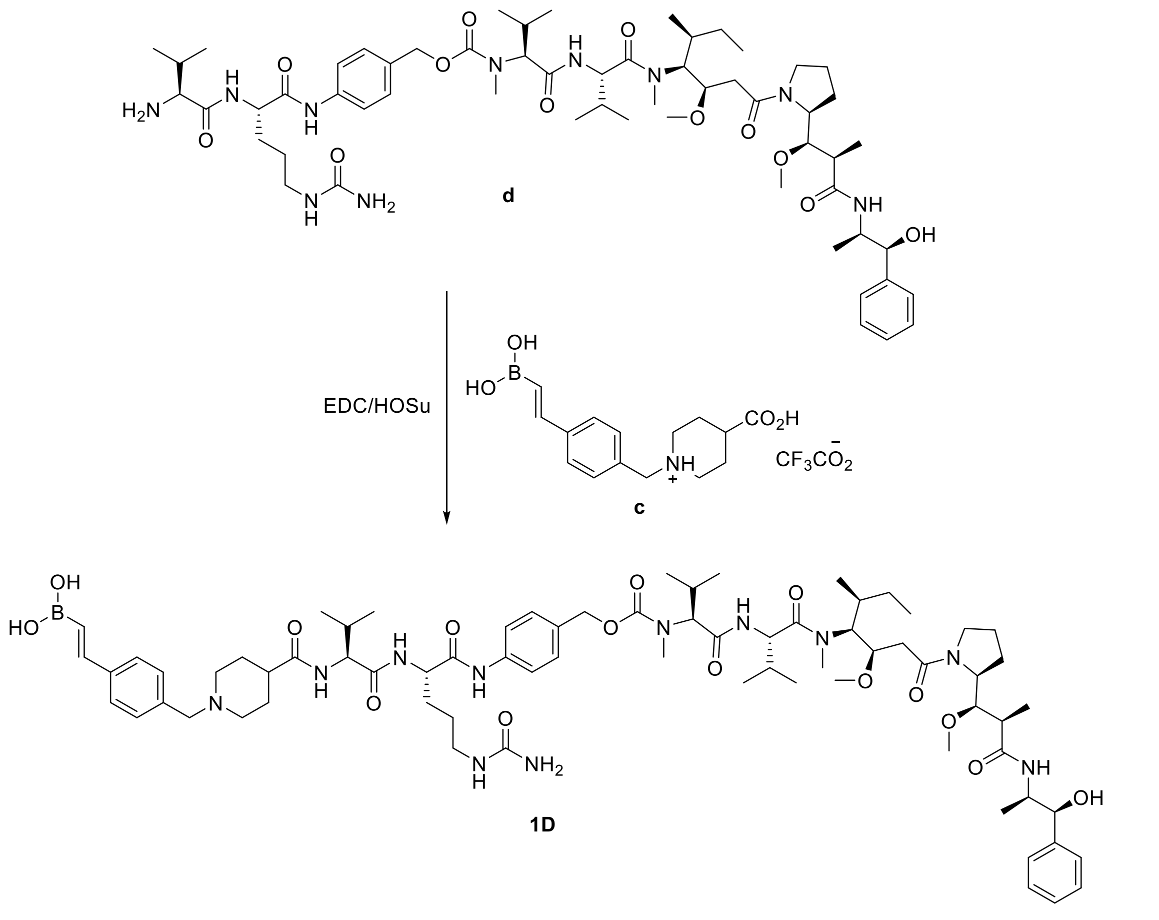

SBA-MMAE (1D). NH_2-Val-Cit-PAB-MMAE (**d**) was synthesized according to the reported literature[4]. Intermediate **c** (20.0 mg, 0.049 mmol, 6 equiv.), HOSu (7.4 mg, 0.064 mmol, 7.8 equiv.), DMAP (3 mg, 0.032 mmol, 3 equiv.) were dissolved in dry DMF (100 μL) and then EDCI (12.3 mg, 0.064 mmol, 7.8 equiv.) was added and the solution was stirred at 30 °C overnight. Then NH_2-Val-Cit-PAB-MMAE (**d**, 10.0 mg, 8 μmol, 1 equiv.) was added to the solution. The reaction was stirred at 30°C overnight and monitored by LC-MS. Finally, the product was purified by HPLC with Agilent C18 column to give a white powder (7.6 mg, 46%). HRMS calculated for $C_{73}H_{112}BN_{11}O_{15}$ $[M+Na]^+$1416.8232, observed 1416.8296.

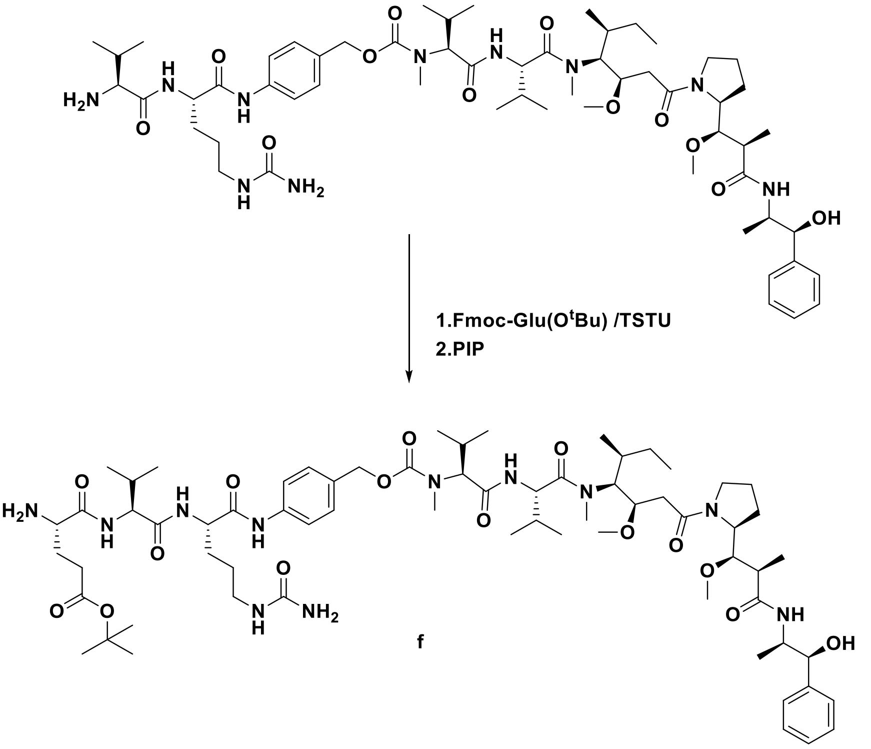

H_2N-Glu(O^{t}Bu)-Val-Cit-PAB-MMAE (f). Fmoc-Glu(O^{t}Bu)-OH (28.0 mg, 0.066 mmol, 1 equiv.), TSTU (23.3 mg, 0.079 mmol, 1.2 equiv.) , DIEA (24 μL, 0.13 mmol, 2 equiv.) were dissolved in dry DMF (460 μL) and the solution was stirred at 30 ℃ for 1 hour. Then the mixture (426 μL) was added to the solution of NH_2-Val-Cit-PAB-MMAE (60 mg, 0.048 mmol, 0.72 equiv.) in 1mL DMF for 3-4 h until HPLC-MS indicated that the reaction was completed. Then 4-Methylpiperidine (9.5 μL, 0.1 mmol, 1.5 equiv.) was added to the mixture and stirred at 30 °C overnight. The product was purified by HPLC with agilent C18 column and lyophilized to get a white powder (56.4 mg, 89%). HRMS Calcd for $[M+H]^+$ 1308.8105, observed 1308.8082.

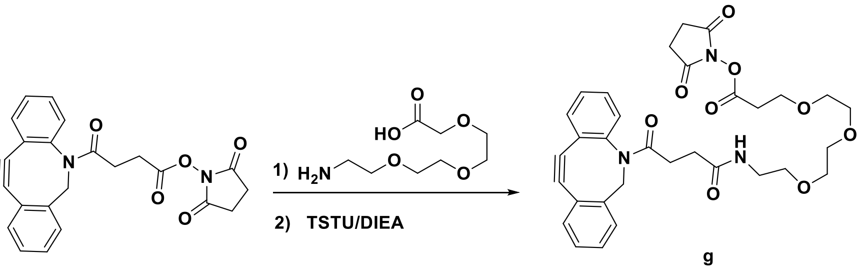

g

DBCO-PEG$_3$-NHS (g). DBCO-PEG$_3$-NHS was synthesized according to the reported literature[5]. DIEA (88 μL, 2 equiv.) was added to a mixture of DBCO-NHS (100 mg, 1 equiv.) and 3-(2-(2-(2-aminoethoxy)edioxy)ethoxy) propanoic acid (66 mg, 1.2 equiv.) in DCM (2 mL), then reaction mixture was stirred overnight at room temperature. After the reaction was completed, the reaction mixture was diluted with DCM. Then the organic layer was washed with brine, 1 M HCl and dried over $MgSO_4$. The residue was concentrated in vacuo to afford a white viscous liquid compound. Then the product (50 mg) was added to a mixture of TSTU (38.5 mg, 1.3 equiv.) and DIEA (26 μL, 1.5 equiv.) in dry DCM / DMF (1.5 mL / 0.5 mL). The reaction mixture was stirred overnight at room temperature. After the reaction was completed, the reaction mixture was diluted with DCM. Then, the organic layer was washed with brine, 1

M HCl and dried over $MgSO_4$. The organic solvent was removed under reduced pressure to afford a viscous liquid compound (52 mg) without further purification.

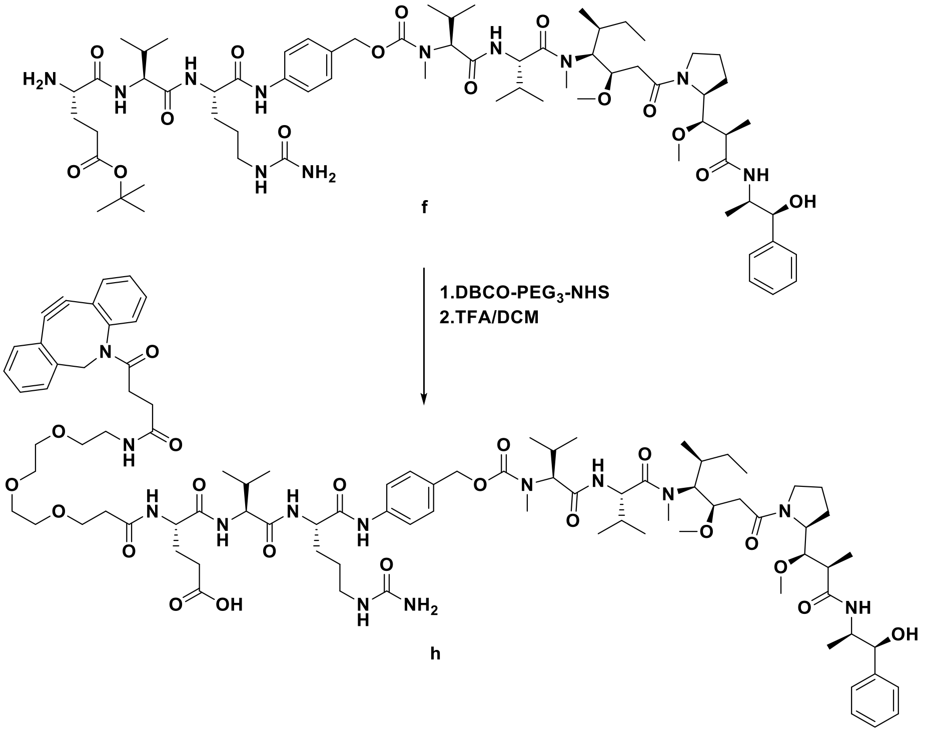

DBCO-MMAE (h). H_2N-Glu(O^{t}Bu)-Val-Cit-PAB-MMAE (30 mg, 0.023 mmol), DBCO-PEG$_3$-NHS (16.3 mg, 0.027 mmol, 1.2 equiv.), DIEA (8 μL, 0.46 mmol, 2 equiv.) were dissolved in dry DMF (600 μL) and the solution was stirred at room temperature for 1 hour until LC-MS indicated that the reaction was completed. Then the solution was concentrated in vacuo and the crude product was precipitated with cold diethyl ether (4 mL) followed by centrifugation at 2,000 × g for 3 minutes (3 times) and dried in vacuo. 30% TFA/DCM (2 mL) was added to the product at 0 ºC for 4-5 hours. Then the reaction mixture was diluted with DCM (4ml), washed with 5 mL water (3 times) and concentrated in vacuo. The resulting crude product was purified by HPLC with agilent C18 column and lyophilized to get a white powder (10 mg, 25%). HRMS Calcd for $[M+H]^+$1742.9582, observed 1742.9537.

5. Preparation of Proteins

a. Expression and purification of recombinant proteins:

Construction of plasmids for recombinant proteins.

The gene of SMT3 was synthesized by Genewiz, Suzhou, China. It was cloned into the vector pET22b. The genes of MBP, Sortase and Trigger factor were cloned into the vector pET28a. The gene of Nanobody was cloned into the vector pET26b. The amino acid sequences are shown below. KOD One^{TM} PCR Master Mix-Blue (ThermoFisher scientific) was used to PCR amplify the DNA. All the gene fragments were assembled by the Gibson assembly kit

(Cat.C115-01, Vazyme).

Protein expression and purification.
E. coli BL21(DE3) cells transformed with His-TEV-SMT3, His-TEV-MBP, His-TEV-Sortase, His-TEV-Trigger factor and His-TEV-Nanobody plasmids were grown in 1 L of LB medium containing kanamycin (50 mg/L) at 37 °C until OD600 = 0.6. Then, expression of His-TEV-MBP, His-TEV-Sortase, His-TEV-Trigger factor and His-TEV-Nanobody were induced by addition of 0.5 mM IPTG overnight at 18 °C. Expression of His-TEV-SMT3 was induced by addition of 0.5 mM IPTG at 37 °C for 4 hours. After harvesting the cells by centrifugation (6,000 rpm for 10 minutes), the cell pellet was lysed by sonication in 25 mL of 50 mM Tris with 150 mM NaCl (pH 7.5) buffer. The suspension was centrifuged at 12,000 rpm for 30 minutes to remove cell debris. The supernatant was loaded onto 2 mL Ni-Charged Resin (Genscript, Cat. NO. L00666-100), first washed with 40 mL of 20 mM Tris with 150 mM NaCl (pH 7.5), and then washed with 40 mL of 20 mM imidazole in 20 mM Tris with 150 mM NaCl (pH 7.5). The protein was eluted from the column with buffer containing 250 mM imidazole, 20 mM Tris, 150 mM NaCl (pH 7.5). Imidazole was removed from protein using Pur-A-Lyzer™ Maxi Dialysis Kit (PURX12005, Sigma-Aldrich), Tris buffer was changed to NMM buffer during the dialysis. The protein was analyzed by LC-MS to confirm its purity and molecular weight. TEV enzyme digestion experiments were carried out by mixing the enzyme and substrate in the ratio of 1:20 at 4°C overnight.

His-TEV-SMT3
MGSHHHHHHENLYFQSDSEVNQEAKPEVKPEVKPETHINLKVSDGSSEIFFKIKKTTPLRRL
MEAFAKRQGKEMDSLRFLYDGIRIQADQTPEDLDMEDNDIIEAHREQIGG

SMT3
SDSEVNQEAKPEVKPEVKPETHINLKVSDGSSEIFFKIKKTTPLRRLMEAFAKRQGKEMDSL
RFLYDGIRIQADQTPEDLDMEDNDIIEAHREQIGG

His-TEV-SMT3 **(C-*CAST)***
MGSHHHHHHENLYFQSDSEVNQEAKPEVKPEVKPETHINLKVSDGSSEIFFKIKKTTPLRRL
MEAFAKRQGKEMDSLRFLYDGIRIQADQTPEDLDMEDNDIIEAHREQIGGFFKKDDHAA

SMT3 **(C-*CAST)***
SDSEVNQEAKPEVKPEVKPETHINLKVSDGSSEIFFKIKKTTPLRRLMEAFAKRQGKEMDSL
RFLYDGIRIQADQTPEDLDMEDNDIIEAHREQIGGFFKKDDHAA

His-TEV-MBP
MHHHHHHHHGSENLYFQGGSKIEEGKLVIWINGDKGYNGLAEVGKKFEKDTGIKVTVEHPDK
LEEKFPQVAATGDGPDIIFWAHDRFGGYAQSGLLAEITPDKAFQDKLYPFTWDAVRYNGKLI
AYPIAVEALSLIYNKDLLPNPPKTWEEIPALDKELKAKGKSALMFNLQEPYFTWPLIAADGG
YAFKYENGKYDIKDVGVDNAGAKAGLTFLVDLIKNKHMNADTDYSIAEAAFNKGETAMTING
PWAWSNIDTSKVNYGVTVLPTFKGQPSKPFVGVLSAGINAASPNKELAKEFLENYLLTDEGL
EAVNKDKPLGAVALKSYEEELAKDPRIAATMENAQKGEIMPNIPQMSAFWYAVRTAVINAAS

GRQTVDEALKDAQT

MBP
GGSKIEEGKLVIWINGDKGYNGLAEVGKKFEKDTGIKVTVEHPDKLEEKFPQVAATGDGPDI
IFWAHDRFGGYAQSGLLAEITPDKAFQDKLYPFTWDAVRYNGKLIAYPIAVEALSLIYNKDL
LPNPPKTWEEIPALDKELKAKGKSALMFNLQEPYFTWPLIAADGGYAFKYENGKYDIKDVGV
DNAGAKAGLTFLVDLIKNKHMNADTDYSIAEAAFNKGETAMTINGPWAWSNIDTSKVNYGVT
VLPTFKGQPSKPFVGVLSAGINAASPNKELAKEFLENYLLTDEGLEAVNKDKPLGAVALKSY
EEELAKDPRIAATMENAQKGEIMPNIPQMSAFWYAVRTAVINAASGRQTVDEALKDAQT

His-TEV-MBP **(C-*CAST*)**
MHHHHHHHHGSENLYFQGGSKIEEGKLVIWINGDKGYNGLAEVGKKFEKDTGIKVTVEHPDK
LEEKFPQVAATGDGPDIIFWAHDRFGGYAQSGLLAEITPDKAFQDKLYPFTWDAVRYNGKLI
AYPIAVEALSLIYNKDLLPNPPKTWEEIPALDKELKAKGKSALMFNLQEPYFTWPLIAADGG
YAFKYENGKYDIKDVGVDNAGAKAGLTFLVDLIKNKHMNADTDYSIAEAAFNKGETAMTING
PWAWSNIDTSKVNYGVTVLPTFKGQPSKPFVGVLSAGINAASPNKELAKEFLENYLLTDEGL
EAVNKDKPLGAVALKSYEEELAKDPRIAATMENAQKGEIMPNIPQMSAFWYAVRTAVINAAS
GRQTVDEALKDAQTFFKKDDHAA

MBP **(C-*CAST*)**
GGSKIEEGKLVIWINGDKGYNGLAEVGKKFEKDTGIKVTVEHPDKLEEKFPQVAATGDGPDI
IFWAHDRFGGYAQSGLLAEITPDKAFQDKLYPFTWDAVRYNGKLIAYPIAVEALSLIYNKDL
LPNPPKTWEEIPALDKELKAKGKSALMFNLQEPYFTWPLIAADGGYAFKYENGKYDIKDVGV
DNAGAKAGLTFLVDLIKNKHMNADTDYSIAEAAFNKGETAMTINGPWAWSNIDTSKVNYGVT
VLPTFKGQPSKPFVGVLSAGINAASPNKELAKEFLENYLLTDEGLEAVNKDKPLGAVALKSY
EEELAKDPRIAATMENAQKGEIMPNIPQMSAFWYAVRTAVINAASGRQTVDEALKDAQTFFK
KDDHAA

MBP **(N-*CAST*)**
GGSFFKKDDHAAGSKIEEGKLVIWINGDKGYNGLAEVGKKFEKDTGIKVTVEHPDKLEEKFP
QVAATGDGPDIIFWAHDRFGGYAQSGLLAEITPDKAFQDKLYPFTWDAVRYNGKLIAYPIAV
EALSLIYNKDLLPNPPKTWEEIPALDKELKAKGKSALMFNLQEPYFTWPLIAADGGYAFKYE
NGKYDIKDVGVDNAGAKAGLTFLVDLIKNKHMNADTDYSIAEAAFNKGETAMTINGPWAWSN
IDTSKVNYGVTVLPTFKGQPSKPFVGVLSAGINAASPNKELAKEFLENYLLTDEGLEAVNKD
KPLGAVALKSYEEELAKDPRIAATMENAQKGEIMPNIPQMSAFWYAVRTAVINAASGRQTVD
EALKDAQT

MBP (G^{177}-***CAST***)
GGSKIEEGKLVIWINGDKGYNGLAEVGKKFEKDTGIKVTVEHPDKLEEKFPQVAATGDGPDI
IFWAHDRFGGYAQSGLLAEITPDKAFQDKLYPFTWDAVRYNGKLIAYPIAVEALSLIYNKDL
LPNPPKTWEEIPALDKELKAKGKSALMFNLQEPYFTWPLIAADGGYAFKYENGGSFFKKDDH
AAGGSKYDIKDVGVDNAGAKAGLTFLVDLIKNKHMNADTDYSIAEAAFNKGETAMTINGPWA
WSNIDTSKVNYGVTVLPTFKGQPSKPFVGVLSAGINAASPNKELAKEFLENYLLTDEGLEAV
NKDKPLGAVALKSYEEELAKDPRIAATMENAQKGEIMPNIPQMSAFWYAVRTAVINAASGRQ

TVDEALKDAQT

MBP-SGSGLPETGG
MHHHHHHHHGSENLYFQGGSKIEEGKLVIWINGDKGYNGLAEVGKKFEKDTGIKVTVEHPDK
LEEKFPQVAATGDGPDIIFWAHDRFGGYAQSGLLAEITPDKAFQDKLYPFTWDAVRYNGKLI
AYPIAVEALSLIYNKDLLPNPPKTWEEIPALDKELKAKGKSALMFNLQEPYFTWPLIAADGG
YAFKYENGKYDIKDVGVDNAGAKAGLTFLVDLIKNKHMNADTDYSIAEAAFNKGETAMTING
PWAWSNIDTSKVNYGVTVLPTFKGQPSKPFVGVLSAGINAASPNKELAKEFLENYLLTDEGL
EAVNKDKPLGAVALKSYEEELAKDPRIAATMENAQKGEIMPNIPQMSAFWYAVRTAVINAAS
GRQTVDEALKDAQTSGSGLPETGG

His-TEV-Nanobady
MHHHHHHHHGSENLYFQGGSDQVQLQESGGGLVQAGGSLRLSCAASGYISDAYYMGWYRQAP
GKEREFVATITHGTNTYYADSVKGRFTISRDNAKNTVYLQMNSLKPEDTAVYYCAVLETRSY
SFRYWGQGTQVTVSSLE

Nanobody
GGSDQVQLQESGGGLVQAGGSLRLSCAASGYISDAYYMGWYRQAPGKEREFVATITHGTNTY
YADSVKGRFTISRDNAKNTVYLQMNSLKPEDTAVYYCAVLETRSYSFRYWGQGTQVTVSSLE

His- TEV-Nanobody **(**C**-*CAST)***
MHHHHHHHHGSENLYFQGGSDQVQLQESGGGLVQAGGSLRLSCAASGYISDAYYMGWYRQAP
GKEREFVATITHGTNTYYADSVKGRFTISRDNAKNTVYLQMNSLKPEDTAVYYCAVLETRSY
SFRYWGQGTQVTVSSLEFFKKDDHAA

Nanobody **(**C**-*CAST)***
GGSDQVQLQESGGGLVQAGGSLRLSCAASGYISDAYYMGWYRQAPGKEREFVATITHGTNTY
YADSVKGRFTISRDNAKNTVYLQMNSLKPEDTAVYYCAVLETRSYSFRYWGQGTQVTVSSLE
FFKKDDHAA

Nanobody **(**N**-*CAST)***
GGSFFKKDDHAAGSDQVQLQESGGGLVQAGGSLRLSCAASGYISDAYYMGWYRQAPGKEREF
VATITHGTNTYYADSVKGRFTISRDNAKNTVYLQMNSLKPEDTAVYYCAVLETRSYSFRYWG
QGTQVTVSSLE

Nanobody (G^{46}-***CAST***)
GGSDQVQLQESGGGLVQAGGSLRLSCAASGYISDAYYMGWYRQAPGSFFKKDDHAAGGSKER
EFVATITHGTNTYYADSVKGRFTISRDNAKNTVYLQMNSLKPEDTAVYYCAVLETRSYSFRY
WGQGTQVTVSSLE

His-TEV-Trigger Factor
MHHHHHHHHGSENLYFQGGSQVSVETTQGLGRRVTITIAADSIETAVKSELVNVAKKVRIDG
FRKGKVPMNIVAQRYGASVRQDVLGDLMSRNFIDAIIKEKINPAGAPTYVPGEYKLGEDFTY
SVEFEVYPEVELQGLEAIEVEKPIVEVTDADVDGMLDTLRKQQATWKEKDGAVEAEDRVTID

FTGSVDGEEFEGGKASDFVLAMGQGRMIPGFEDGIKGHKAGEEFTIDVTFPEEYHAENLKGK
AAKFAINLKKVEERELPELTAEFIKRFGVEDGSVEGLRAEVRKNMERELKSAIRNRVKSQAI
EGLVKANDIDVPAALIDSEIDVLRRQAAQRFGGNEKQALELPRELFEEQAKRRVVVGLLLGE
VIRTNELKADEERVKGLIEEMASAYEDPKEVIEFYSKNKELMDNMRNVALEEQAVEAVLAKA
KVTEKETTFNELMNQQASAG

Trigger Factor
GGSQVSVETTQGLGRRVTITIAADSIETAVKSELVNVAKKVRIDGFRKGKVPMNIVAQRYGA
SVRQDVLGDLMSRNFIDAIIKEKINPAGAPTYVPGEYKLGEDFTYSVEFEVYPEVELQGLEA
IEVEKPIVEVTDADVDGMLDTLRKQQATWKEKDGAVEAEDRVTIDFTGSVDGEEFEGGKASD
FVLAMGQGRMIPGFEDGIKGHKAGEEFTIDVTFPEEYHAENLKGKAAKFAINLKKVEERELP
ELTAEFIKRFGVEDGSVEGLRAEVRKNMERELKSAIRNRVKSQAIEGLVKANDIDVPAALID
SEIDVLRRQAAQRFGGNEKQALELPRELFEEQAKRRVVVGLLLGEVIRTNELKADEERVKGL
IEEMASAYEDPKEVIEFYSKNKELMDNMRNVALEEQAVEAVLAKAKVTEKETTFNELMNQQA
SAG

His-TEV-Trigger Factor **(C-*CAST)***
MHHHHHHHHGSENLYFQGGSQVSVETTQGLGRRVTITIAADSIETAVKSELVNVAKKVRIDG
FRKGKVPMNIVAQRYGASVRQDVLGDLMSRNFIDAIIKEKINPAGAPTYVPGEYKLGEDFTY
SVEFEVYPEVELQGLEAIEVEKPIVEVTDADVDGMLDTLRKQQATWKEKDGAVEAEDRVTID
FTGSVDGEEFEGGKASDFVLAMGQGRMIPGFEDGIKGHKAGEEFTIDVTFPEEYHAENLKGK
AAKFAINLKKVEERELPELTAEFIKRFGVEDGSVEGLRAEVRKNMERELKSAIRNRVKSQAI
EGLVKANDIDVPAALIDSEIDVLRRQAAQRFGGNEKQALELPRELFEEQAKRRVVVGLLLGE
VIRTNELKADEERVKGLIEEMASAYEDPKEVIEFYSKNKELMDNMRNVALEEQAVEAVLAKA
KVTEKETTFNELMNQQASAGFFKKDDHAA

Trigger Factor **(C-*CAST)***
GGSQVSVETTQGLGRRVTITIAADSIETAVKSELVNVAKKVRIDGFRKGKVPMNIVAQRYGA
SVRQDVLGDLMSRNFIDAIIKEKINPAGAPTYVPGEYKLGEDFTYSVEFEVYPEVELQGLEA
IEVEKPIVEVTDADVDGMLDTLRKQQATWKEKDGAVEAEDRVTIDFTGSVDGEEFEGGKASD
FVLAMGQGRMIPGFEDGIKGHKAGEEFTIDVTFPEEYHAENLKGKAAKFAINLKKVEERELP
ELTAEFIKRFGVEDGSVEGLRAEVRKNMERELKSAIRNRVKSQAIEGLVKANDIDVPAALID
SEIDVLRRQAAQRFGGNEKQALELPRELFEEQAKRRVVVGLLLGEVIRTNELKADEERVKGL
IEEMASAYEDPKEVIEFYSKNKELMDNMRNVALEEQAVEAVLAKAKVTEKETTFNELMNQQA
SAGFFKKDDHAA

His-TEV-Sortase
MHHHHHHHHGSENLYFQGGSQAKPQIPKDKSKVAGYIEIPDADIKEPVYPGPATREQLNRGV
SFAEENESLDDQNISIAGHTFIDRPNYQFTNLKAAKKGSMVYFKVGNETRKYKMTSIRNVKP
TAVEVLDEQKGKDKQLTLITCDDYNEETGVWETRKIFVATEVKLE

Sortase
GGSQAKPQIPKDKSKVAGYIEIPDADIKEPVYPGPATREQLNRGVSFAEENESLDDQNISIA
GHTFIDRPNYQFTNLKAAKKGSMVYFKVGNETRKYKMTSIRNVKPTAVEVLDEQKGKDKQLT

LITCDDYNEETGVWETRKIFVATEVKLE

His-TEV-Sortase **(**C**-*CAST)***
MHHHHHHHHGSENLYFQGGSQAKPQIPKDKSKVAGYIEIPDADIKEPVYPGPATREQLNRGV
SFAEENESLDDQNISIAGHTFIDRPNYQFTNLKAAKKGSMVYFKVGNETRKYKMTSIRNVKP
TAVEVLDEQKGKDKQLTLITCDDYNEETGVWETRKIFVATEVKLEFFKKDDHAA

Sortase **(**C**-*CAST)***
GGSQAKPQIPKDKSKVAGYIEIPDADIKEPVYPGPATREQLNRGVSFAEENESLDDQNISIA
GHTFIDRPNYQFTNLKAAKKGSMVYFKVGNETRKYKMTSIRNVKPTAVEVLDEQKGKDKQLT
LITCDDYNEETGVWETRKIFVATEVKLEFFKKDDHAA

π-clamp sortase
GASMTGFCPFGQQMGRDPNSQAKPQIPKDKSKVAGYIEIPDADIKEPVYPGPATSEQLNRGV
SFAEENESLDDQNISIAGHTFIDRPNYQFTNLKAAKKGSMVYFKVGNETRKYKMTSIRNVKP
TDVEVLDEQKGKDKQLTLITCDDYNEKTGVWETRKIFVATEVKLEHHHHHH

b. Expression and purification of antibodies

The pVITRO1-trastuzumab plasmid was purchased from Addgene (#61883). The pVITRO1-**Tra-*CAST*** plasmids were constructed by inserting the ***CAST*** peptide at the *C*-terminus of the trastuzumab heavy chain, using the ClonExpress Ultra One Step Cloning Kit which was purchased from Vazyme (C115-01). The light chain and heavy chain sequences for the trastuzumab, **Tra-*CAST***, **Tra-*CASTi***, π-clamp-trastuzumab are listed below:

Trastuzumab-Light Chain
MLPSQLIGFLLLWVPASRGDIQMTQSPSSLSASVGDRVTITCRASQDVNTAVAWYQQKPGKA
PKLLIYSASFLYSGVPSRFSGSRSGTDFTLTISSLQPEDFATYYCQQHYTTPPTFGQGTKLE
IKRTVAAPSVFIFPPSDEQLKSGTASVVCLLNNFYPREAKVQWKVDNALQSGNSQESVTEQD
SKDSTYSLSSTLTLSKADYEKHKVYACEVTHQGLSSPVTKSFNRGEC*

Trastuzumab-Heavy Chain
MDWTWRILFLVAAATGAHSEVQLVESGGGLVQPGGSLRLSCAASGFNIKDTYIHWVRQAPGK
GLEWVARIYPTNGYTRYADSVKGRFTISADTSKNTAYLQMNSLRAEDTAVYYCSRWGGDGFY
AMDYWGQGTLVTVSSASTKGPSVFPLAPSSKSTSGGTAALGCLVKDYFPEPVTVSWNSGALT
SGVHTFPAVLQSSGLYSLSSVVTVPSSSLGTQTYICNVNHKPSNTKVDKKVEPKSCDKTHTC
PPCPAPELLGGPSVFLFPPKPKDTLMISRTPEVTCVVVDVSHEDPEVKFNWYVDGVEVHNAK
TKPREEQYNSTYRVVSVLTVLHQDWLNGKEYKCKVSNKALPAPIEKTISKAKGQPREPQVYT
LPPSRDELTKNQVSLTCLVKGFYPSDIAVEWESNGQPENNYKTTPPVLDSDGSFFLYSKLTV
DKSRWQQGNVFSCSVMHEALHNHYTQKSLSLSPG*

Tra-*CAST*-*Light Chain*
MLPSQLIGFLLLWVPASRGDIQMTQSPSSLSASVGDRVTITCRASQDVNTAVAWYQQKPGKA
PKLLIYSASFLYSGVPSRFSGSRSGTDFTLTISSLQPEDFATYYCQQHYTTPPTFGQGTKLE

IKRTVAAPSVFIFPPSDEQLKSGTASVVCLLNNFYPREAKVQWKVDNALQSGNSQESVTEQD
SKDSTYSLSSTLTLSKADYEKHKVYACEVTHQGLSSPVTKSFNRGEC*

Tra-*CAST*-*Heavy Chain*
MDWTWRILFLVAAATGAHSEVQLVESGGGLVQPGGSLRLSCAASGFNIKDTYIHWVRQAPGK
GLEWVARIYPTNGYTRYADSVKGRFTISADTSKNTAYLQMNSLRAEDTAVYYCSRWGGDGFY
AMDYWGQGTLVTVSSASTKGPSVFPLAPSSKSTSGGTAALGCLVKDYFPEPVTVSWNSGALT
SGVHTFPAVLQSSGLYSLSSVVTVPSSSLGTQTYICNVNHKPSNTKVDKKVEPKSCDKTHTC
PPCPAPELLGGPSVFLFPPKPKDTLMISRTPEVTCVVVDVSHEDPEVKFNWYVDGVEVHNAK
TKPREEQYNSTYRVVSVLTVLHQDWLNGKEYKCKVSNKALPAPIEKTISKAKGQPREPQVYT
LPPSRDELTKNQVSLTCLVKGFYPSDIAVEWESNGQPENNYKTTPPVLDSDGSFFLYSKLTV
DKSRWQQGNVFSCSVMHEALHNHYTQKSLSLSPGFFKKDDHAA*

Tra- *CASTi*-*Light Chain*
MLPSQLIGFLLLWVPASRGDIQMTQSPSSLSASVGDRVTITCRASQDVNTAVAWYQQKPGKA
PKLLIYSASFLYSGVPSRFSGSRSGTDFTLTISSLQPEDFATYYCQQHYTTPPTFGQGTKLE
IKRTVAAPSVFIFPPSDEQLKSGTASVVCLLNNFYPREAKVQWKVDNALQSGNSQESVTEQD
SKDSTYSLSSTLTLSKADYEKHKVYACEVTHQGLSSPVTKSFNRGEC*

Tra-*CASTi*-*Heavy Chain*
MDWTWRILFLVAAATGAHSEVQLVESGGGLVQPGGSLRLSCAASGFNIKDTYIHWVRQAPGK
GLEWVARIYPTNGYTRYADSVKGRFTISADTSKNTAYLQMNSLRAEDTAVYYCSRWGGDGFY
AMDYWGQGTLVTVSSASTKGPSVFPLAPSSKSTSGGTAALGCLVKDYFPEPVTVSWNSGALT
SGVHTFPAVLQSSGLYSLSSVVTVPSSSLGTQTYICNVNHKPSNTKVDKKVEPKSCDKTHTC
PPCPAPELLGGPSVFLFPPKPKDTLMISRTPEVTCVVVDVSHEDPEVKFNWYVDGVEVHNAK
TKPREEQYNSTYRVVSVLTVLHQDWLNGKEYKCKVSNKALPAPIEKTISKAKGQPREPQVYT
LPPSRDELTKNQVSLTCLVKGFYPSDIAVEWESNGQPENNYKTTPPVLDSDGSFFLYSKLTV
DKSRWQQGNVFSCSVMHEALHNHYTQKSLSLSPGIAPDDHAA*

π-clamp-trastuzumab-Light chain
MLPSQLIGFLLLWVPASRGDIQMTQSPSSLSASVGDRVTITCRASQDVNTAVAWYQQKPGKA
PKLLIYSASFLYSGVPSRFSGSRSGTDFTLTISSLQPEDFATYYCQQHYTTPPTFGQGTKLE
IKRTVAAPSVFIFPPSDEQLKSGTASVVCLLNNFYPREAKVQWKVDNALQSGNSQESVTEQD
SKDSTYSLSSTLTLSKADYEKHKVYACEVTHQGLSSPVTKSFNRGEC

π-clamp-trastuzumab-Heavy chain
MDWTWRILFLVAAATGAHSEVQLVESGGGLVQPGGSLRLSCAASGFNIKDTYIHWVRQAPGK
GLEWVARIYPTNGYTRYADSVKGRFTISADTSKNTAYLQMNSLRAEDTAVYYCSRWGGDGFY
AMDYWGQGTLVTVSSASTKGPSVFPLAPSSKSTSGGTAALGCLVKDYFPEPVTVSWNSGALT
SGVHTFPAVLQSSGLYSLSSVVTVPSSSLGTQTYICNVNHKPSNTKVDKKVEPKSCDKTHTC
PPCPAPELLGGPSVFLFPPKPKDTLMISRTPEVTCVVVDVSHEDPEVKFNWYVDGVEVHNAK
TKPREEQYNSTYRVVSVLTVLHQDWLNGKEYKCKVSNKALPAPIEKTISKAKGQPREPQVYT
LPPSRDELTKNQVSLTCLVKGFYPSDIAVEWESNGQPENNYKTTPPVLDSDGSFFLYSKLTV
DKSRWQQGNVFSCSVMHEALHNHYTQKSLSLSPGKGFCPF*

HEK 293F cells (Invitrogen) were cultured in Freestyle medium (Gibco, Lot.2164683) at 37 °C under 5% CO_2 in a CRYSTAL shaker (140 rpm). The cells were transiently transfected with trastuzumab plasmids and polyethylenimine (PEI) (Polysciences, Cat.24765-1) when the cell density reached approximately 1.5×10^6/mL. 1 mg of plasmid was premixed with 2.6 mg PEI in 50 mL of fresh medium for 15 minutes before adding to one liter of cell culture. The transfected cells were cultured for 96 hours before harvesting.

The supernatant of medium was harvested by centrifugation at 1000×g for 5 minutes. Then the supernatants were loaded on Protein A beads (GenScript, Cat. L00210-50) and washed with PBS buffer. Proteins were then eluted with 0.1 M glycine (pH 3.0), then neutralized in 0.2M $NaHCO_3$ (pH 8.0). The eluted proteins were concentrated and subjected to size-exclusion chromatography (Superdex 200 Increase 10/300 GL, GE Healthcare) in NMM buffer (50 mM, 0.2 M NaCl, pH 7.4). The peak fractions were collected and concentrated. The purified IgGs were analyzed by LC-MS to confirm their molecular weight and purity, and stored at -20 °C.

6. Reactions on proteins

SMT3

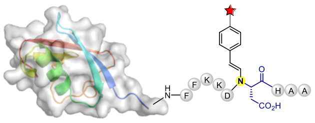

1

To a solution of SMT3 **(C-*CAST*)** (10 μM final concentration) in NMM buffer (50 mM, 0.2 M NaCl, pH 7.4), boronic acid reagent **1** (1 μL of 5 mM stock solution in DMF, 0.1 mM final concentration) and $CuCl_2\cdot2H_2O$ (1.7 μL of 1.5 mM stock solution in water, 50 μM final concentration) were added subsequently. The total reaction volume is 50 μL and the mixture was incubated at 37 °C for 10 minutes

1A, 1B, 1C:

To a solution of SMT3 **(C-*CAST*)**(10 μM final concentration) in NMM buffer (50 mM, 0.2 M NaCl, pH 7.4), boronic acid reagent **1A, 1B or 1C** (1 μL of 25 mM stock solution in DMF, 0.5 mM final concentration) and $CuCl_2\cdot2H_2O$ (1.7 μL of 1.5 mM stock solution in water, 50 μM final concentration) were added subsequently. The total reaction volume is 50 μL. The mixture was incubated at 37 °C for 30 minutes. After reaction is completed, Na_2-EDTA(2 μL of 500 mM stock solution in H_2O, 20 mM final concentration) was added, the crude reaction mixture was directly injected onto LC-MS for analysis.

Nanobody

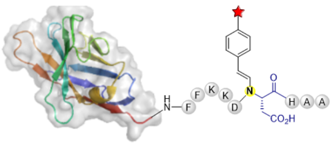

1.

To a solution of nanobody **(C-*CAST*)** (10 µM final concentration) in NMM buffer (50 mM, 0.2 M NaCl, pH 7.4), boronic acid reagent **1** (1 µL of 5 mM stock solution in DMF, 0.1 mM final concentration) and $CuCl_2 \cdot 2H_2O$ (1.7 µL of 1.5 mM stock solution in water, 50 µM final concentration) were added subsequently. The total reaction volume is 50 µL. The mixture was incubated at 37 °C for 1.5 hours

1A,1B,1C :

To a solution of nanobody **(C-*CAST*)** (10 µM final concentration) in NMM buffer (50 mM, 0.2 M NaCl, pH 7.4), boronic acid reagent **1A, 1B or 1C** (1 µL of 25 mM stock solution in DMF, 0.5 mM final concentration) and $CuCl_2 \cdot 2H_2O$ (1.7 µL of 1.5 mM stock solution in water, 50 µM final concentration) were added subsequently. The total reaction volume is 50 µL. The mixture was incubated at 37 °C for 1.5 hours. After reaction is completed, Na_2-EDTA (2 µL of 500 mM stock solution in H_2O, 20 mM final concentration) was added, the crude reaction mixture was directly injected onto LC-MS for analysis.

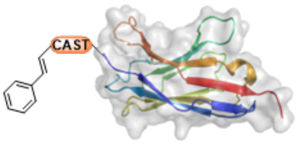

To a solution of nanobody **(N-*CAST*)** (10 µM final concentration) in NMM buffer (50 mM, 0.2 M NaCl, pH 7.4), boronic acid reagent **1** (1 µL of 5 mM stock solution in DMF, 0.1 mM final concentration) and $CuCl_2 \cdot 2H_2O$ (3.4 µL of 1.5 mM stock solution in water, 100 µM final concentration) were added subsequently. The total reaction volume is 50 µL. The mixture was incubated at 37 °C for 1.5 hours

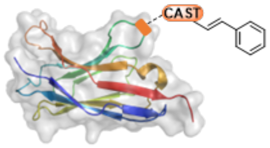

To a solution of nanobody **(G^{46}-*CAST*)** (10 µM final concentration) in NMM buffer (50 mM, 0.2 M NaCl, pH 7.4), boronic acid reagent **1** (2 µL of 5 mM stock solution in DMF, 0.2 mM final concentration) and $CuCl_2 \cdot 2H_2O$ (3.4 µL of 1.5 mM stock solution in water, 100 µM final concentration) were added subsequently. The total reaction volume is 50 µL. The mixture was incubated at 37 °C for 1.5 hours

MBP

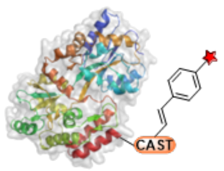

1.

To a solution of MBP **(C-*CAST)*** (10 μM final concentration) in NMM buffer (50 mM, 0.2 M NaCl, pH 7.4), boronic acid reagent **1** (1 μL of 5 mM stock solution in DMF, 0.1 mM final concentration) and $CuCl_2\cdot 2H_2O$ (1.7 μL of 1.5 mM stock solution in water, 50 μM final concentration) were added subsequently. The total reaction volume is 50 μL. The mixture was incubated at 37 °C for 1 hour.

1A, 1B, 1C:

To a solution of MBP **(C-*CAST)*** (10 μM final concentration) in NMM buffer (50 mM, 0.2 M NaCl, pH 7.4), boronic acid reagent **1A, 1B or 1C** (1 μL of 25 mM stock solution in DMF, 0.5 mM final concentration) and $CuCl_2\cdot 2H_2O$ (1.7 μL of 1.5 mM stock solution in water, 50 μM final concentration) were added subsequently. The total reaction volume is 50 μL, the mixture was incubated at 37 °C for 2 hours. After reaction is completed, Na_2-EDTA(2 μL of 500 mM stock solution in H_2O, 20 mM final concentration) was added, The crude reaction mixture was directly injected onto LC-MS for analysis.

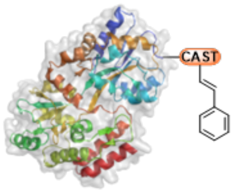

To a solution of MBP **(N-*CAST)*** (10 μM final concentration) in NMM buffer (50 mM, 0.2 M NaCl, pH 7.4), boronic acid reagent **1** (1 μL of 5 mM stock solution in DMF, 0.1 mM final concentration) and $CuCl_2\cdot 2H_2O$ (1.7 μL of 1.5 mM stock solution in water, 50 μM final concentration) were added subsequently. The total reaction volume is 50 μL. The mixture was incubated at 37 °C for 1 hour.

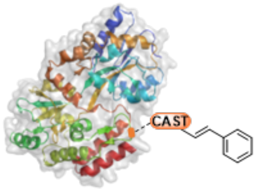

To a solution of MBP **(G^{177}-*CAST)*** (10 μM final concentration) in NMM buffer (50 mM, 0.2 M NaCl, pH 7.4), boronic acid reagent **1** (1 μL of 5 mM stock solution in DMF, 0.1 mM final concentration) and $CuCl_2\cdot 2H_2O$ (1.7 μL of 1.5 mM stock solution in water, 50 μM final concentration) were added subsequently. The total reaction volume is 50 μL. The mixture was incubated at 37 °C for 1 hour.

Trigger factor

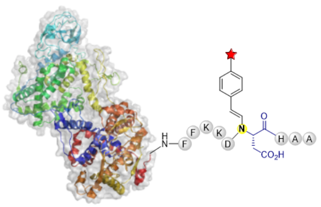

1.

To a solution of trigger factor **(C-*CAST*)** (5 μM final concentration) in NMM buffer (50 mM, 0.2 M NaCl, pH 7.4), boronic acid reagent **1** (1 μL of 5 mM stock solution in DMF, 0.1 mM final concentration) and $CuCl_2 \cdot 2H_2O$ (1.7 μL of 1.5 mM stock solution in water, 50 μM final concentration) were added subsequently. The total reaction volume is 50 μL. The mixture was incubated at 37 °C for 2 hours.

1A, 1B, 1C:

To a solution of trigger factor **(C-*CAST*)** (5 μM final concentration) in NMM buffer (50 mM, 0.2 M NaCl, pH 7.4), boronic acid reagent **1A, 1B or 1C** (1 μL of 25 mM stock solution in DMF, 0.5 mM final concentration) and $CuCl_2 \cdot 2H_2O$ (1.7 μL of 1.5 mM stock solution in water, 50 μM final concentration) were added subsequently, the total reaction volume is 50 μL. The mixture was incubated at 37 °C for 2 hours.

After reaction is completed, Na_2-EDTA (2 μL of 500 mM stock solution in H_2O, 20 mM final concentration) was added, the crude reaction mixture was directly injected onto LC-MS for analysis.

Sortase

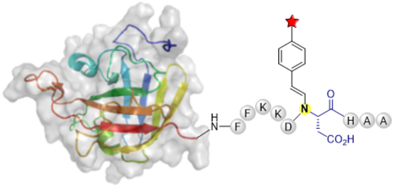

Sortase **(C-*CAST*)** (10 μM) was incubated with Maleimide (5 mM) in PBS at r.t. for 40 min, Then PBS buffer was exchanged into NMM buffer (50 mM, pH 7.4, 0.2 M NaCl).

1.

To a solution of maleimide modified Sortase **(C-*CAST*)** (10 μM final concentration) in NMM buffer (50 mM, 0.2 M NaCl, pH 7.4), boronic acid reagent **1** (1 μL of 5 mM stock solution in DMF, 0.1 mM final concentration) and $CuCl_2 \cdot 2H_2O$ (1.7 μL of 1.5 mM stock solution in water, 50 μM final concentration) were added subsequently The total reaction volume is 50 μL. The mixture was incubated at 37 °C for 30 minutes.

1A, 1B, 1C:

To a solution of maleimide modified Sortase **(C-*CAST*)** (10 μM final concentration) in NMM buffer (50 mM, 0.2 M NaCl, pH 7.4), boronic acid reagent **1A, 1B or 1C** (1 μL of 25 mM stock solution in DMF, 0.5 mM final concentration) and $CuCl_2 \cdot 2H_2O$ (1.7 μL of 1.5 mM stock solution in water, 50 μM final concentration) were added subsequently The total reaction volume is 50 μL. The mixture was incubated at 37 °C for 30 minutes.

After reaction is completed, Na_2-EDTA (2 μL of 500 mM stock solution in H_2O, 20 mM final concentration) was added, the crude reaction mixture was directly injected onto LC-MS for analysis.

Tra-*CAST*

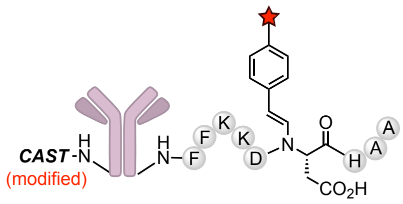

1.

To a solution of **Tra-*CAST*** (4 µM final concentration) in NMM buffer (50 mM, 0.2 M NaCl, pH 7.4), boronic acid reagent **1** (1 µL of 12.5 mM stock solution in DMF, 0.25 mM final concentration) and $CuCl_2·2H_2O$ (1 µL of 1.0 mM stock solution in water, 20 µM final concentration) were added subsequently. The total reaction volume is 50 µL. The mixture was incubated at 37 °C for 2 hours.

1A, 1B, 1C:

To a solution of **Tra-*CAST*** (4 µM final concentration) in NMM buffer (50 mM, 0.2 M NaCl, pH 7.4), boronic acid reagent **1A, 1B or 1C** (1 µL of 12.5 mM stock solution in DMF, 0.25 mM final concentration) and $CuCl_2·2H_2O$ (1 µL of 1.0 mM stock solution in water, 20 µM final concentration) were added subsequently, the total reaction volume is 50 µL. The mixture was incubated at 37 °C for 4 hours.

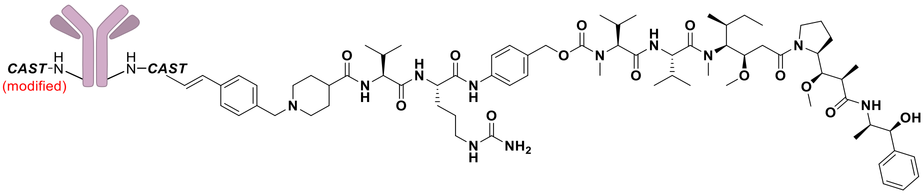

SBA-MMAE

To a solution of **Tra-*CAST*** (4 µM final concentration) in NMM buffer (50 mM, 0.2 M NaCl, pH 7.4), **SBA-MMAE** (1 µL of 25 mM stock solution in DMF, 0.5 mM final concentration) and $CuCl_2·2H_2O$ (1.5 µL of 1.0 mM stock solution in water, 30 µM final concentration), 5 µL DMF were added subsequently. The total reaction volume is 50 µL. The mixture was incubated at 37 °C for 7 hours. After reaction is completed, Na_2-EDTA (2 µL of 500 mM stock solution in H_2O, 20 mM final concentration) was added the crude reaction mixture was directly injected onto LC-MS for analysis.

Tra-*CASTi*

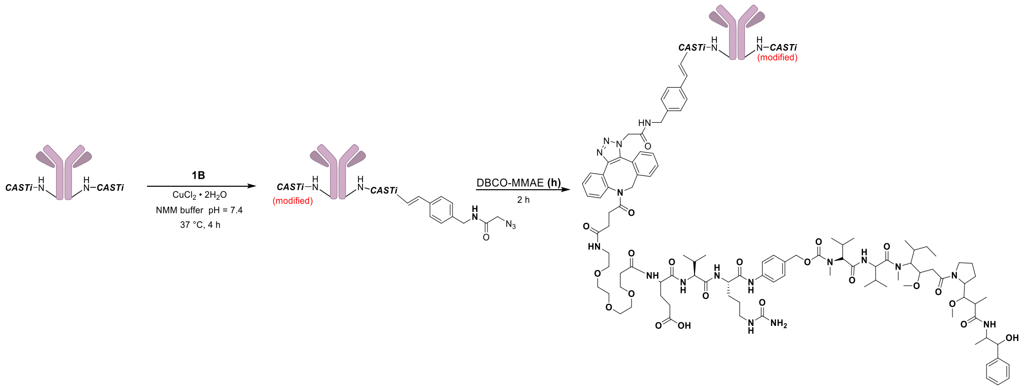

1B.

To a solution of **Tra-*CASTi*** (4 µM final concentration) in NMM buffer (50 mM, 0.2 M NaCl, pH 7.4), boronic acid reagent **1B** (20 µL of 12.5 mM stock solution in DMF, 0.25 mM final concentration) and $CuCl_2 \cdot 2H_2O$ (20 µL of 1.0 mM stock solution in water, 20 µM final concentration) were subsequently added. The total reaction volume is 1 mL. The mixture was incubated at 37 °C for 4 hours. After the reaction was completed, Na_2-EDTA (40 µL of 500 mM stock solution in H_2O, 20 mM final concentration) was added. Excessive **1B** is removed during dialysis

DBCO- MMAE (h)

Click reactions for MMAE installation: DBCO-MMAE (**h**) (4 µL of 20 mM stock solution in DMSO, 20 equivalents) was added to a solution of the **1B** modified **Tra-*CASTi*** (4 µM, total reaction volume is 1 mL) conjugate in PBS, and the mixture was incubated at 37 °C for 2 hours. The reaction was monitored using LC-MS. After the reaction was completed, excess **DBCO-MMAE** was removed during dialysis.

7. Protein raw data

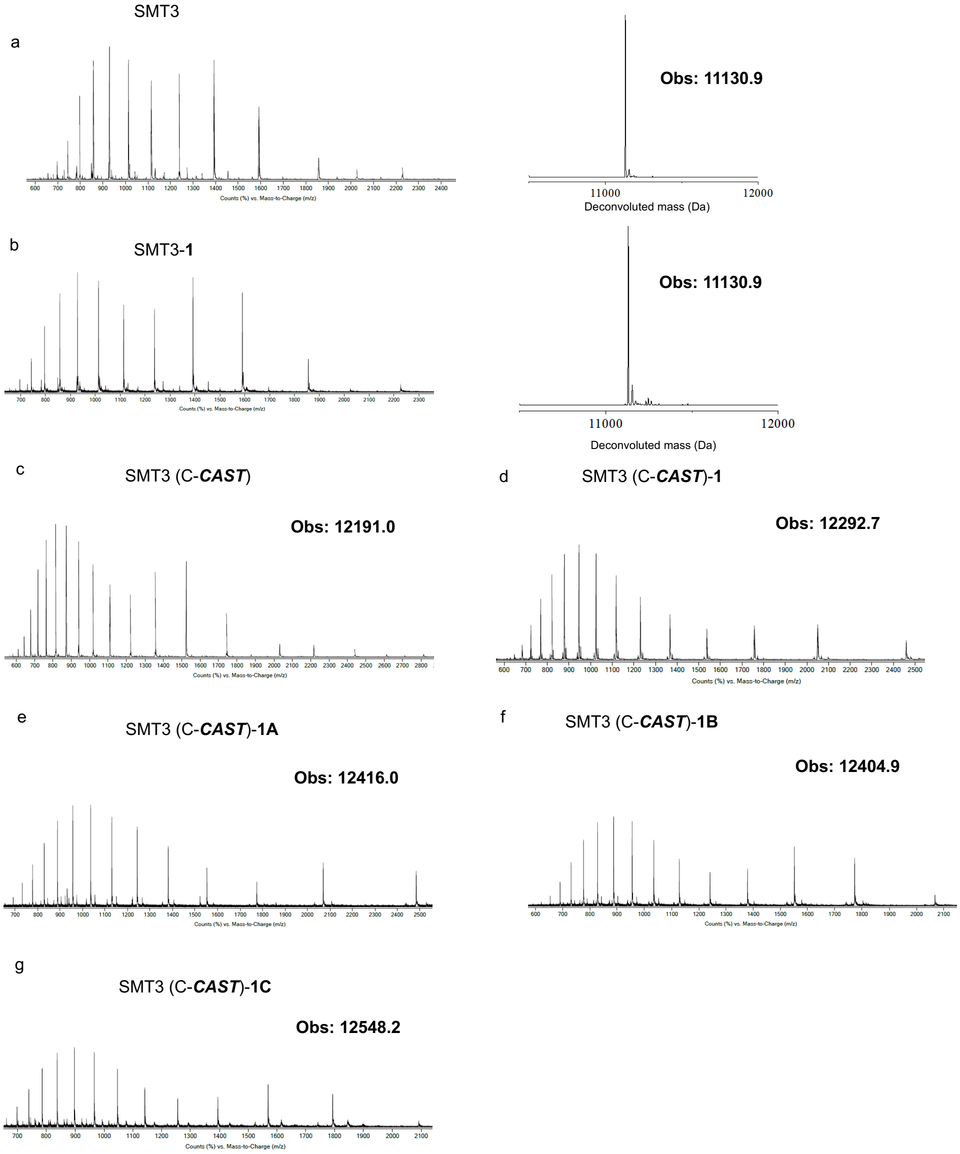

Supplementary Figure 25. **ESI ion series/deconvolution MS spectra: reactions between SMT3 and 1, 1A, 1B, 1C**. **a**. SMT3 protein MS analysis. **b**. Reaction of SMT3 with **1** (No modification observed). Conditions: SMT3 (10 μM), $CuCl_2 \cdot 2H_2O$ (30 μM), **1** (100 μM) in NMM buffer (50 mM pH 7.4, 0.2 M NaCl), 37 °C, 10 minutes. **c**. SMT3 **(C-*CAST*)**. **d**. Reaction of SMT3 **(C-*CAST*)** with **1**. Conditions: SMT3 **(C-*CAST*)** (10 μM), $CuCl_2 \cdot 2H_2O$ (50 μM), **1** (100 μM) in NMM buffer (50 mM pH 7.4, 0.2 M NaCl), 37 °C, 10 minutes. **e**. Reaction of SMT3 **(C-*CAST*)** with **1A**. **f**. Reaction of SMT3 **(C-*CAST*)** with **1B. g**. Reaction of SMT3 **(C-*CAST*)** with **1C**. Conditions: SMT3 **(C-*CAST*)** (10 μM), $CuCl_2 \cdot 2H_2O$ (50 μM), **1A, 1B** or **1C** (500 μM) in NMM buffer (50 mM, pH 7.4, 0.2 M NaCl), 37 °C, 30 minutes.

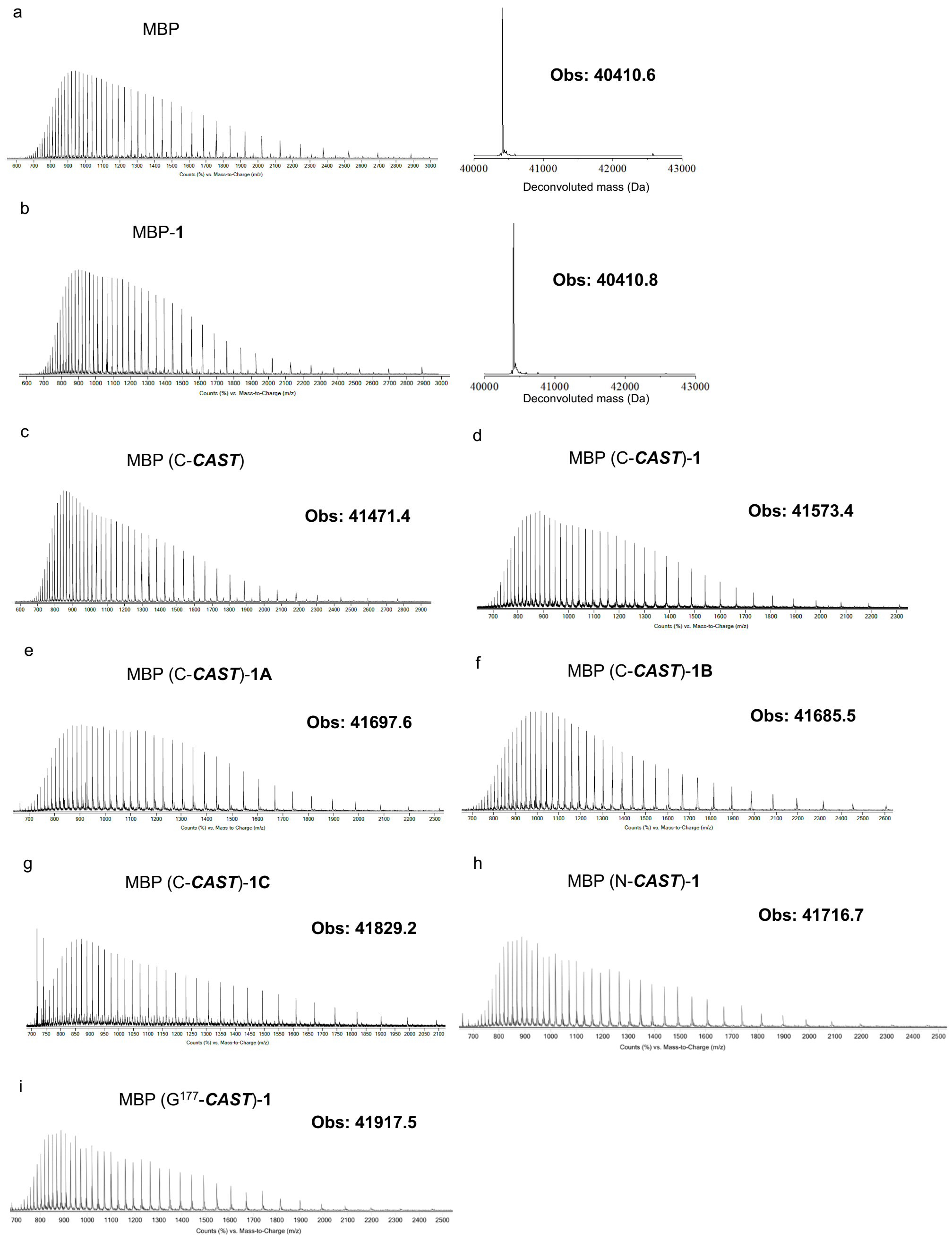

Supplementary Figure 26. **ESI ion series/deconvolution MS spectra: reactions between MBP and 1, 1A, 1B, 1C**. **a**. MBP protein MS analysis. **b**. Reaction of MBP with **1** (no modification observed). Conditions: MBP (10 µM), $CuCl_2\cdot 2H_2O$ (50 µM), **1** (100 µM) in NMM buffer (50 mM pH 7.4, 0.2 M NaCl), 37 °C, 1 hour. **c**. MBP **(C-*CAST*)** protein MS analysis. **d**. Reaction of MBP **(C-*CAST*)** with **1**. Conditions: MBP **(C-*CAST*)** (10 µM), $CuCl_2\cdot 2H_2O$ (50 µM), **1** (100 µM) in NMM buffer (50 mM pH 7.4, 0.2 M NaCl), 37 °C, 1 hour. **e**. Reaction of MBP **(C-*CAST*)** with **1A**. **f**. Reaction of MBP **(C-*CAST*)** with **1B. g**. Reaction of MBP **(C-*CAST*)** with **1C**. Conditions: MBP **(C-*CAST*)** (10 µM), $CuCl_2\cdot 2H_2O$ (50 µM), **1A, 1B** or **1C** (500 µM) in NMM buffer (50 mM, pH 7.4, 0.2 M NaCl), 37 °C, 2 hours. **h.** Reaction of MBP **(N-*CAST*)** with **1**. **i.** Reaction of MBP **(G^{177}-*CAST*)** with **1.** Conditions: MBP **(N-*CAST*)** or MBP **(G^{177}-*CAST*)** (10 µM), $CuCl_2\cdot 2H_2O$ (50 µM),**1** (100 µM) in NMM buffer (50 mM, pH 7.4, 0.2 M NaCl), 37 °C, 1 hour.

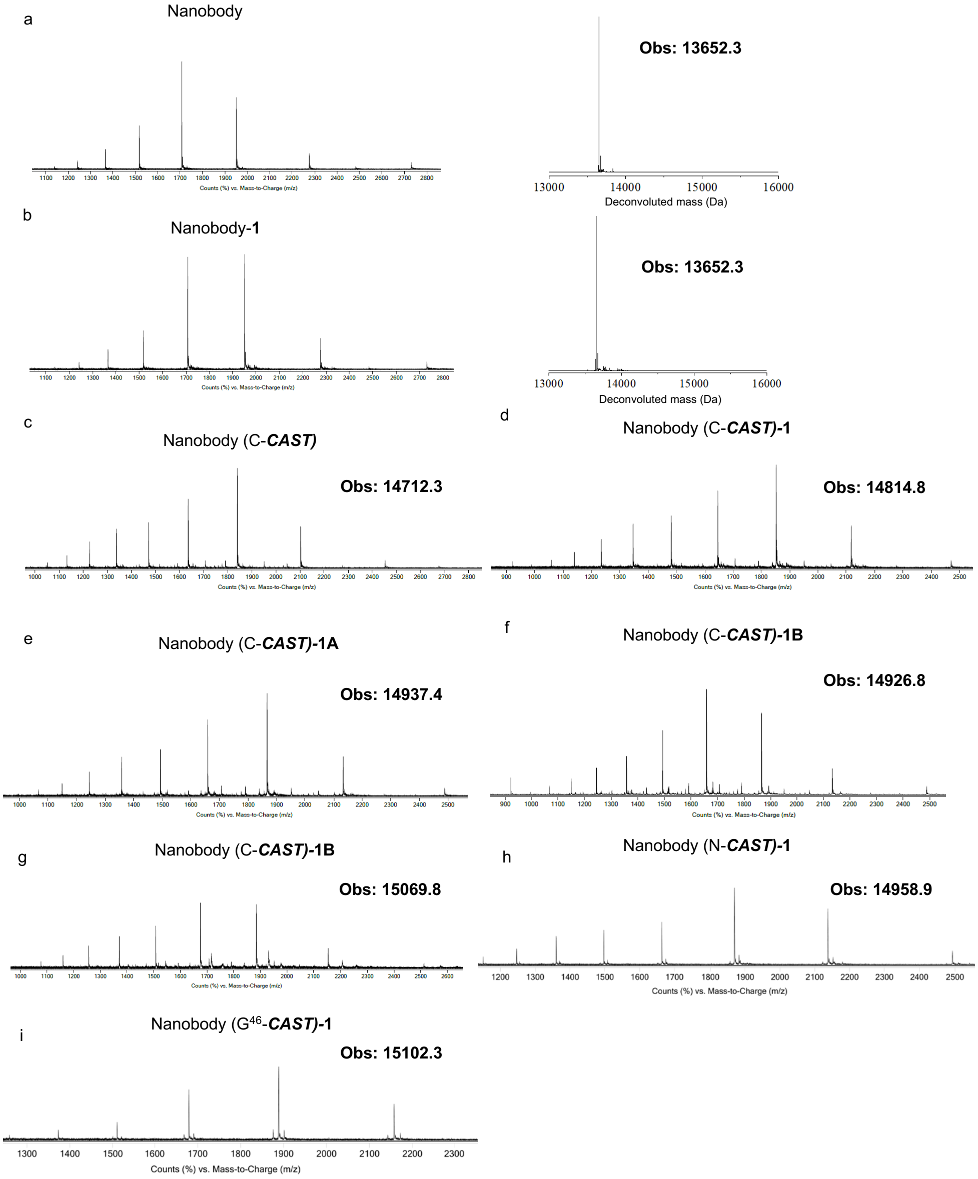

Supplementary Figure 27. ESI ion series/deconvolution MS spectra: reactions between nanobody and 1, 1A, 1B, 1C. **a**. nanobody protein MS analysis. **b**. Reaction of nanobody with **1** (no modification observed). Conditions: nanobody (10 μM), $CuCl_2$ $2H_2O$ (50 μM), **1** (100 μM) in NMM buffer (50 mM pH 7.4, 0.2 M NaCl), 37 °C, 1.5 hours. **c**. nanobody **(C-*CAST*)** protein MS analysis. **d**. Reaction of nanobody **(C-*CAST*)** with **1.** Conditions: nanobody **(C-*CAST*)** (10 μM), $CuCl_2$ $2H_2O$ (50 μM), **1** (100 μM) in NMM buffer (50 mM pH 7.4, 0.2 M NaCl), 37 °C, 1.5 hours. **e**. Reaction of nanobody **(C-*CAST*)** with **1A**. **f**. Reaction of nanobody **(C-*CAST*)** with **1B**. **g**. Reaction of nanobody **(C-*CAST*)** with **1C**. Conditions: nanobody **(C-*CAST*)**(10 μM), $CuCl_2$ $2H_2O$ (50 μM), **1A, 1B** or **1C** (500 μM) in NMM buffer (50 mM, pH 7.4, 0.2 M NaCl), 37 °C, 1.5 hours. **h.** Reaction of nanobody **(N-*CAST*)** with **1**. Conditions: nanobody **(N-*CAST*)** (10 μM), $CuCl_2 \cdot 2H_2O$ (100 μM),**1** (100 μM) in NMM buffer (50 mM, pH 7.4, 0.2 M NaCl), 37 °C, 1.5 hours. **i.** Reaction of nanobody **(**G^{46}**-*CAST*)** with **1.** Conditions: nanobody **(**G^{46}**-*CAST*)** (10 μM), $CuCl_2 \cdot 2H_2O$ (100 μM),**1** (200 μM) in NMM buffer (50 mM, pH 7.4, 0.2 M NaCl), 37 °C, 1.5 hours.

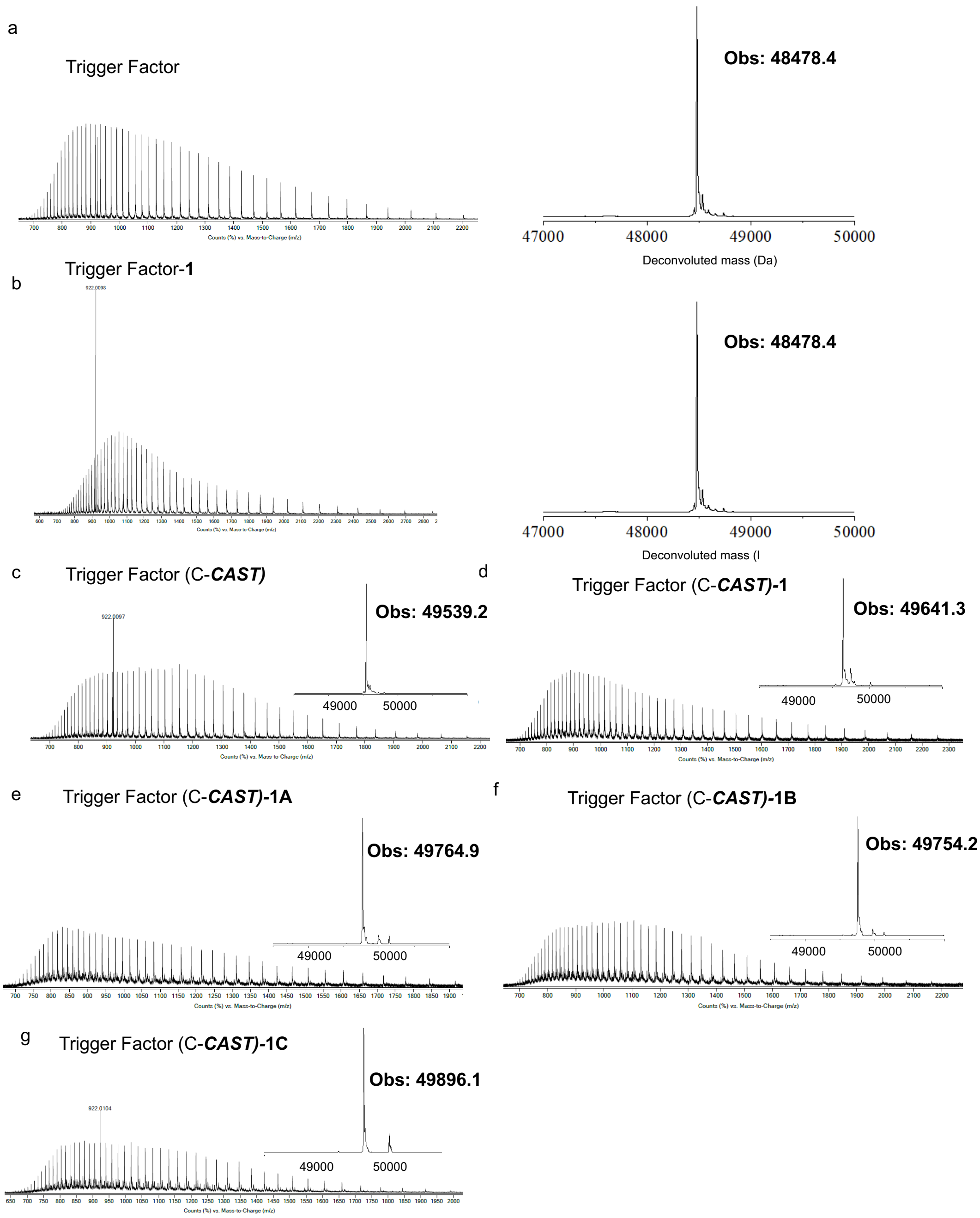

Supplementary Figure 28. **The ESI ion series/deconvolution MS spectra: reactions between trigger factor and 1, 1A, 1B, 1C**. **a**. Trigger factor protein MS analysis. **b**. Reaction of trigger factor with **1** (no modification observed). Conditions: trigger factor (5 µM), $CuCl_2\cdot 2H_2O$ (50 µM), **1** (100 µM) in NMM buffer (50 mM, pH 7.4, 0.2 M NaCl), 37 °C, 2 hours. **c**. Trigger factor **(C-*CAST*)** protein MS analysis. **d.** Reaction of trigger factor **(C-*CAST*)** with **1**. Conditions: trigger factor **(C-*CAST*)** (5 µM), $CuCl_2\cdot 2H_2O$ (50 µM), **1** (100 µM) in NMM buffer (50 mM, pH 7.4, 0.2 M NaCl), 37 °C, 2 hours. **e**. Reaction of trigger factor **(C-*CAST*)** with **1A**. **f**. Reaction of trigger factor **(C-*CAST*)** with **1B. g**. Reaction of trigger factor **(C-*CAST*)** with **1C**. Conditions: trigger factor **(C-*CAST*)** (5 µM), $CuCl_2\cdot 2H_2O$ (50 µM), **1A, 1B** or **1C** (500 µM) in NMM buffer (50 mM, pH 7.4, 0.2 M NaCl), 37 °C, 2 hours.

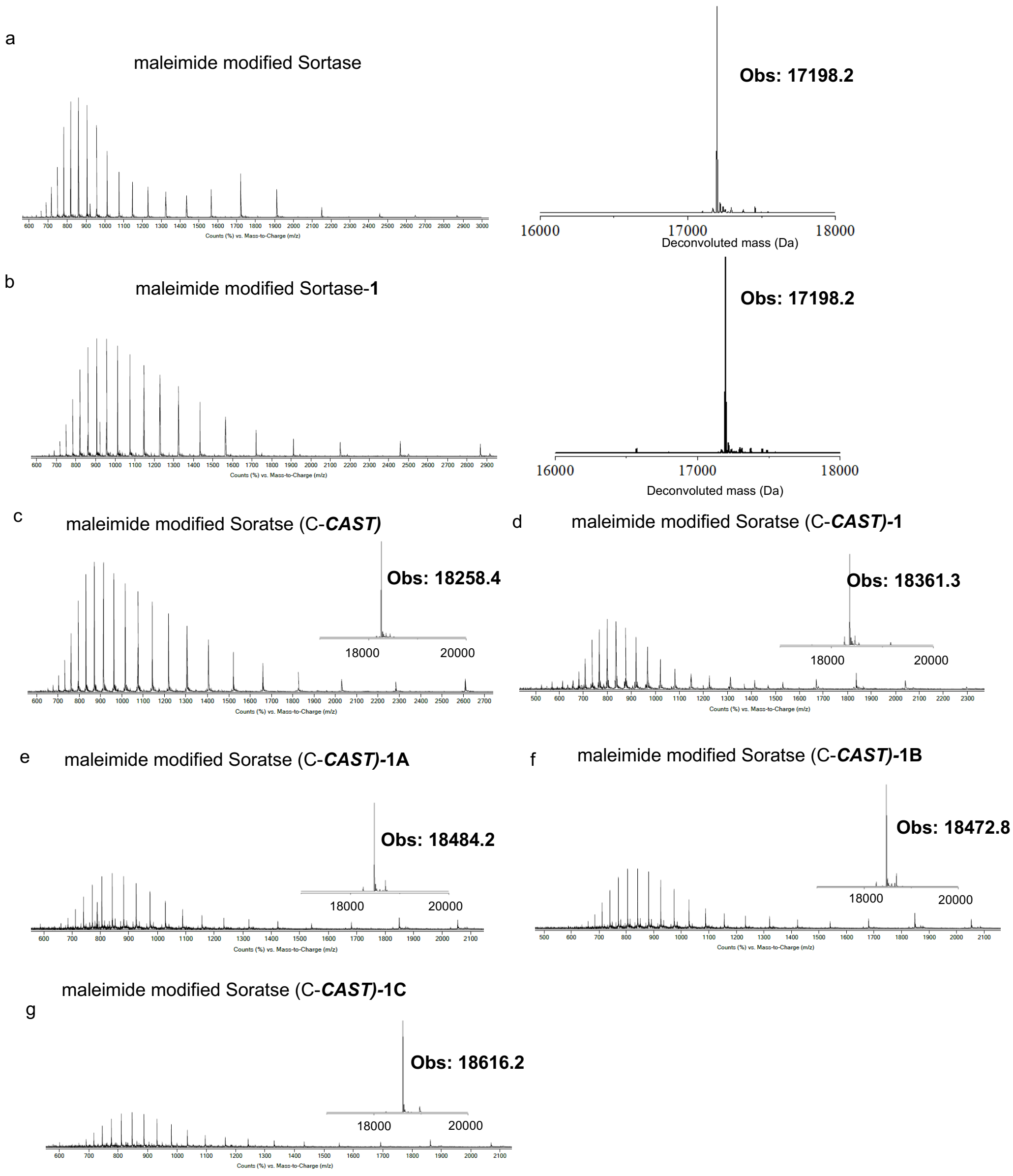

Supplementary Figure 29. ESI ion series/deconvolution MS spectra: reactions between maleimide-modified sortase and 1, 1A, 1B, 1C. **a**. Maleimide-modified Sortase MS analysis. **b**. Reaction of maleimide-modified sortase with **1** (no modification observed). Conditions: maleimide-modified sortase (10 µM), $CuCl_2 \cdot 2H_2O$ (50 µM), **1** (100 µM) in NMM buffer (50 mM, pH 7.4, 0.2 M NaCl), 37 °C, 30 minutes. **c**. maleimide-modified sortase **(C-*CAST*)** MS analysis. **d**. Reaction of maleimide-modified sortase **(C-*CAST*)** with **1**. Conditions: maleimide-modified sortase **(C-*CAST*)** (10 µM), $CuCl_2 \cdot 2H_2O$ (50 µM), **1** (100 µM) in NMM buffer (50 mM, pH 7.4, 0.2 M NaCl), 37 °C, 30 minutes. **e**. Reaction of maleimide-modified sortase **(C-*CAST*)** with **1A**. **f**. Reaction of maleimide-modified sortase **(C-*CAST*)** with **1B. g**. Reaction of maleimide-modified sortase **(C-*CAST*)** with **1C**. Conditions: maleimide-modified sortase **(C-*CAST*)** (10 µM), $CuCl_2 \cdot 2H_2O$ (50 µM), **1A, 1B** or **1C** (500 µM) in NMM buffer (50 mM, pH 7.4, 0.2 M NaCl), 37 °C, 30 minutes.

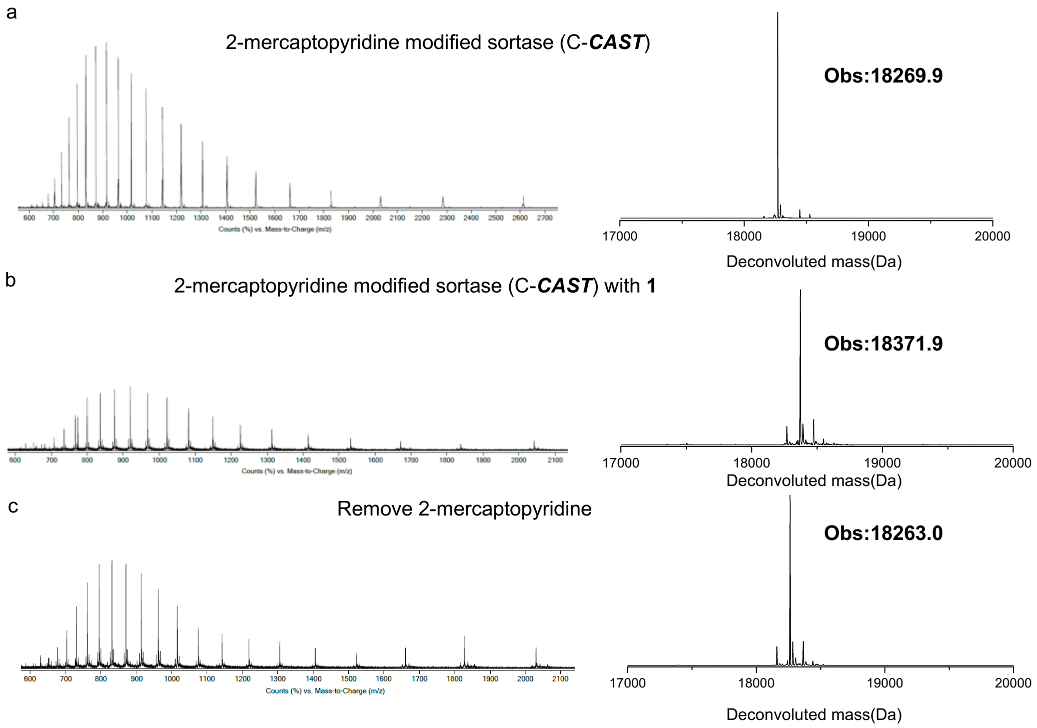

Supplementary Figure 30. Conjugation reaction between 2-mercaptopyridine-modified sortase (C-*CAST*) and 1. Cysteine residue of sortase was blocked with 2-mercaptopyridine before the ***CAST*** conjugation reaction. Sortase (C-***CAST***) (50 µM) was incubated with 2,2′-dipyridyl disulfide (1 mM) in PBS at r.t. for 1 hour, then PBS buffer was changed to NMM buffer (50 mM, pH 7.4, 0.2 M NaCl) using dialysis. **a**. 2-mercaptopyridine-modified Sortase (C-***CAST***) MS analysis. **b**. Reaction of 2-mercaptopyridine-modified sortase (C-***CAST***) with **1**. Conditions: 2-mercaptopyridine-modified sortase (C-***CAST***) (10 µM), $CuCl_2 \cdot 2H_2O$ (50 µM), **1** (100 µM) in NMM buffer (50 mM, pH 7.4, 0.2 M NaCl), 37 °C, 30 minutes. **c**: 20 mM TCEP was added to remove the 2-mercaptopyridine temporary protecting group on sortase Cys residue, the crude reaction product was analyzed using LC-MS

SDS-PAGE analysis of *CAST* fusion proteins:

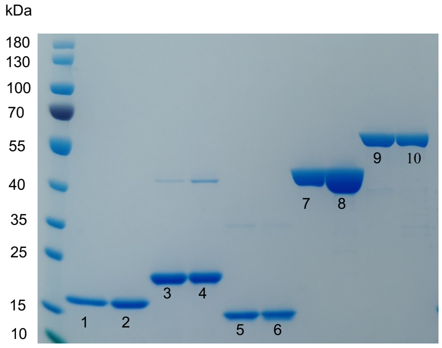

Lane 1:SMT3 (C-***CAST)***
Lane 2:SMT3(C-***CAST)*** after modification with **1**
Lane 3:maileimide modified soratse (C-***CAST)***
Lane 4:maileimide modified soratse (C-***CAST)*** after modification with **1**
Lane 5:nanobody (C-***CAST)***
Lane 6:nanobody (C-***CAST)*** after modification with **1**
Lane 7:MBP (C-***CAST)***
Lane 8:MBP (C-***CAST)*** after modification with **1**
Lane 9:Trigger factor (C-***CAST)***
Lane 10:Trigger factor (C-***CAST)*** after modification with **1**

Supplementary Figure 31. SDS-PAGE analysis of *CAST* fusion proteins before and after conjugation reaction. Detailed reaction conditions for each protein can be found in previous reaction procedure descriptions. Source data are provided as a Source Data file.

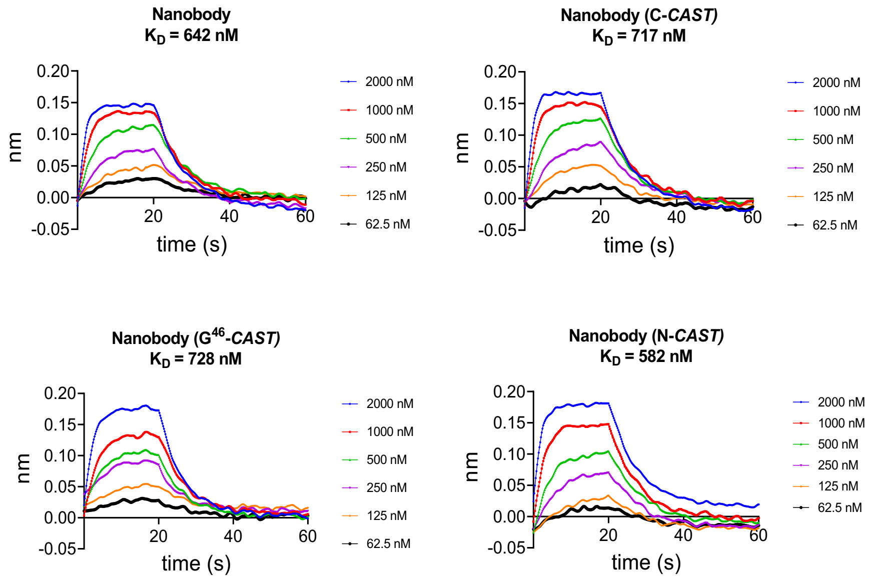

Supplementary Figure 32 Binding affinity characterization of *CAST*-nanobody with human serum albumin using biolayer interferometry. *CAST* insertion at different locations of nanobody didn't significantly alter the binding affinity to human serum albumin.

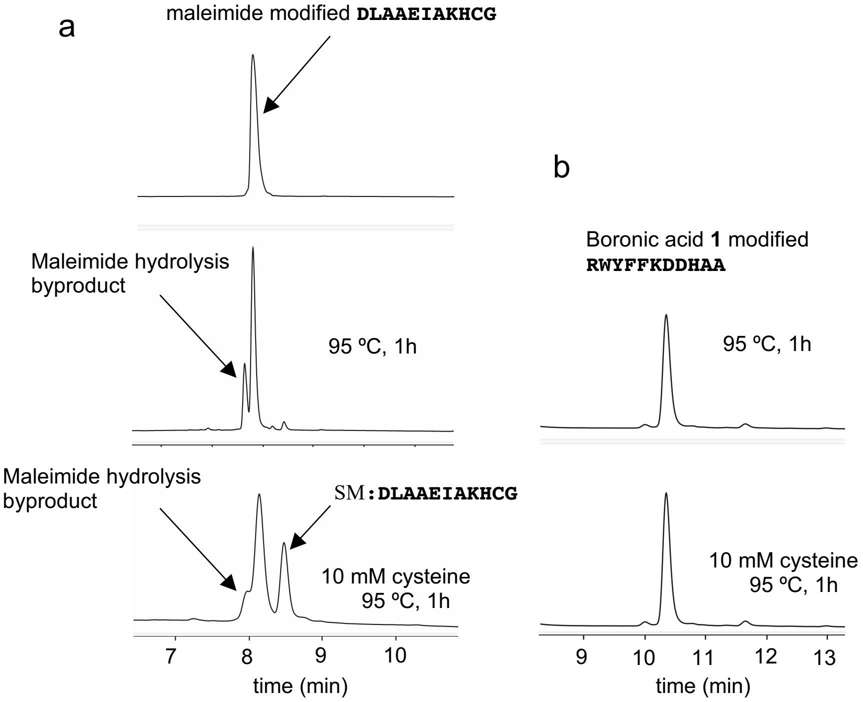

Supplementary Figure 33. The stability comparison of boronic acid 1 modified `RWYFFKKDDHAA` with maleimide modified `DLAAEIAKHCG` at different conditions. a. To a solution of peptide `DLAAEIAKHCG` (1μL of 5 mM stock solution in water, 0.05 mM final concentration) in PBS buffer (89 μL of 10 mM stock solution, pH 7.4), maleimide (10 μL of 50 mM stock solution in DMSO, 5 mM final concentration) were added subsequently. The mixture was vortexed and shaken for 30 minutes at room temperature. After removing

excessive maleimide, the product was incubated at 95 °C for 1 hour in PBS or incubated in 10 mM cysteine at 95 °C for 1 hour in PBS. **b.** To a solution of `RWYFFKKDDHAA` (1 µL of 5 mM stock solution in water, 0.05 mM final concentration) in NMM buffer (97 µL of 50 mM stock solution, pH 7.4), boronic acid **1** (1 µL of 50 mM stock solution in DMSO, 0.5 mM final concentration) and $CuCl_2 \cdot 2H_2O$ (1 µL of 15 mM stock solution in water, 0.15 mM final concentration) were added subsequently. The mixture was vortexed and shaken for 15 minutes at 37 °C. Then the purified modified `RWYFFKKDDHAA` was incubated at 95 °C for 1 hour in PBS or incubated in 10 mM cysteine at 95 °C for 1 hour in PBS.

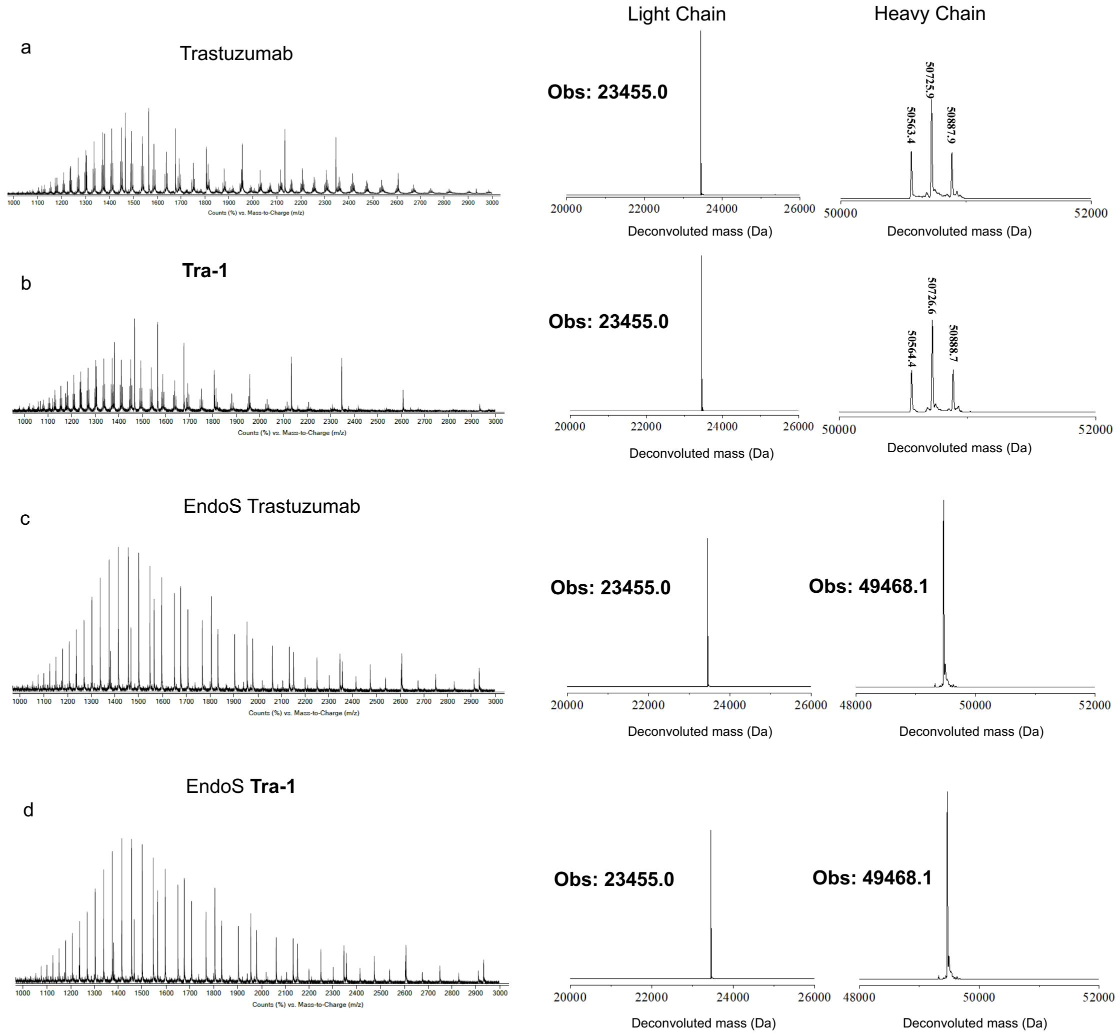

Supplementary Figure 34. The ESI ion series/deconvolution MS spectra: reactions between trastuzumab and 1. a. trastuzumab. **b**. Reaction of trastuzumab with **1** (no modification was observed either on the light chain or on the heavy chain). Conditions: trastuzumab (4 µM), $CuCl_2 \cdot 2H_2O$ (20 µM), **1** (250 µM) in NMM buffer (50 mM, pH 7.4, 0.2 M NaCl), 37 °C, 2 hours. **c**. trastuzumab was treated with endoglycosidase (EndoS) to remove the N-linked glycans before LC–MS analysis. **d**. Reaction of trastuzumab with **1**. The modified products were treated with endoglycosidase (EndoS) to remove the N-linked glycans before LC–MS analysis. (No modification observed either on light chain or on heavy chain).

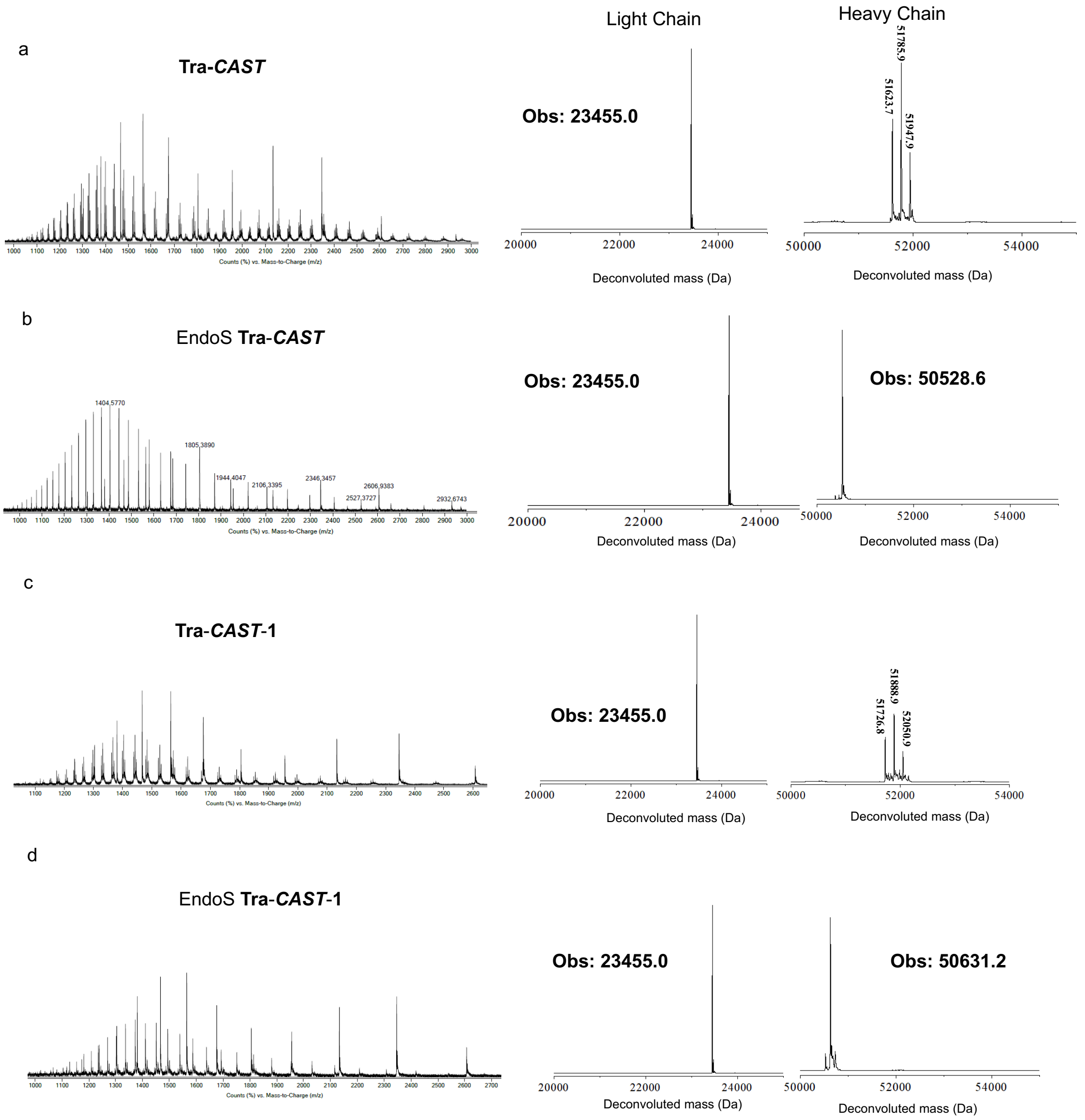

Supplementary Figure 35. ESI ion series/deconvolution MS spectra: reactions between Tra-*CAST* and 1. a. **Tra-*CAST*** protein MS analysis. **b**. **Tra-*CAST*** was treated with endoglycosidase (EndoS) to remove the N-linked glycans before LC–MS analysis. **c**. Reaction of **Tra-*CAST*** with **1.** Conditions: **Tra-*CAST*** (4 μM), $CuCl_2 \cdot 2H_2O$ (20 μM), **1** (250 μM) in NMM buffer (50 mM, pH 7.4, 0.2 M NaCl), 37 °C, 2 hours. **d**. Reaction of **Tra-*CAST*** with **1**. The modified products were treated with endoglycosidase (EndoS) to remove the N-linked glycans before LC–MS analysis.

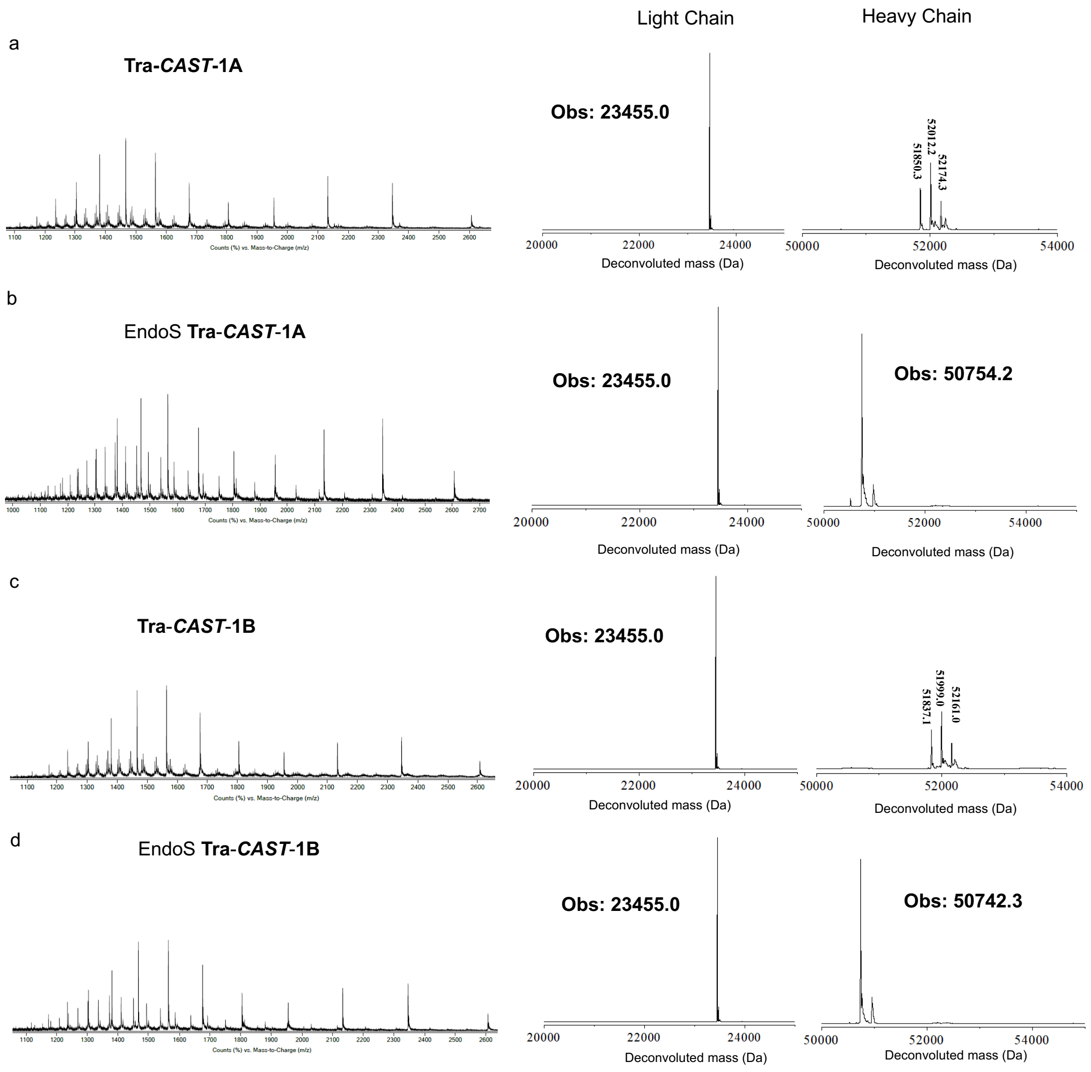

Supplementary Figure 36. ESI ion series/deconvolution MS spectra: reactions between Tra-*CAST* and 1A and 1B. a. Reaction of **Tra-*CAST*** with **1A**. Conditions: **Tra-*CAST*** (4 µM), $CuCl_2{\cdot}2H_2O$ (20 µM), **1** (250 µM) in NMM buffer (50 mM, pH 7.4, 0.2 M NaCl), 37 °C, 4 hours. **b**. The **1A**-modified products were treated with endoglycosidase (EndoS) to remove the N-linked glycans before LC–MS analysis. **c**. Reaction of **Tra-*CAST*** with **1B.** Conditions: **Tra-*CAST*** (4 µM), $CuCl_2{\cdot}2H_2O$ (20 µM), **1B** (250 µM) in NMM buffer (50 mM, pH 7.4, 0.2 M NaCl), 37 °C, 4 hours. **d**. The **1B**-modified products were treated with endoglycosidase (EndoS) to remove the N-linked glycans before LC–MS analysis.

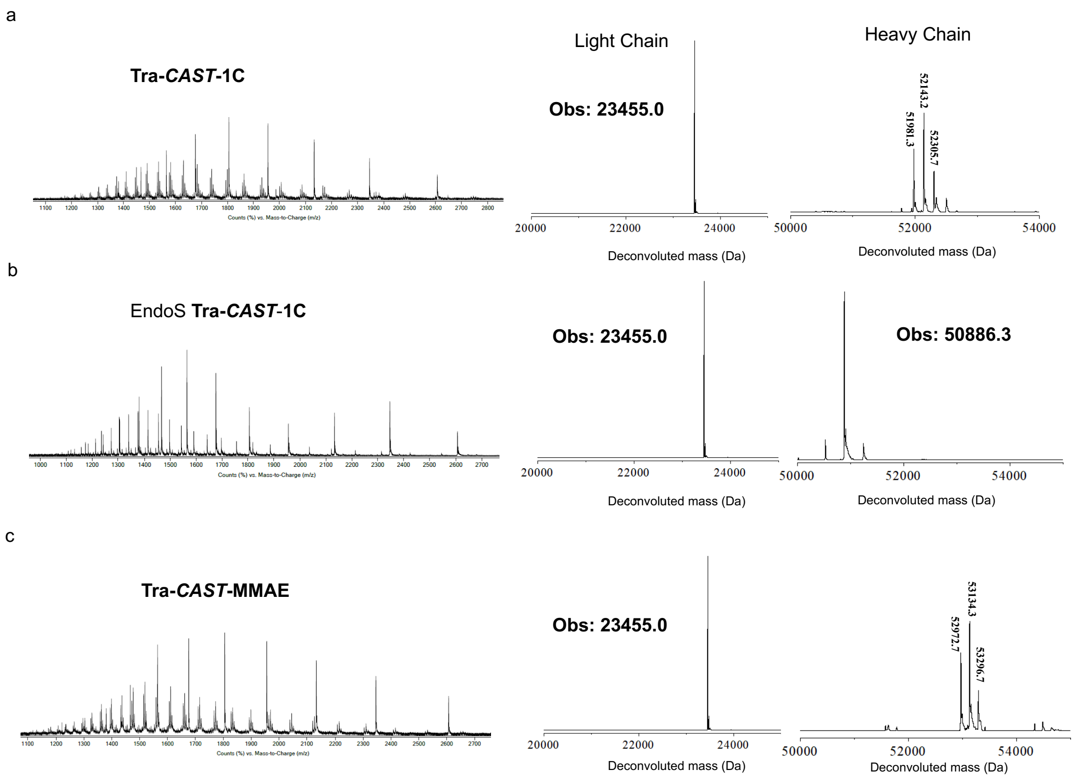

Supplementary Figure 37. ESI ion series/deconvolution MS spectra: reactions between trastuzumab-*CAST* and 1C and SBA-MMAE. a. Reaction of **Tra-*CAST*** with **1C**. Conditions: **Tra-*CAST*** (4 µM), $CuCl_2 \cdot 2H_2O$ (20 µM), **1C** (250 µM) in NMM buffer (50 mM, pH 7.4, 0.2 M NaCl), 37 °C, 4 hours. **b**. The **1C**-modified products were treated with endoglycosidase (EndoS) to remove the N-linked glycans before LC–MS analysis. **c**. Reaction of **Tra-*CAST*** with **SBA-MMAE** conditions: **Tra-*CAST*** (4 µM), $CuCl_2 \cdot 2H_2O$ (30 µM), **SBA-MMAE** (500 µM), 10% DMF in NMM buffer (50 mM, pH 7.4, 0.2 M NaCl), 37 °C, 7 hours.

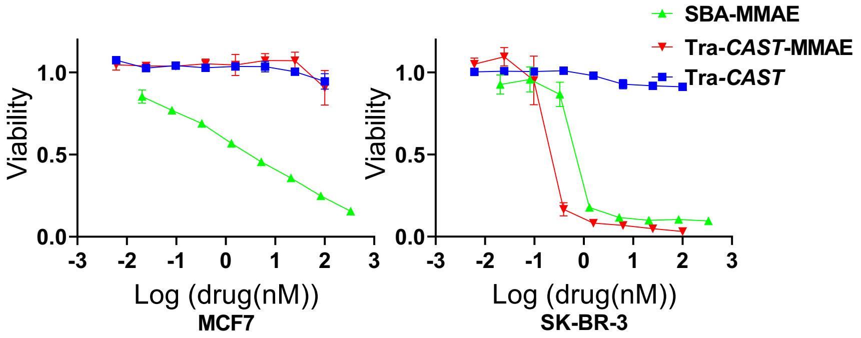

Supplementary Figure 38. **In vitro antitumor activity of Tra-*CAST*-MMAE. Tra-*CAST*-MMAE** effectively killed HER2-positive SK-BR-3 cells but only showed minimal toxicity against HER2-negative MCF7 cells.

8. Exploration of similar *CAST* sequence with high stability in vitro

a. Reaction efficiency of similar *CAST* sequences

Supplementary Table 16. Reaction yields of similar CAST sequences. The phenyl group, PEG2 linker and the last three residues (-SPG-) of Fc domain were attached to the N termini of these peptide sequences to facilitate analysis. Conjugation conditions: To a solution of peptide (1 µL of 5 mM stock solution in water, 0.05 mM final concentration) in NMM buffer (97 µL of 50 mM stock solution, pH 7.4), boronic acid reagent (1 µL of 50 mM stock solution in DMSO, 0.5 mM final concentration) and $CuCl_2 \cdot 2H_2O$ (1 µL of 15 mM stock solution in water, 0.15 mM final concentration) were added subsequently. The mixture was vortexed and shaken for 5 min at 37 °C.

Sequence	Yield
Phenyl-PEG$_2$-SPG-NAPDDHAA	88%
Phenyl-PEG$_2$-SPG-KAQDDHAA	88%
Phenyl-PEG$_2$-SPG-FFKKDDHAA	88%
Phenyl-PEG$_2$-SPG-SHAQDDHAA	85%
Phenyl-PEG$_2$-SPG-INDPDDHAA	83%
Phenyl-PEG$_2$-SPG-SKAQDDHAA	81%
Phenyl-PEG$_2$-SPG-NKKDDHAA	80%
Phenyl-PEG$_2$-SPG-NAQDDHAA	79%
Phenyl-PEG$_2$-SPG-IAPDDHAA	78%
Phenyl-PEG$_2$-SPG-HAQDDHAA	78%
Phenyl-PEG$_2$-SPG-LKQDDHAA	73%
Phenyl-PEG$_2$-SPG-MEQDDHAA	70%
Phenyl-PEG$_2$-SPG-IAQDDHAA	70%
Phenyl-PEG$_2$-SPG-IKAQDDHAA	65%
Phenyl-PEG$_2$-SPG-NEQDDHAA	63%

b.Plasma/Serum stability determination

Tra-*CASTi*-MMAE plasma stability

Tra-*CASTi*-MMAE (100 µg/mL, 1.2 µL in PBS) was added to BALB/c mouse plasma (98.8 µL). The sample was then incubated at 37 °C and aliquoted at different time points to store at −80 °C for later use. The **Tra-*CASTi*-MMAE** stability was then assessed using a sandwich ELISA assay. In the sandwich ELISA assay, homemade HER2 protein (100 ng per well) was used to coat the high-binding 96-well plate (Corning) overnight at 4 °C. 200 µL PBST (2% BSA, 0.05% Tween 20) was then used to do the blocking at room temperature for 2 hours. The aliquoted ADC samples (100 µL in PBS-T containing 2% BSA) were then added to incubate at 4 °C overnight. PBS-T buffer was then used to wash the plate four times. 100 µL of rabbit anti-human IgG antibody (1:5000) was used to detect trastuzumab (room temperature, 2 hours incubation). After washing it four times. 100 µL secondary goat anti-rabbit (1:5000) was added and then washed four times with 200 µL PBS-T before adding 100 µL the TMB substrate. After color was developed for 10−30 minutes, 100 µL of 2 M HCl was added to each well to stop the reaction. Then the absorbance at 450 nm was recorded using a plate reader (Thermo Varioskan LUX). Concentrations were calculated based on a standard curve. MMAE conjugate stability assays were performed in the same manner using homemade human HER2 (100 ng per well) for plate coating, mouse anti-MMAE antibody (1:5000), and goat anti-mouse IgG–HRP conjugate (1:5000) as secondary detection antibodies, respectively.

Peptide in vitro serum/plasma stability determination

Fresh blood was obtained from Male BALB/c mice (8 weeks old) from the Laboratory Animal Resources Center of Westlake University. The serum was prepared by centrifugation at 1500 g for 10 min after standing at room temperature for 30 min. The plasma was prepared by centrifugation at 1500 g for 20 min. Peptide was individually incubated with fresh serum at 2 mM at 37°C respectively. Samples were taken at 0 hour, 3 hours, and 21 hours. Then acetonitrile at 75% final concentration was added to serum samples to precipitate plasma proteins, precipitates were removed by centrifugation at 12000 g for 5 min. The supernatant was diluted 20 times with 0.1%TFA/H_2O (v/v) and analyzed by LC/MS.

Supplementary Table 17. Serum stability assessment in vitro. The phenyl group, PEG$_2$ linker and the last three residues (-SPG-) of the Fc domain were attached to the N termini of these peptide sequences to facilitate cleavage analysis. Different cleavage products are listed in the table, followed by the percentage of each cleaved product; backslash indicates the cleavage site. N/A: not applied. The ***CAST*** peptide is colored orange, and the peptide with the highest serum stability is colored green.

Sequences	3 hours		20 hours	
	Cleaved products	Percentage	Cleaved products	Percentage
Phenyl-PEG$_2$-SPG-NAQDDHAA	*Phenyl-PEG*$_2$-SPG/	70%	N/A	
	Phenyl-PEG$_2$-SP/	30%		
	Starting material	0%		
Phenyl-PEG$_2$-SPG-MEQDDHAA	*Phenyl-PEG*$_2$-SPG/	30%	*Phenyl-PEG*$_2$-SPG/	80%
	Phenyl-PEG$_2$-SP/	25%	*Phenyl-PEG*$_2$-SP/	15%
	Starting material	45%	Starting material	5%
Phenyl-PEG$_2$-SPG-NAPDDHAA	Starting material	95%	*Phenyl-PEG*$_2$-SPG/	50%
			Phenyl-PEG$_2$-SP/	50%
Phenyl-PEG$_2$-SPG-NEQDDHAA	Starting material	95%	*Phenyl-PEG*$_2$-SP/	10%
	Phenyl-PEG$_2$-SP/	5%	*Phenyl-PEG*$_2$-SPG/	80%
			Starting material	10%
Phenyl-PEG$_2$-SPG-KAQDDHAA	*Phenyl-PEG*$_2$-SPG/	50%		
	Phenyl-PEG$_2$-SP/	50%		
Phenyl-PEG$_2$-SPG-SKAQDDHAA	*Phenyl-PEG*$_2$-SPG/	25%	*Phenyl-PEG*$_2$-SPG/	30%
	Phenyl-PEG$_2$-SP/	55%	*Phenyl-PEG*$_2$-SP/	70%
	Starting material	20%		
Phenyl-PEG$_2$-SPG-INDPDDHAA	*Phenyl-PEG*$_2$-SP/	5%	*Phenyl-PEG*$_2$-SPG/	30%
	Starting material	95%	Starting material	60%
			Phenyl-PEG$_2$-SP/	10%
Phenyl-PEG$_2$-SPG-FFKKDDHAA	*Phenyl-PEG*$_2$-SP/	10%	N/A	
	Phenyl-PEG$_2$-SPG-F/	55%		
	Phenyl-PEG$_2$-SPG-FF/	10%		
	Phenyl-PEG$_2$-SPG/	10%		
	Starting material	15%		
Phenyl-PEG$_2$-SPG-HAQDDHAA	*Phenyl-PEG*$_2$-SP/	30%	N/A	
	Phenyl-PEG$_2$-SPG/	70%		
Phenyl-PEG$_2$-SPG-SHAQDDHAA	*Phenyl-PEG*$_2$-SPG/	38%	N/A	
	Phenyl-PEG$_2$-SP/	62%		
Phenyl-PEG$_2$-SPG-IAPDDHAA	*Phenyl-PEG*$_2$-SPG/	5%	*Phenyl-PEG*$_2$-SPG-IAPD/	80%
	Starting material	95%	*Phenyl-PEG*$_2$-SPG/	16%
			Phenyl-PEG$_2$-SP/	4%
Phenyl-PEG$_2$-SPG-IAQDDHAA	*Phenyl-PEG*$_2$-SPG/	85%	*Phenyl-PEG*$_2$-SPG/	99%
	Starting material	15%		
Phenyl-PEG$_2$-SPG-IKAQDDHAA	*Phenyl-PEG*$_2$-SPG-I/	20%	*Phenyl-PEG*$_2$-SPG/	80%
	Phenyl-PEG$_2$-SPG/	30%	*Phenyl-PEG*$_2$-SP/	20%
	Phenyl-PEG$_2$-SPG-IKA/	10%		
	Phenyl-PEG$_2$-SP/	30%		
	Starting material	10%		
Phenyl-PEG$_2$-SPG-NKKDDHAA	*Phenyl-PEG*$_2$-SPG/	80%	*Phenyl-PEG*$_2$-SPG/	95%
	Starting material	20%		
Phenyl-PEG$_2$-SPG-LKQDDHAA	*Phenyl-PEG*$_2$-SPG/	95%	N/A	

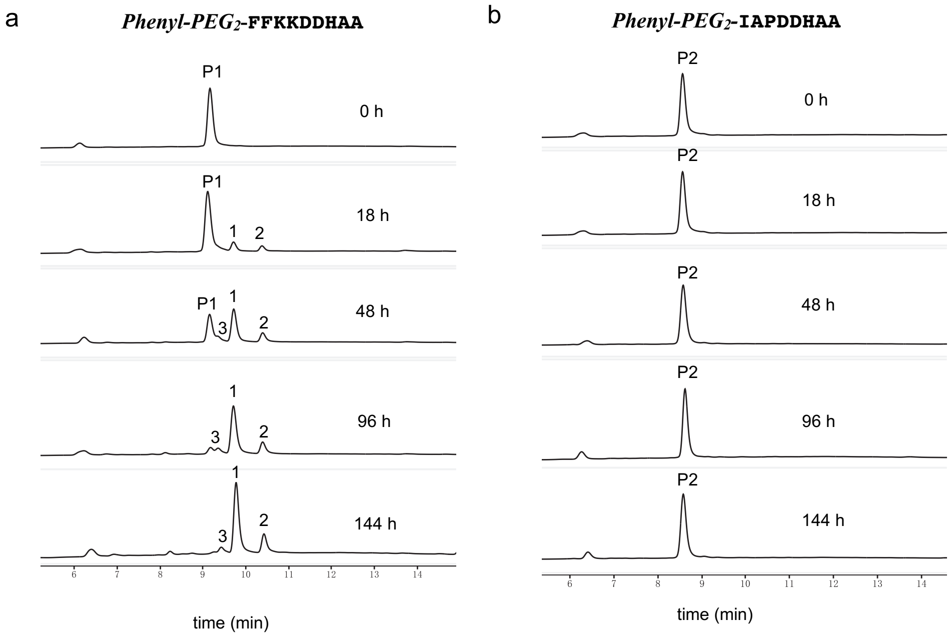

1. *Phenyl-PEG*$_2$-FFK/
2. *Phenyl-PEG*$_2$-FF/
3. *Phenyl-PEG*$_2$-F/ mixed with *Phenyl-PEG*$_2$-FFKK

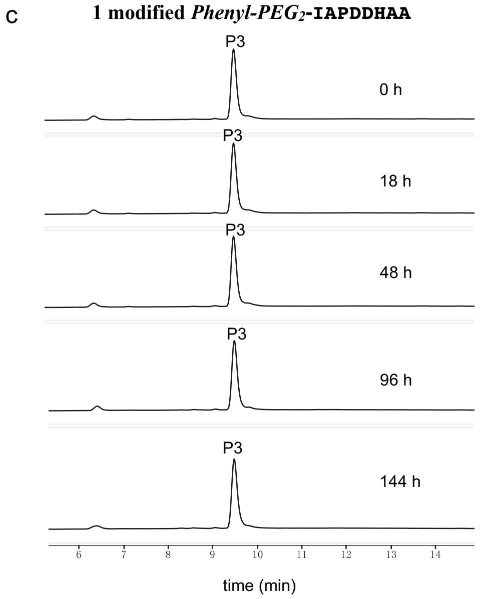

Supplementary Figure 39. Plasma stability assessment in vitro. IAPDDHAA has higher plasma stability than FFKKDDHAA in vitro. a. Control: *Phenyl-PEG*$_2$-FFKKDDHAA plasma stability over time, cleaved products are listed below the figure. **b.** *Phenyl-PEG*$_2$-IAPDDHAA plasma stability over time. **c. 1** modified IAPDDHAA plasma stability over time.

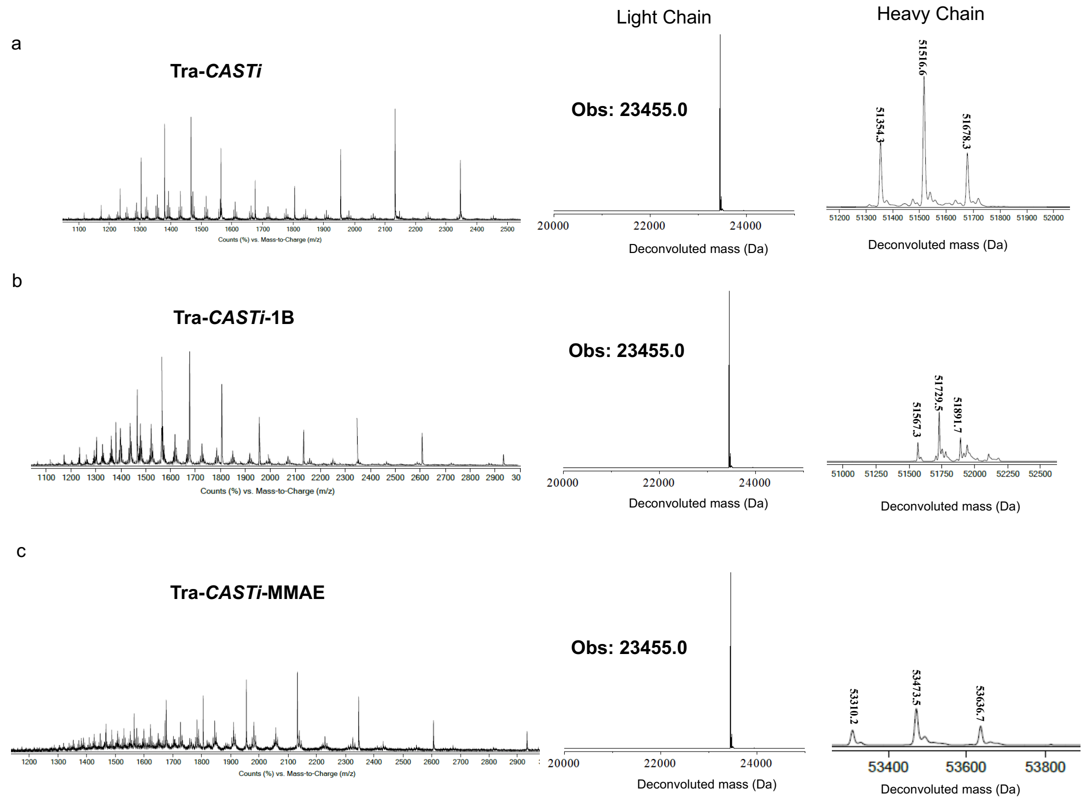

Supplementary Figure 40. ESI ion series/deconvolution MS spectra: reactions between Tra-*CASTi* and DBCO-MMAE(h) a. **Tra-*CASTi***. **b**. Reaction of **Tra-*CASTi*** with **1B.** Conditions: **Tra-*CASTi*** (4 μM), $CuCl_2 \cdot 2H_2O$ (20 μM), **1B** (250 μM) in NMM buffer (50 mM, pH 7.4, 0.2 M NaCl), 37 °C, 4 hours. **c**. Reaction of **1B**-modified **Tra-*CASTi*** with **h**. Conditions: **1B**-modified **Tra-*CASTi*** (4 μM), **h** (80 μM) in PBS (10 mM, pH 7.4), 37 °C, 2 hours.

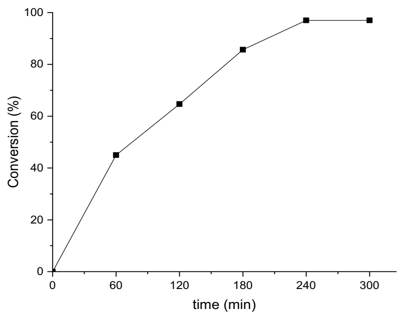

Supplementary Figure 41. Kinetics of Tra-*CASTi* reaction with 1B. Conjugation conditions: **Tra-*CASTi*** (2 μM), $CuCl_2 \cdot 2H_2O$ (20 μM), **1B** (250 μM) in NMM buffer (50 mM, pH 7.4, 0.2 M NaCl), the mixture was incubated at 37°C for 0 minutes, 60 minutes, 120 minutes, 180 minutes, 240 minutes before analysis.

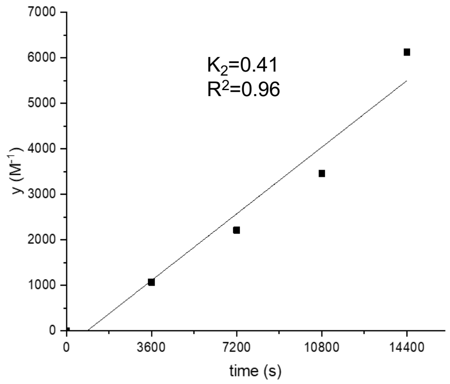

Supplementary Figure 42. Linear fitting of kinetics data for Tra-*CASTi* reaction with 1B. The second-order reaction rate of **Tra-*CASTi*** with **1B** was determined by fitting the data to the following equation:

$$y = \frac{\ln\left(\frac{[protein]_0[probe]_t}{[protein]_t[probe]_0}\right)}{([probe]_0 - [protein]_0)} = k_2 t$$

t= 0 s, 3600 s, 7200 s, 10800 s, 14400 s. $[\text{protein}]_0$ and $[\text{probe}]_0$ are the initial concentrations of the **Tra-*CASTi*** and **1B**; and $[\text{protein}]_t$ and $[\text{probe}]_t$ are the concentrations of the **Tra-*CASTi*** and **1B** at time t.

9. Determination of copper concentration in Tra-*CASTi*-MMAE

1. Materials

ICP-MS tune solution and nitric acid (trace metal grade) were from Alfa Aesar Company (USA) and Fisher Chemical Company (Canada), respectively. Standard element solution was purchased from National Center of Analysis and Testing for Nonferrous and Electronic Materials (China). All element concentrations are shown in Supplementary Table 18. The standard solution was diluted into 20.0,10.0, 5.0, 2.0, 1.0, 0.5 ppb with 1% HNO_3 for calibration curve. Milli-Q water (18.2M Ω·cm) was used in all experiments. The sample and blank solutions were analyzed after filtering. The concentration of **Tra-*CASTi*-MMAE** was about 208 ppm (mg/L)

Supplementary Table 18 Element and corresponding concentrations.

Solution	Element	Concentration	Liquid Matrix
tune solution	Ba, Bi, Ce, Co, In, Li, U	1.00 ± 0.05 μg/L	2% HNO_3+0.5% HCl
standard element solution	Al, As, B, Ba, Be, Bi, Ca, Cd, Co, Cr, Cu, Fe, K, Li, Mg, Mn, Na, Ni, P, Pb, Se, Sr, V, Zn	1000.00 μg/ml	9% HNO_3

2. ICP-MS analysis

Copper (Cu) was quantified with internal standard method via inductively coupled plasma-mass spectrometry (ICP-MS, iCAPRQ, ThermoFisher) in kinetic energy discrimination mode. Some typical instrument parameters are listed in Supplementary Table 19. Data was acquired by Qtegra software (Version: 2.10.3324.131). The instrument was initially tuned by pumping tune solution to optimized sensitivity. Blank solution, calibration solution and sample solution were analyzed, and two Milli-water injections were inserted between calibration solution and sample solution. R^2 value for copper calibration curve is 0.9998 (Supplementary Figure 45). The detector intensity of sample solution is lower than that of 0.5 ppb standard solution, exceeding the detection limit of ICP-MS. Additionally, based the acquired standard curve, the concentrations of Cu in **Tra-*CASTi*-MMAE** 208 ppm (mg/L) was estimated to be 0.29 ppb (μg/L).

Supplementary Table 19 ICP-MS instrument operating conditions.

Operating parameters	Condition
RF power	1550 W
Sampling depth	5.0 mm
Auxilliary Flow (Ar)	0.80 L/min
Nebulizer Flow (Ar)	0.95 L/min
Cool Flow (Ar)	14 L/min
Spray Chamber temperature	2.7°C
Peristaltic Pump Speed	40
Replicates	3
Dwell time	0.05 s
Isotopes monitored	^{63}Cu , ^{45}Sc[a]

a internal standard

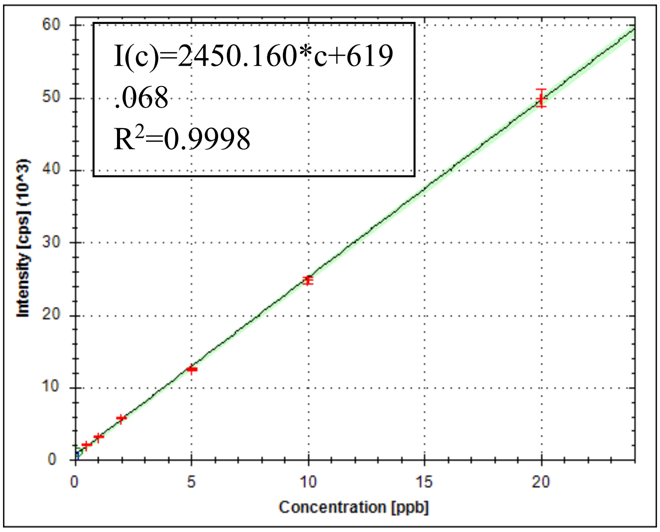

Supplementary Figure 43. The calibration curve for copper concentration quantification.

10. BioLayer interferometry binding assay

In vitro binding assays were performed using Fortebio Octet BioLayer Interferometry system at room temperature[6]. Briefly, AHC tips were dipped into 200 µL of antibody solution (10 ug/ml **Tra-*CAST***or **Tra-*CAST*-MMAE** in PBS with 0.1 % BSA and 0.02 % tween20) for the loading of antibodies. The tips loaded with antibody were sampled with recombinant HER2 (Sino biological 10004-H08H1-50) at various HER2 concentrations in PBS with 0.1% BSA and 0.02% tween 20 to obtain the association curve, buffer only serves as the reference. After association, the tips were dipped into PBS with 0.1 % BSA and 0.02 % tween 20 to obtain the dissociation curve. Following the protocols provided by Fortebio Biosystems, the association and dissociation curves of each sample were manually fitted using ForteBio DataAnalysis 12 to obtain the K_D. The final K_D was reported as the average of the K_D obtained from experiments with serially diluted HER2
Our selected nanobody binds to human serum albumin (HSA)[7], The functional impact of inserting ***CAST*** to nanobody was evaluated by measuring the affinity to human serum albumin using Biolayer Interferometry at room temperature. The anti-His tips dipped into 200ul of human HSA solution (10ug/ml HSA with his-tag in PBS with 0.02% tween 20) for the loading of HSA. The tips loaded with HSA were sampled with ***CAST***-nanobodies at various concentrations in PBS with 0.02% tween 20 to obtain the association curve. The buffer served as the reference. After association, the tips were dipped into PBS with 0.02% tween 20 to obtain the dissociation curve. After the emperiments, the association and dissociation curves of each sample were manually fitted to obtain the K_D. The final K_D was reported as the average of the K_D obtained from experiments with serial diluted nanobodies.

11. Cell assays

Cells were seeded in a 96-well white opaque plate at a density of 5×10^3/well (CHO) or 1×10^4/well (MCF7, SK-BR-3, SK-OV-3 or JIMT-1). Cells were allowed to attach for 24 hours at 37 °C and 5% CO_2 in humidified atmosphere. Cells were then treated with serial dilutions of **Tra-*CAST***, **Tra-*CAST*-MMAE** and **SBA-MMAE** or **Tra-*CASTi***, **Tra-*CASTi*-MMAE** and **DBCO-MMAE** for 96 hours (BT474, MCF7, SK-BR-3). Cell viability was determined using CellTiter Glo reagents (G7571) and was normalized to the control cells. Data was analyzed by Graphpad software, and the half-maximal effective concentration (EC50) value was calculated by fitting with the log(inhibitor) vs. response module.

12.Animal model preparation and in vivo antitumor experiment

All procedures were approved by the Institutional Animal Care and Use Committee of Zhejiang University or Westlake University. Female Balb/c nude mice (age, 4 weeks) purchased from Ziyuan Laboratory Animal lnc. (Hangzhou, China). Mice were acclimated for 1 week before

the experiment and kept under standard laboratory conditions with food and water provided ad libitum. HER2-positive human ovarian xenograft tumor model was used to evaluate the antitumor effect of ADC compounds (**Tra-*CASTi*-MMAE**). Briefly, cultured SK-OV-3 cells were suspended in DMEM medium without serum and antibiotics. Mice received 100 μL subcutaneous injection of SK-OV-3 cell suspension (1 × 10^7/100μL). Tumor volume was calculated by using the following formula: Tumor volume = 0.52 × Length × $Width^2$. When the volume of xenograft tumor reached average of 100-150 mm^3, the mice were randomly divided into four groups. The **Tra-*CASTi*-MMAE** protein (12 or 6 mg/kg) and control (**Tra-*CASTi***, 12 mg/kg; Fc, 12 mg/kg) were administered to mice via the tail vein on days 0, 7, 14 and 28. Tumor volume and body weight were monitored three times a week. When xenograft tumors grew to 1000 mm^3, the mice were killed.

Female NSG mice (age, 4 weeks) were purchased from Shanghai Jihui (Shjh) Laboratory Animals Care Co. Ltd. Briefly, cultured JIMT-1 cells were suspended in DMEM medium without serum and antibiotics. NSG mice received 100 μL subcutaneous injection of JIMT-1 cell suspension (1 × 10^7/100μL). Tumor volume was calculated by using the following formula: Tumor volume = 0.52 × Length × $Width^2$. When the volume of xenograft tumor reached average of 100-150 mm^3, the mice were randomly divided into two groups. The **Tra-*CASTi*-MMAE** protein (12 mg/kg) and control (**Tra-*CASTi***, 12 mg/kg) were intraperitoneally administered to mice on days 0, 7 and 14. Tumor volume and body weight were monitored twice a week. When xenograft tumors grew to 1000 mm^3, the mice were killed.

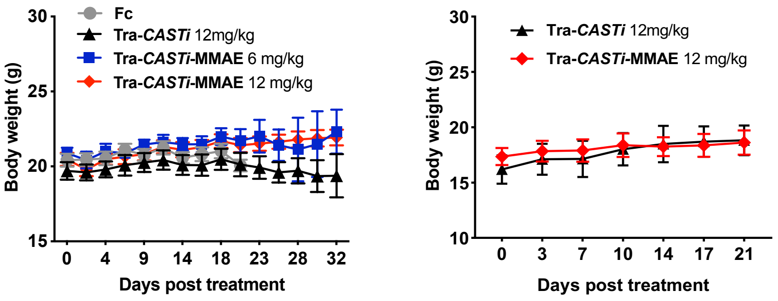

Supplementary Figure 44. Body weight after Tra-*CASTi*-MMAE administration. Left: Female Balb/c nude mice, n = 5 for 12 mg/kg Fc isotype control, n = 5 for 12 mg/kg Tra-***CASTi***, n = 4 for 6 mg/kg **Tra-*CASTi*-MMAE**, n = 6 for 12 mg/kg **Tra-*CASTi*-MMAE**; Right: female NSG mice, n = 6 for **Tra-*CASTi*-MMAE,** n = 6 for **Tra-*CASTi***, error bars represent s.e.m.

13. Supplementary figures and tables

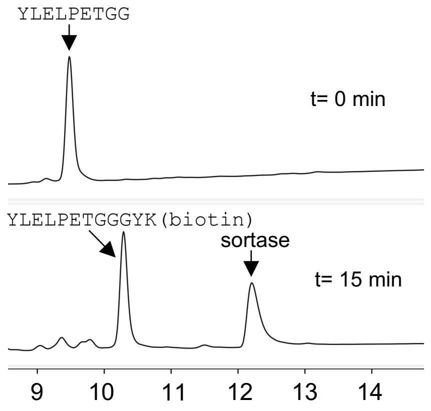

Figure. S45. Evolved sortase mediated peptide ligation. Condition: peptide YLELPETGG (50 μM), sortase (2.5 μM), GGGYK(biotin) (500 μM), 25 °C for 15 minutes, ~ 95 % yield.

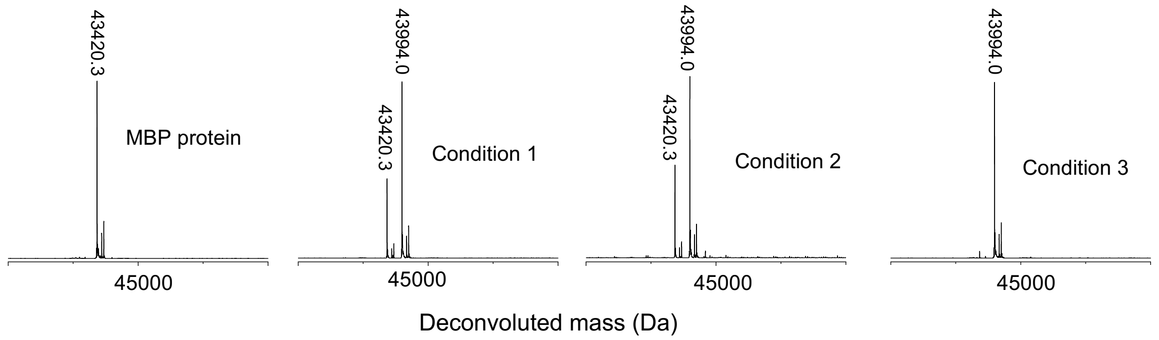

Figure. S46. Evolved sortase mediated MBP ligations. Condition 1: MBP (10 μM), sortase (1 μM), GGGYK(biotin)(500 μM), 25 °C for 2 hours, 75 % yield. Condition 2: MBP (20 μM), sortase (1 μM), GGGYK(biotin)(500 μM), 25 °C for 2 hours, 75 % yield. Condition 3: MBP (60 μM), sortase (20 μM), GGGYK(biotin)(500 μM), 25 °C for 30 minutes, > 95 % yield. Only when significant amount of sortase (33% of MBP) is added, the reaction can proceed to completion.

Supplementary Table 20: Proteomic analysis of ***CAST*** conjugation reaction products. The listed sequences showed modification by proteomic analysis, residues with red color are the modification sites.

SMT3(C-CAST)	Nanobody(C-CAST)	MBP(C-CAST)
MDSLRFLYDG	VATITHGTNT	VNKDKPLGAV
EQIGGFFK	FTISRDNAK	RFGGYAQSGL
LRFLYDGIRI	GGSDQVQLQE	AATGDGPDII
IQADQTPEDL		GDKGYNGLAE
DLDMEDNDIIE		IKNKHMNADT
RLMEAFAK		QDKLYPFTWD
KPETHINLK		NPPKTWEEIP
SDSEVNQEA		TSKVNYGVTV
NLKVSDGSSEI		VTVEHPDKLE

Following the proteomic analysis, we synthesized all these 21 peptides and performed the conjugation reactions, only one peptide (-VATITHGTNT-) showed ~1% modification, and all the other 20 peptides have no observable modification, reaction conditions: peptide (10 µM), $CuCl_2$ $2H_2O$ (50 µM), **1** (100 µM) in NMM buffer (50 mM pH 7.4, 0.2 M NaCl), 37 °C, 1 h.

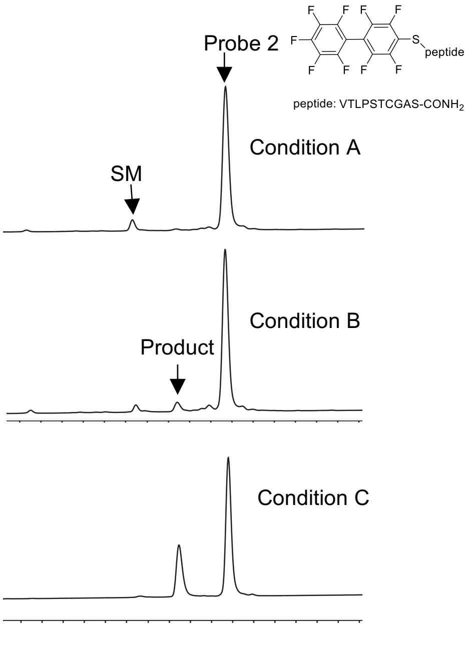

Supplementary Figure 47. *π*-clamp conjugaion on peptide. Peptide： NH_2-FCPFGLLKNK-$CONH_2$. Condition A： peptide (50 µM), probe 2 (500 µM) in 0.2 M phosphate, 20 mM TCEP, 37 °C, 30 minutes. ~ 2% yield. Condition B: peptide (50 µM), probe 2 (500 µM) in 0.2 M phosphate, 20 mM TCEP, 37 °C, 3 hours. ~ 55 % yield. Condition C： peptide (1 mM), probe 2 (5 mM) in 0.2 M phosphate, 20 mM TCEP, 37 °C, 30minutes. > 95% yield.[8]

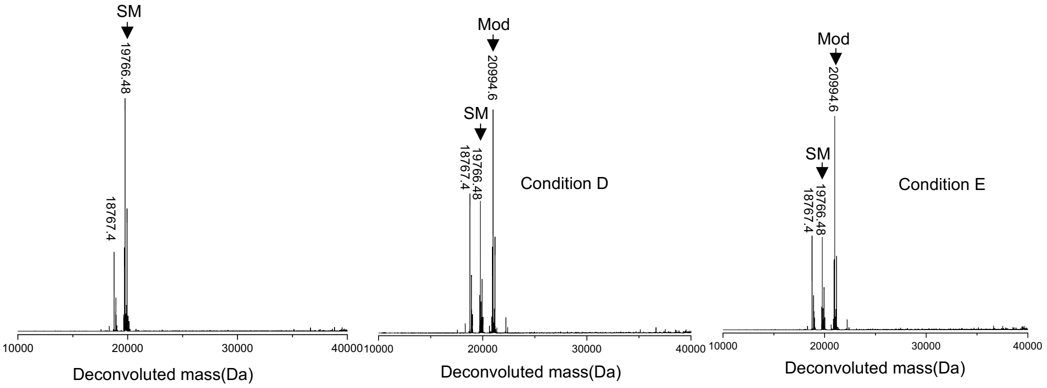

Supplementary Figure 48. **π-clamp conjugaion on π-clamp sortase**. Condition D: π-clamp sortase (38 μM), probe 2 (1 mM) in 0.2 M phosphate, 20 mM TCEP, 37 °C, 2 hours. Condition E: π-clamp sortase (10 μM), probe 2 (1 mM) in 0.2 M phosphate, 20 mM TCEP, 37 °C, 2 hours. SM: starting material, Mod: modified product.

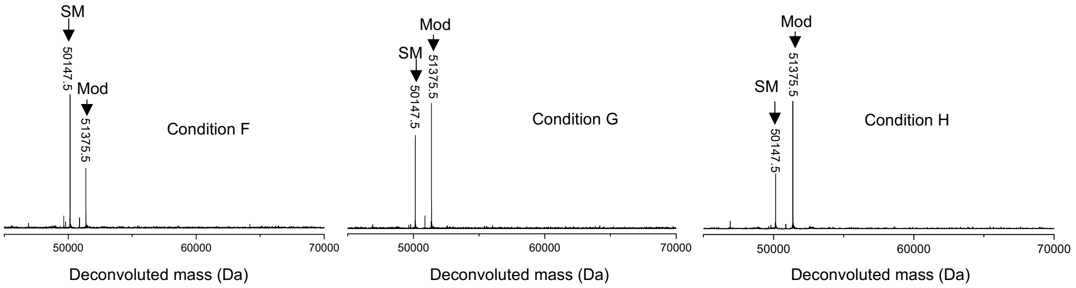

Supplementary Figure 49. **π-clamp conjugaion on π-clamp trastuzumab**. Light chain doesn't have π-clamp, no reaction observed, heavy chain reaction was analyzed using LC-MS and shown here. Condition F: π-clamp trastuzumab (4 μM), probe 2 (0.5 mM) in 0.2 M phosphate, 20 mM TCEP, 37 °C, 4 hours. Condition G: π-clamp trastuzumab (4 μM), probe 2 (1 mM) in 0.2 M phosphate, 20 mM TCEP, 37 °C, 4 hours. Condition H: π-clamp trastuzumab (12 μM), probe 2 (1 mM) in 0.2 M phosphate, 20 mM TCEP, 37 °C, 4 hours. SM: starting material, Mod: modified product.

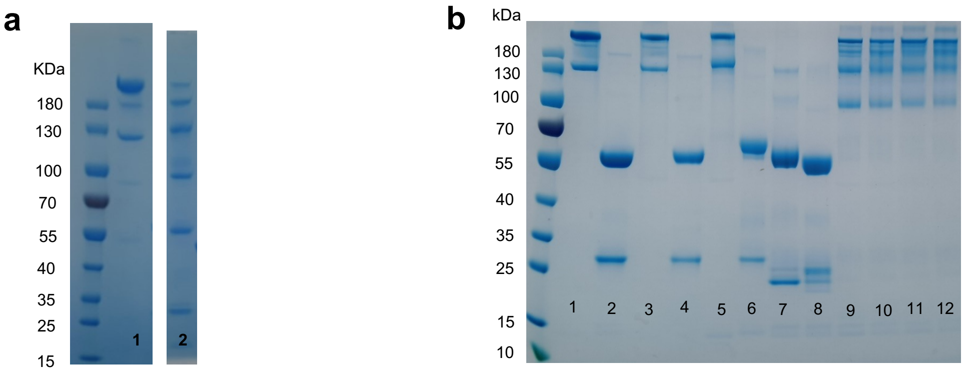

Lane 1:**Tra-*CASTi*** boiling 5 minutes
Lane 2:**Tra-*CASTi*** + 0.6 mM TCEP + 5.6 equiv. bis-sulfone-PEG$_3$-azide, boiling 5 minutes

Lane 1:**Tra-*CASTi***
Lane 2:**Tra-*CASTi*** + 10 mM DTT, boiling 5 minutes
Lane 3:**Tra-*CASTi*** modified with **1B**
Lane 4:**Tra-*CASTi*** modified with **1B** + 10 mM DTT, boiling 5 minutes
Lane 5:**Tra-*CASTi*-MMAE**
Lane 6:**Tra-*CASTi*-MMAE**+10 mM DTT, boiling 5 minutes
Lane 7:**Tra-*CASTi*** + 0.6 mM TCEP
Lane 8:**Tra-*CASTi*** + 0.6 mM TCEP, boiling 5 minutes
Lane 9:**Tra-*CASTi*** + 0.6 mM TCEP + 10 equiv. bis-sulfone-PEG$_3$-azide
Lane 10:**Tra-*CASTi*** + 0.6 mM TCEP + 20 equiv. bis-sulfone-PEG$_3$-azide
Lane 11:**Tra-*CASTi*** + 0.6 mM TCEP + 50 equiv. bis-sulfone-PEG$_3$-azide
Lane 12:**Tra-*CASTi*** + 0.6 mM TCEP + 100 equiv. bis-sulfone-PEG$_3$-azide

Supplementary Figure 50. **SDS-PAGE analysis of Tra-*CASTi* modification through *CAST* conjugation or disulfide rebridging. a. Tra-*CASTi*** modification using disulfide rebridging strategy at r.t. for 16 hours.[9] **b. Tra-*CASTi*** modification using ***CAST*** conjugation or disulfide rebridging. Disulfide rebridging conjugations procedure**:** dilute the **Tra-*CASTi*** solution to 3 mg/mL (20 μM) using reaction buffer (10 mM PBS, 20 mM EDTA), total volume is 15 μL. To the antibody solution add 5 mM TCEP (1.8 μL, 30 equiv. final 0.6 mM) and mix the solution using a vortexer. Incubate the reduction mixture at 40 °C for 1 hour. Cool the reduced antibody solution to 22 °C. To the reduced antibody solution add reagent solution (1.5 mM bis-sulfone-PEG$_3$-azide CAS: 1802908-01-5 in DMSO) 1.2 μL (5 equiv., final 100 μM), 2.4 μL (10 equiv., final 200 μM), 0.7 μL (9 mM bis-sulfone-PEG$_3$-azide in DMSO, 20 equiv., final 400 μM), 1.75 μL(50 equiv., final 1 mM), 3.5 μL(100 equiv., final 2 mM), mix the conjugation reaction using a vortexer and then incubate at r.t. for 22 hours. The lowest bands in lane 9-12 corresponds to incomplete conjugated antibody. Source data are provided as a Source Data file.

14.Protein reaction in cell lysate

Preparation of cell lysate[10]

BL21 cells were cultured in LB medium. 293F cells were cultured in SMM 293-TII medium .The cultured cells were collected by centrifugation. After removal of the supernatant, the cells were suspended in NMM buffer and lysed by sonication. Cell debris was separated by centrifugation. For BL21 cell lysate, 2 mM maileimide was added at 37°C for 1 hour to block free Cys. For 293F cell lysate, 2 mM maileimide was added at 37°C for 1 hour to block free Cys, 2 mM phenylboronic acid was also added at 37°C for 1 hour to block potential sugar interactions with boronic acid. Then the added small molecues were removed using dialysis. The obtained cell lysate was split into aliquots, frozen with liq. N_2, and stored in –80 °C.

Procedure for *CAST* fusion protein labeling in cell lysate

MBP (C-***CAST***):

For labeling in BL21 cell lysate, reaction conditions: MBP (C-***CAST***) (5 μM), **1C** (0.5 mM), $CuCl_2 \cdot 2H_2O$ (0.5 mM), BL21 cell lysate in NMM buffer (50 mM, pH 7.4, 0.2 M NaCl) r.t., 5 minutes.

For labeling in 293F cell lysate, reaction conditions: MBP (C-***CAST***) (2 μM), **1C** (1 mM), $CuCl_2 \cdot 2H_2O$ (1 mM), 293F cell lysate in NMM buffer (50 mM, pH 7.4, 0.2 M NaCl), 37 °C, 5 minutes.

Tra-*CASTi* :

For labeling in BL21 or 293F cell lysate, reaction conditions: **Tra-*CASTi*** (2 μM), **1C** (1 mM), $CuCl_2 \cdot 2H_2O$(1 mM), BL21 or 293F cell lysate in NMM buffer (50 mM, pH 7.4, 0.2 M NaCl), r.t., 15 minutes.

The total protein concentration of BL21 cell lysate is 0.9 mg/mL. The total protein concentration of 293F cell lysate is about 0.95 mg/mL

SDS-PAGE and western blot analysis of protein modification reactions in cell lysates

After quenching the copper reaction by addition of EDTA, the reaction mixture was mixed with 4×LDS loading buffer (Life Technologies NP0008). Protein denaturation was performed at 98°C for 5 minutes. After cooling to r.t., the sample was loaded to a 4%-20% bis-tris gel (Genscript). Electrophoretic separation was conducted at 180 V for 40 minutes. For protein staining, Coomassie soln was applied to the gel for 1h and subsequently destained with water. For Western blot analysis, the protein gel was transferred to a blot membrane using Bio-rad Tran-Blot SD Semi-Dry Electrophoretic Transfer Cell at 25V for 25 minutes. The blot membrane was subjected to a blocking process with 5% w/w aq skim milk powder in TBST buffer (150 mM NaCl, 25 mM Tris, 0.1% Tween, pH 7.4) at 4°C overnight. Anti-Biotin antibody [Hyb-8] (20 μL, ab201341) was added to the membrane in 4 mL of the blocking solution, and the membrane in the solutionwas shaken at r.t. for 1 hour. After discarding the liquid, the membrane was washed with TBS buffer (150 mM NaCl, 25 mM Tris, pH 7.4) three times. HRP-labeled Goat Anti-Mouse IgG(H+L) (20 μL, Beyotime, A0216) was added to the membrane in 4 mL of the blocking solution, and the membrane in the solutionwas shaken at r.t. for 1 hour. The membrane was washed with TBS buffer (150 mM NaCl, 25 mM Tris, pH 7.4) three times. Membrane imaging was performed on Amersam Imager 680 using 2 ml Super ECL Detection Reagent (Yesen Biotechnology).

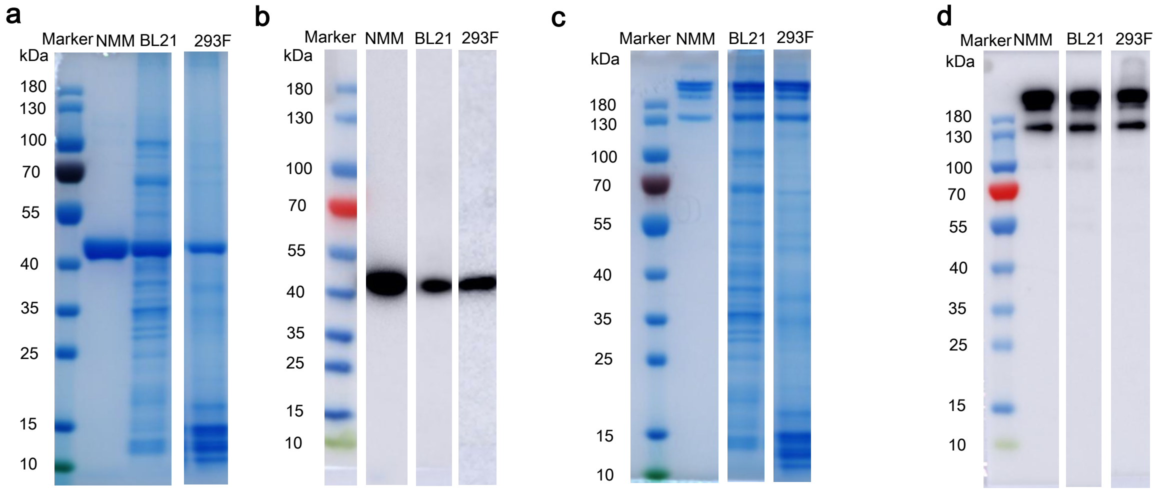

Supplementary Figure 51. Characterizations of *CAST* fusion protein labeling in BL21 or 293F cell lysate. a. SDS-PAGE conjugation reaction analysis of MBP (C-***CAST***) in NMM buffer, MBP (C-***CAST***) in BL21 cell lysate, and MBP (C-***CAST***) in 293F cell lysate. **b**. Western blot analysis of **a**. **c**. SDS-PAGE conjugation reaction analysis of **Tra-*CASTi*** in NMM buffer, **Tra-*CASTi*** in BL21 cell lysate, and **Tra-*CASTi*** in 293F cell lysate. **d**. Western blot analysis of **c.** For **a** and **b**, pure MBP (C-***CAST***) labeling in NMM buffer conditions: MBP (C-***CAST*)** (10 µM), **1C** (1 mM), $CuCl_2 \cdot 2H_2O$ (1 mM), NMM buffer (50 mM, pH 7.4, 0.2 M NaCl), 37 °C, 5 minutes. For MBP (C-***CAST***) labeling in BL21 cell lysate: MBP (C-***CAST*)** (5 µM), **1C** (0.5 mM), $CuCl_2 \cdot 2H_2O$ (0.5 mM), BL21 cell lysate in NMM buffer (50 mM, pH 7.4, 0.2 M NaCl), r.t., 5 minutes. For MBP (C-***CAST***) labeling in 293F cell lysate: MBP (C-***CAST*)** (2 µM), **1C** (1 mM), $CuCl_2 \cdot 2H_2O$ (1 mM), 293F cell lysate in NMM buffer (50 mM, pH 7.4, 0.2 M NaCl), 37 °C, 5 minutes. For **c** and **d**. protein labeling conditions: **Tra-*CASTi*** (2 µM), **1C** (1 mM), $CuCl_2 \cdot 2H_2O$ (1 mM), in NMM buffer (50 mM, pH 7.4, 0.2 M NaCl) or BL21/293F cell lysate in NMM buffer (50 mM, pH 7.4, 0.2 M NaCl), r.t., 15 minutes. The total protein concentration of BL21 cell lysate is 0.9 mg/mL. The total protein concentration of 293F cell lysate is 0.95 mg/mL. Each experiment was repeated 3 times independently with similar results. Source data are provided as a Source Data file.

15.NMR spectra

a. NMR

1. Chemicals

DMSO-d_6 was bought from Cambridge Isotope Laboratories, Inc. (MA, U.S.).

2. Sample preparation for NMR measurements

Each sample was dissolved with 500 µl DMSO-d_6 and transferred into 5 mm NMR tubes for NMR analysis.

3. NMR measurements

All NMR experiments including ^{1}H, ^{13}C, ROESY, HSQC and HMBC were performed at 25 °C on a Bruker NEO 600 MHz NMR spectrometer (600.23 MHz for proton frequency)

equipped with an QCI-F Cryoprobe or Bruker NEO 500 MHz NMR spectrometer (500.3 MHz for proton frequency) equipped with an BBO Cryoprobe. Spectra were processed using Topspin 4.1.1 software. ^1H NMR chemical shifts were referenced to residual non-deuterated DMSO (2.5 ppm). Chemical shifts for ^{13}C NMR spectra were referenced to DMSO-d_6 (39.5ppm)

4. Peak assignments of polypeptide backbone

Peak assignments of polypeptide backbone were completed using 1D and 2D experiments (^1H, ^{13}C, ROESY, HSQC, and HMBC)

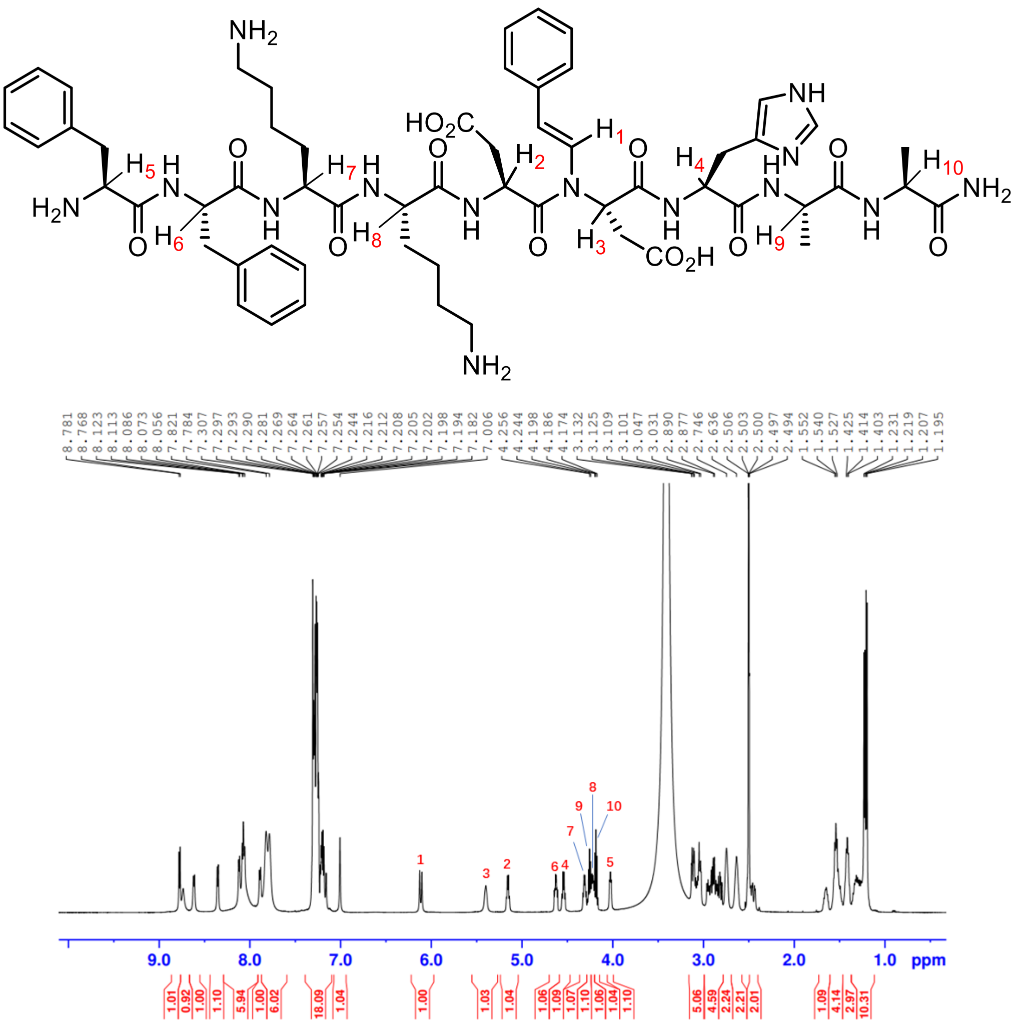

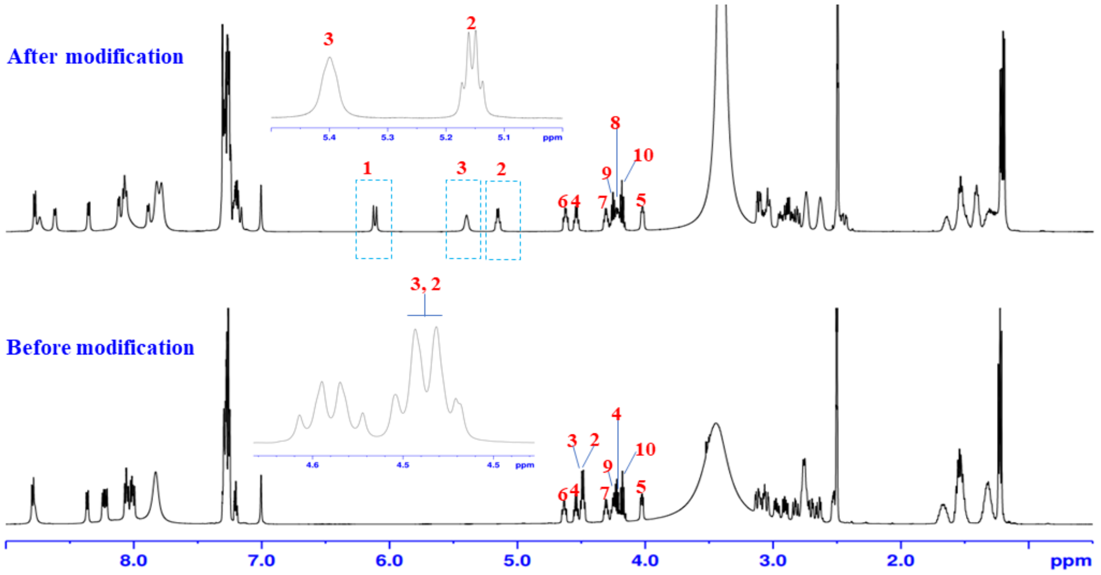

Supplementary Figure 52. ^{1}H NMR spectra (600 MHz, DMSO-d_6, 298K) of peptide before and after modification.

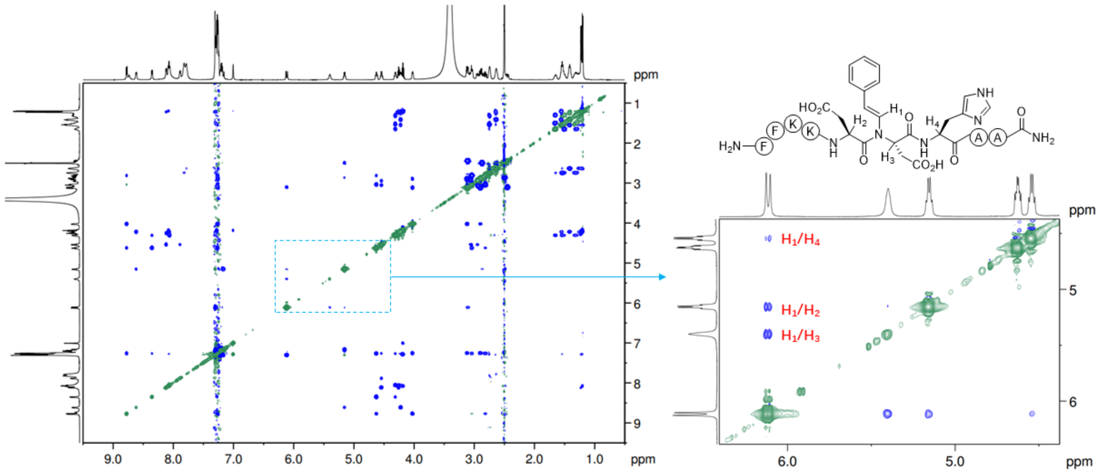

Supplementary Figure 53. ^{1}H-^{1}H ROESY spectra (600 MHz, DMSO-d_6, 298K) of peptide after modification.

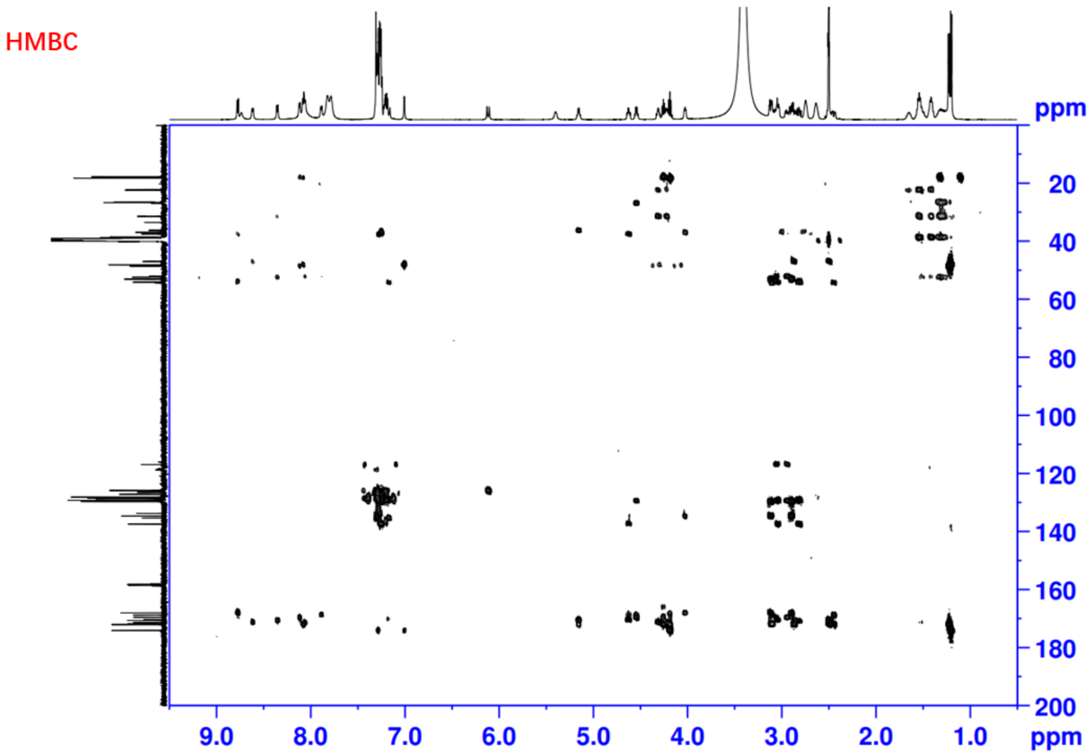

Supplementary Figure 54. HMBC spectra (600 MHz, DMSO-d_6, 298K) of peptide after modification.

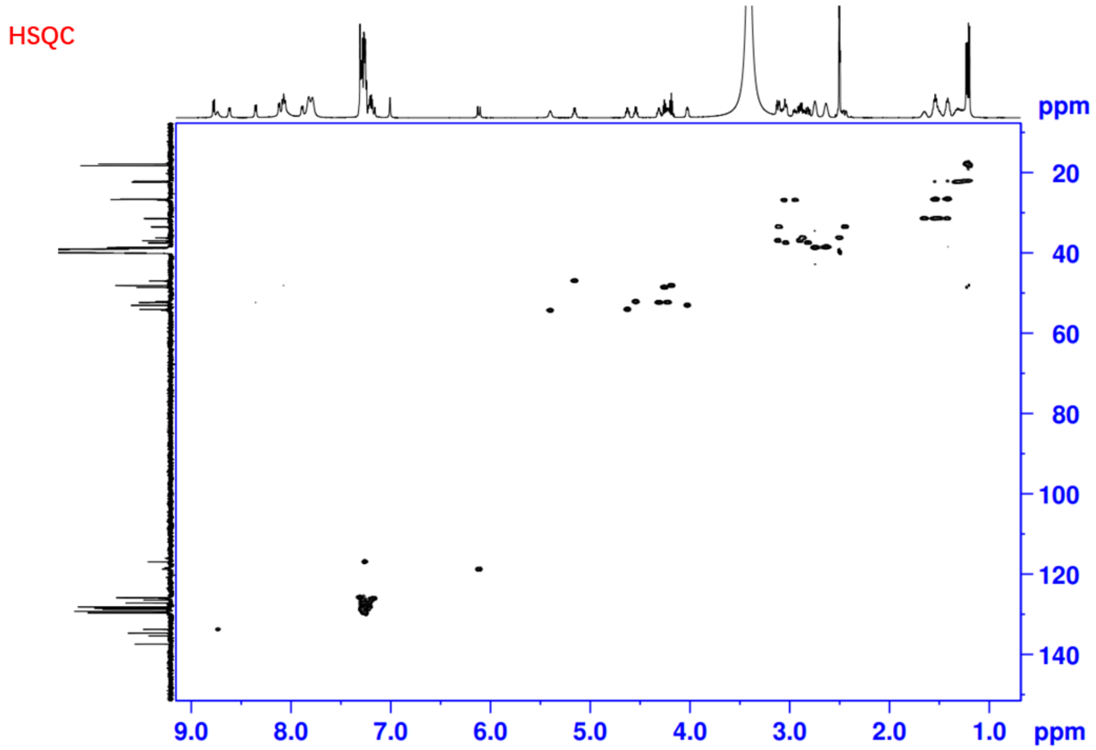

Supplementary Figure 55. HSQC spectra (600 MHz, DMSO-d_6, 298K) of peptide after modification.

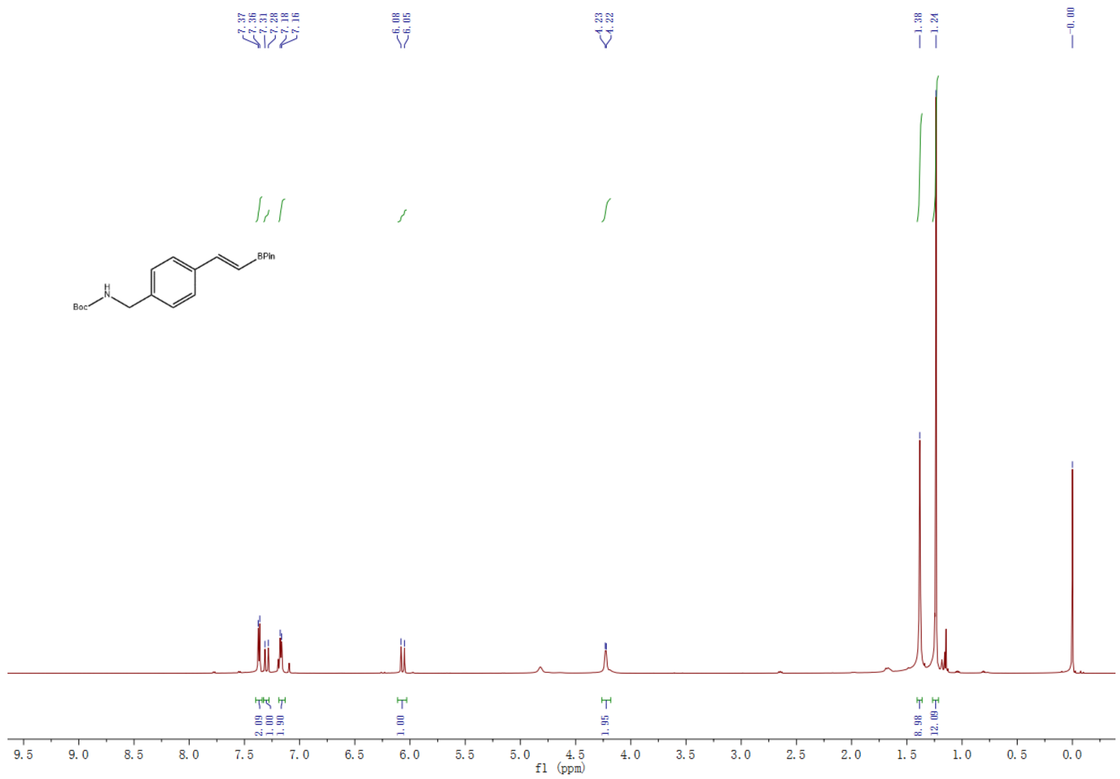

Supplementary Figure 56. ^{1}H NMR spectrum of compound **S1**

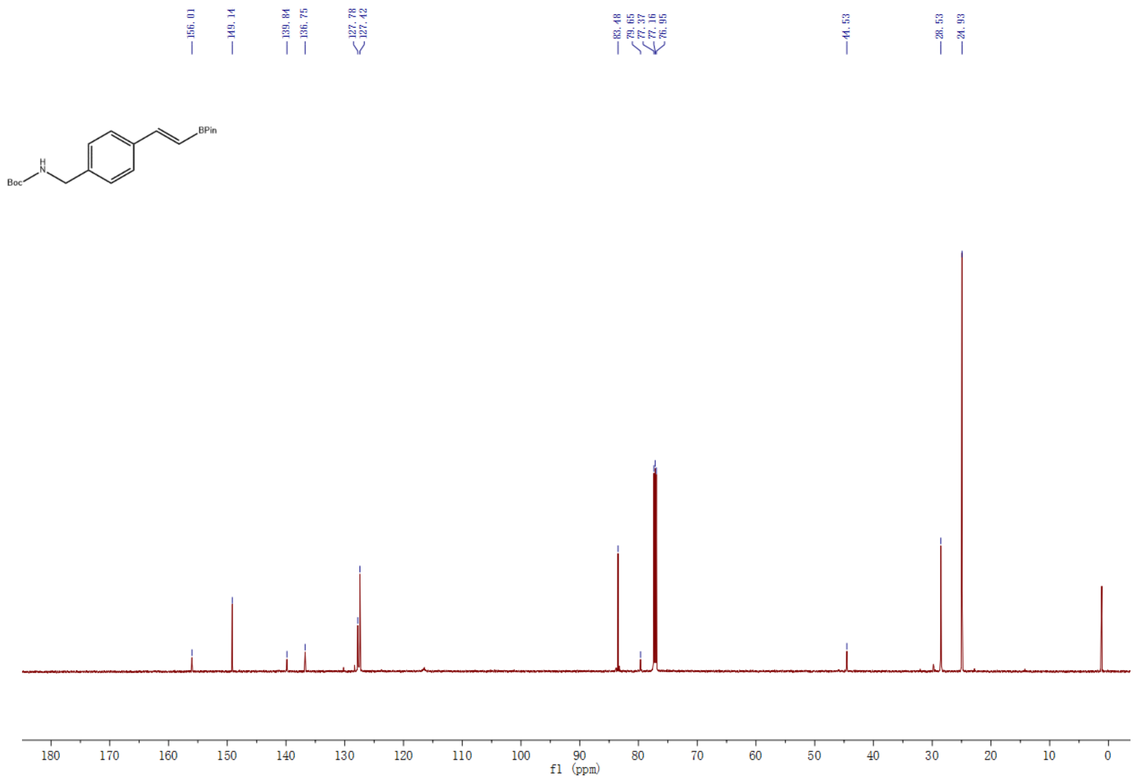

Supplementary Figure 57. ^{13}C NMR spectrum of compound **S1**

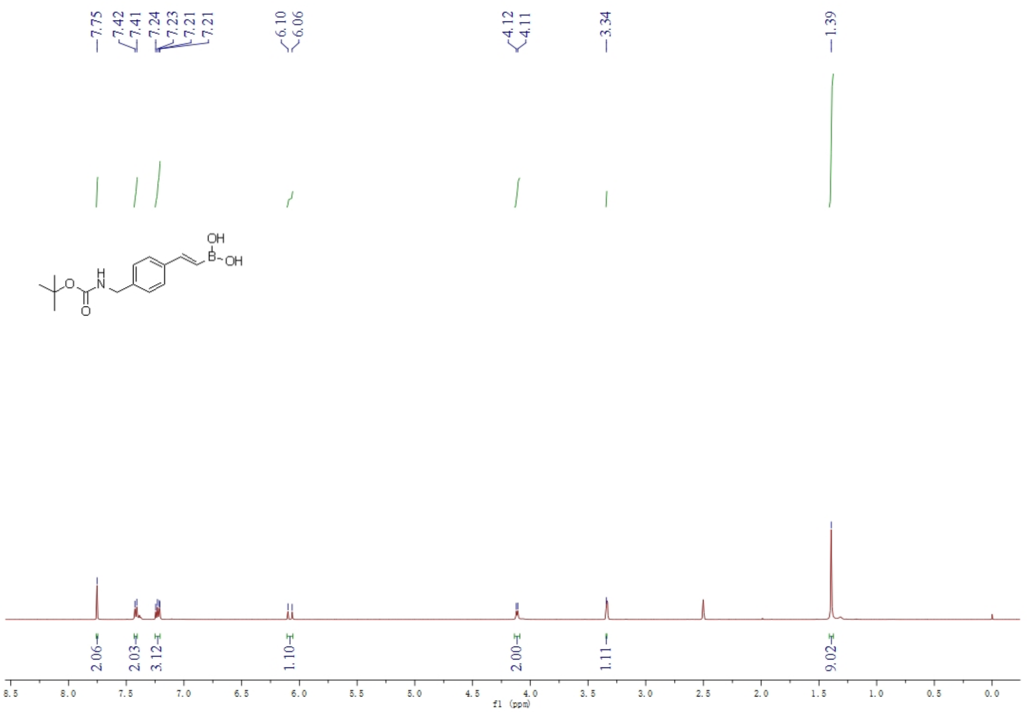

Supplementary Figure 58. ^{1}H NMR spectrum of compound **S2**

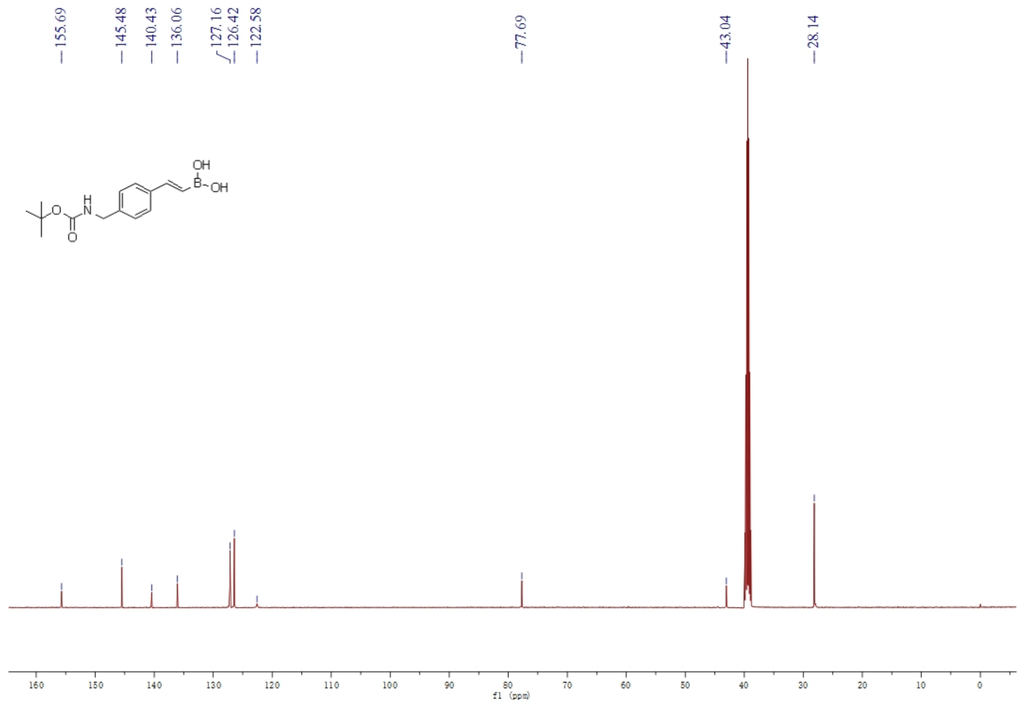

Supplementary Figure 59. ^{13}C NMR spectrum of compound **S2**

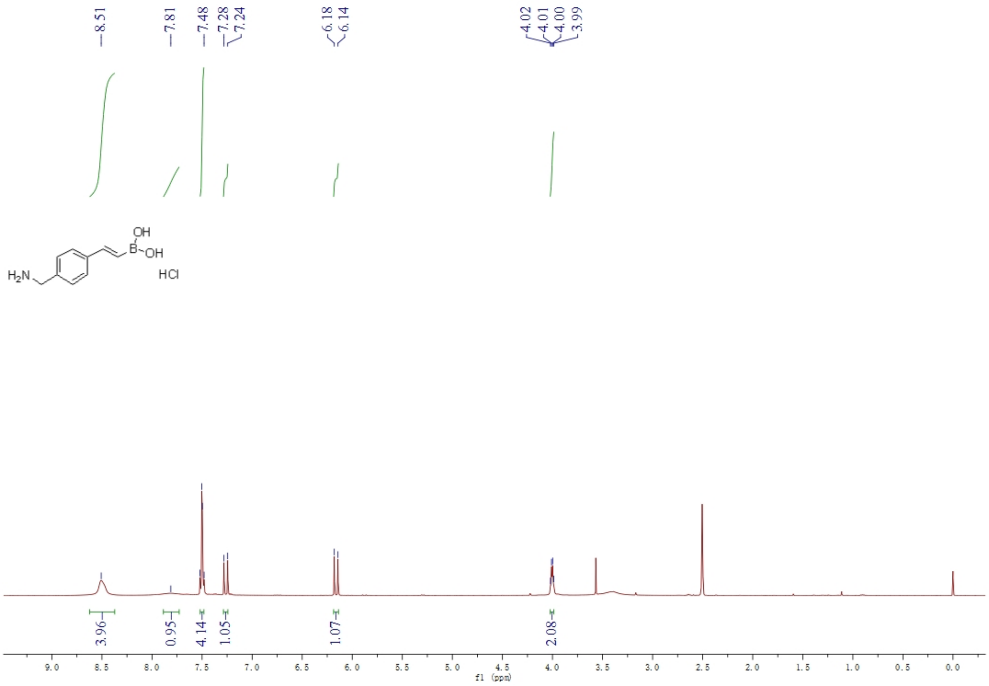

Supplementary Figure 60. ^{1}H NMR spectrum of compound **S3**

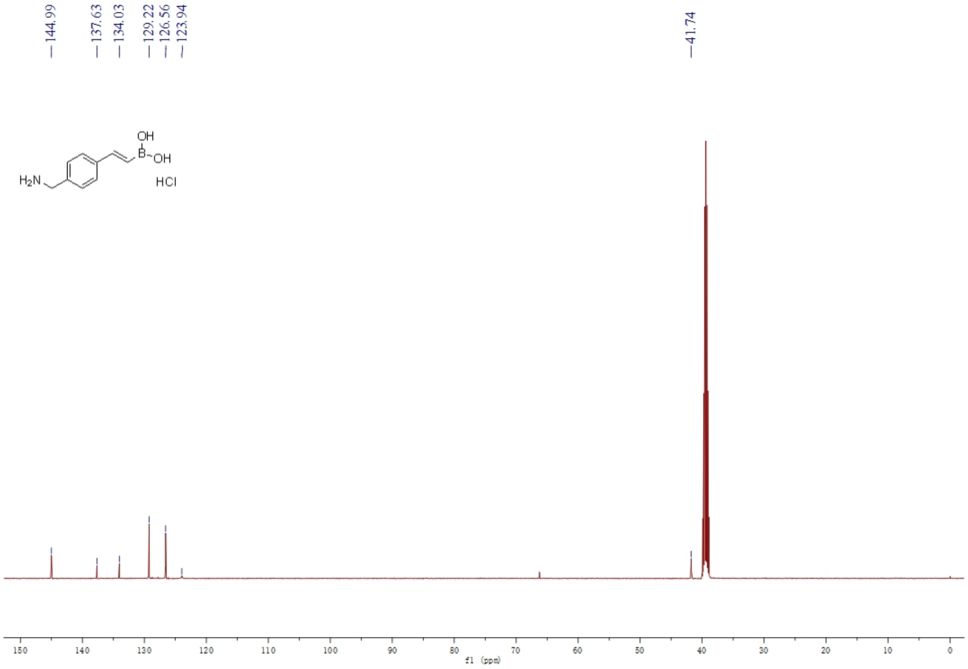

Supplementary Figure 61. ^{13}C NMR spectrum of compound **S3**

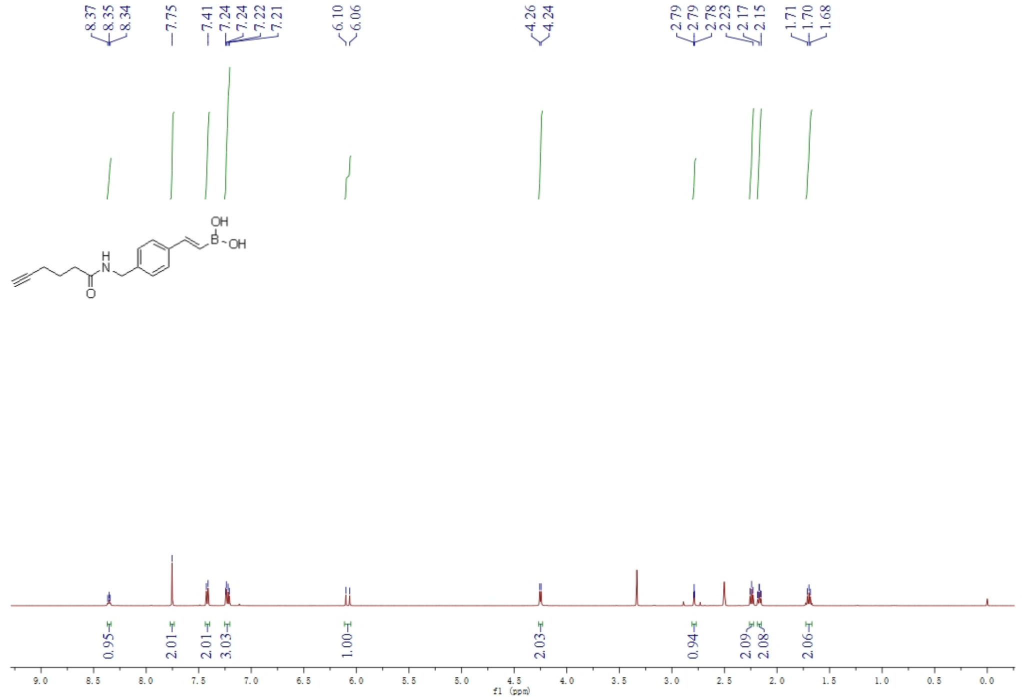

Supplementary Figure 62. ^{1}H NMR spectrum of compound **1A**

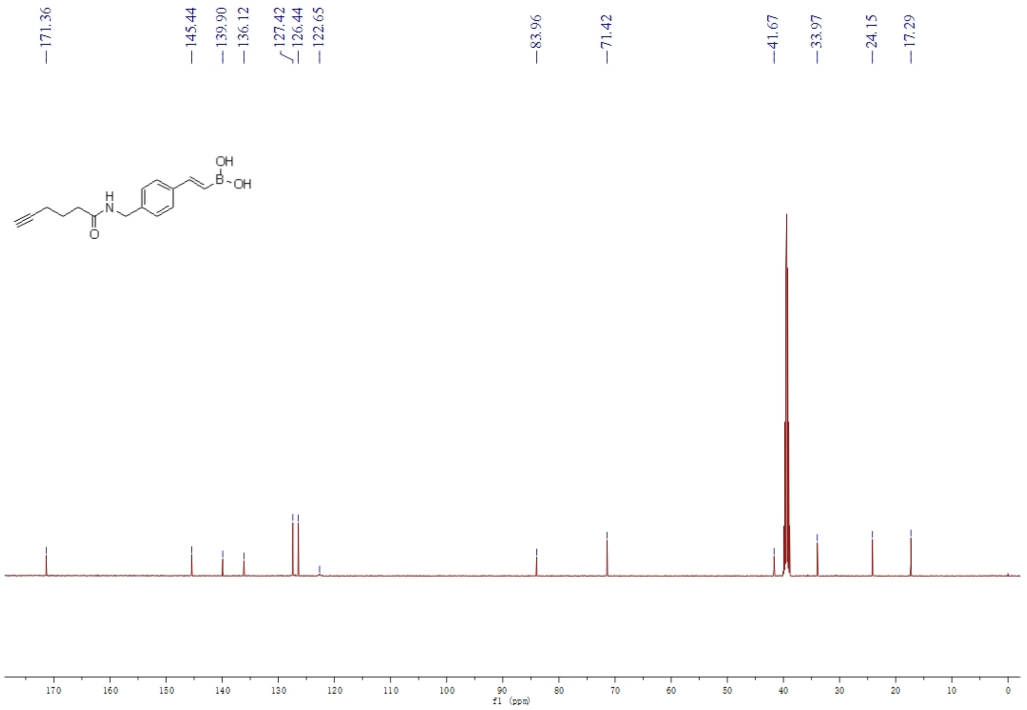

Supplementary Figure 63. ^{13}C NMR spectrum of compound **1A**

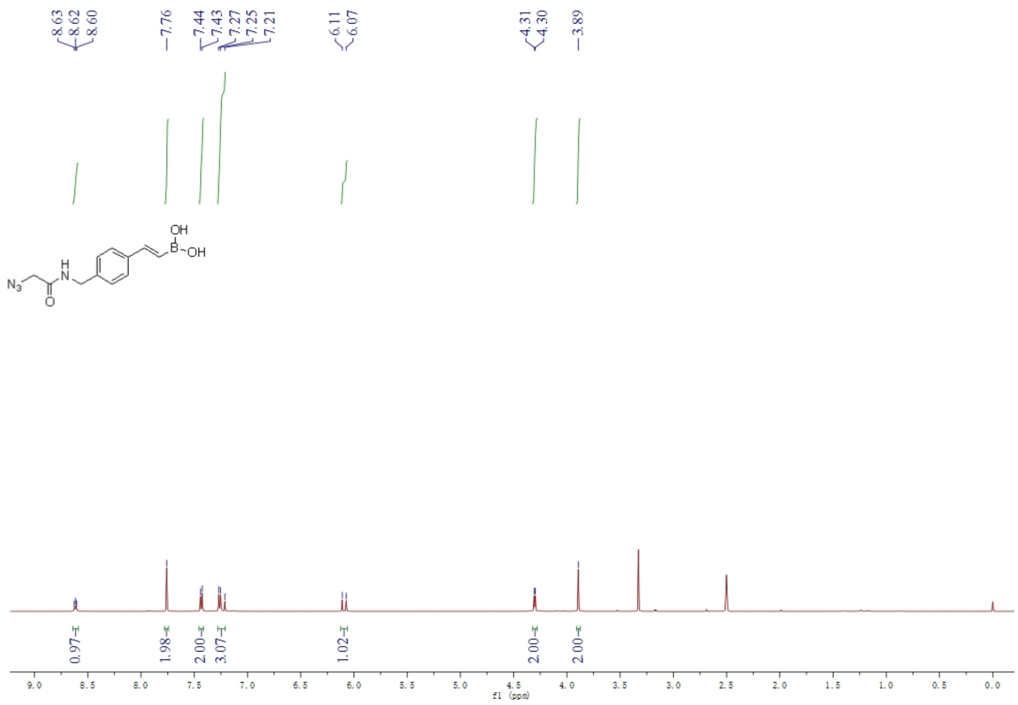

Supplementary Figure 64. ^{1}H NMR spectrum of compound **1B**

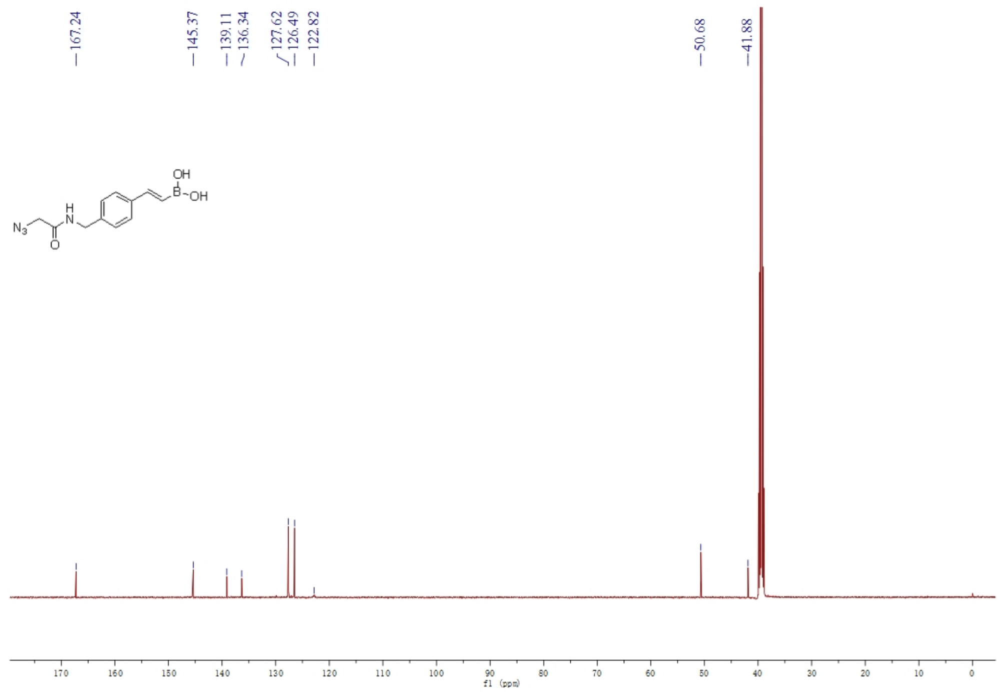

Supplementary Figure 65. ^{13}C NMR spectrum of compound **1B**

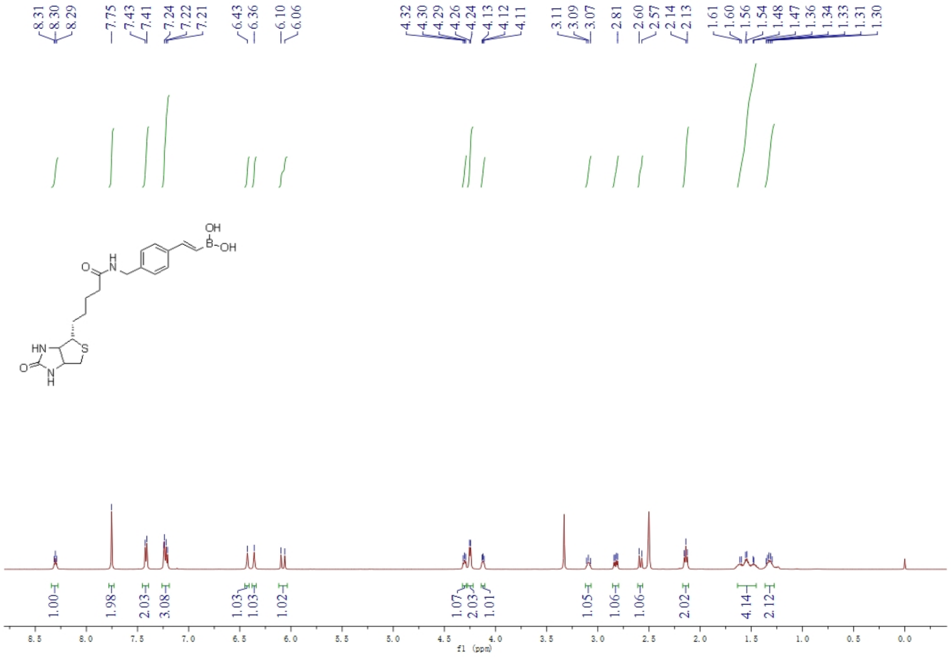

Supplementary Figure 66. ^{1}H NMR spectrum of compound **1C**

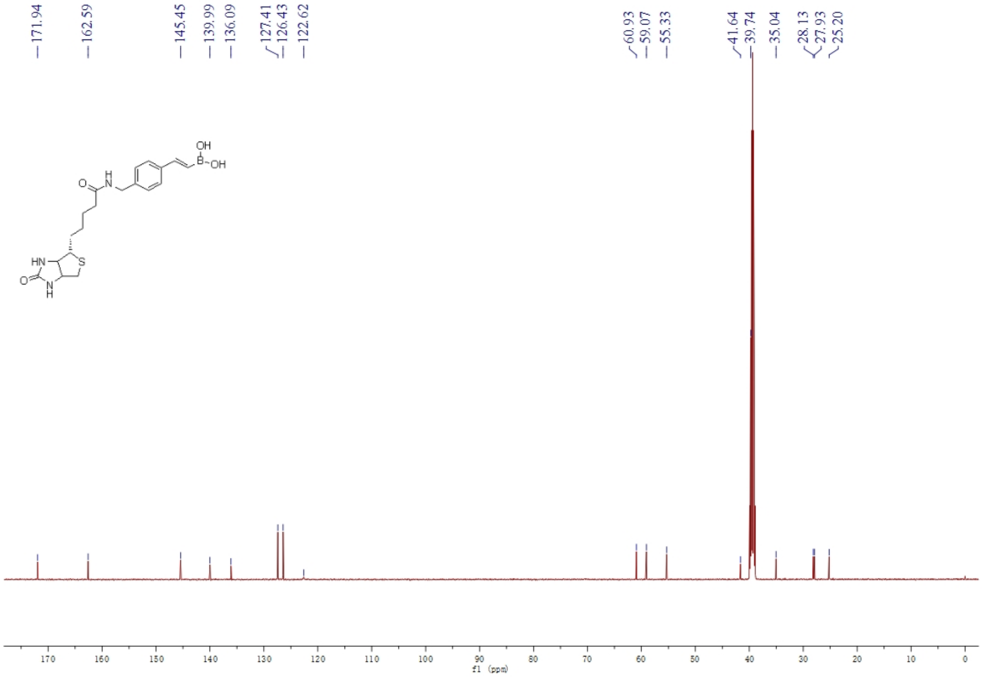

Supplementary Figure 67. ^{13}C NMR spectrum of compound **1C**

16. Supplementary References

1. Dang, B. et al. SNAC-tag for sequence-specific chemical protein cleavage. *Nat Methods* **16**, 319-322 (2019).
2. Dooley, C.T. & Houghten, R.A. in Combinatorial Peptide Library Protocols. (ed. S. Cabilly) 13-24 (Humana Press, Totowa, NJ; 1998).
3. Thomas, S. et al. Borane-Catalysed Hydroboration of Alkynes and Alkenes. *Synthesis* **50**, 803-808 (2017).
4. Tang, F. et al. One-pot N-glycosylation remodeling of IgG with non-natural sialylglycopeptides enables glycosite-specific and dual-payload antibody-drug conjugates. *Org Biomol Chem* **14**, 9501-9518 (2016).
5. Chuang, S. et al. Antibody-drug conjugates containing an anti-mesothelin antibody and uses thereof. WO2021248048.
6. Guo, L. et al. Engineered trimeric ACE2 binds viral spike protein and locks it in "Three-up" conformation to potently inhibit SARS-CoV-2 infection. *Cell Res* **31**, 98-100 (2021).
7. McMahon, C. et al. Yeast surface display platform for rapid discovery of conformationally selective nanobodies. *Nature Structural & Molecular Biology* **25**, 289-296 (2018).
8. Zhang, C. et al. Pi-Clamp-mediated cysteine conjugation. *Nat Chem* **8**, 120-128 (2016).
9. M. Bird, J. Nunes and M. Frigerio, Methods in Molecular Biology, Humana Press Inc., 2020, vol. 2078, pp. 113–129.
10. Ohata, J., Zeng, Y., Segatori, L. & Ball, Z.T. A Naturally Encoded Dipeptide Handle for Bioorthogonal Chan-Lam Coupling. *Angew Chem Int Ed.* **57**, 4015-4019 (2018).
