# Supplementary material for: Copper assisted sequence-specific chemical protein conjugation at a single backbone amide
Source: Nat Commun. 2023 Dec 5;14:8063. doi: 10.1038/s41467-023-43753-7 (PMC10698186; doi:10.1038/s41467-023-43753-7)

**Fig. 2a**

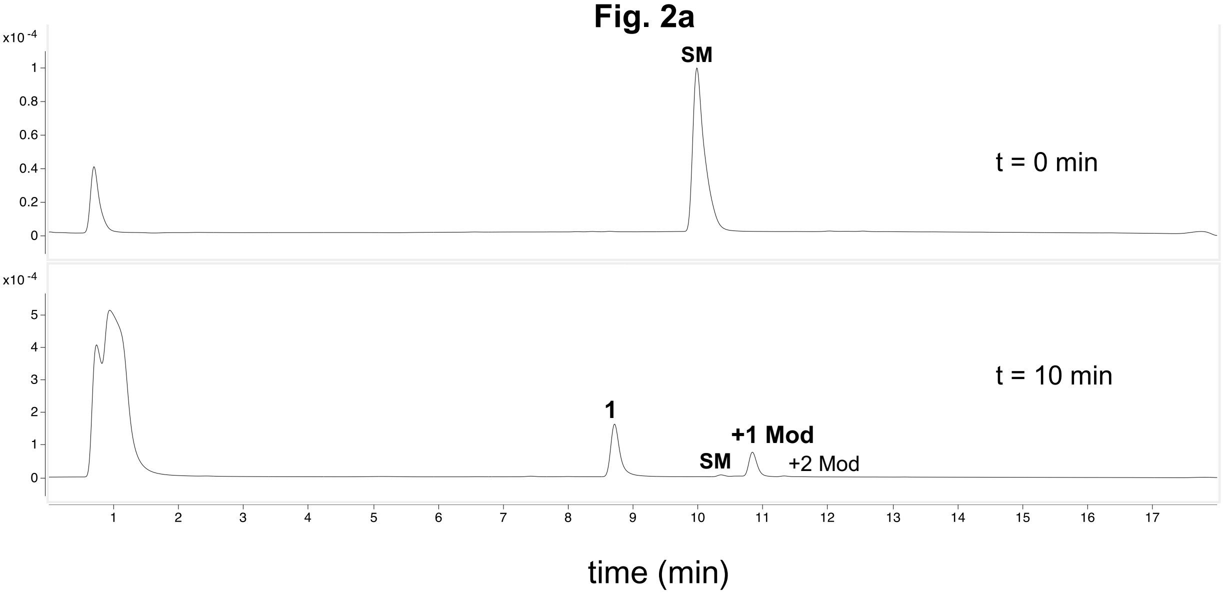


**Fig. 4b**

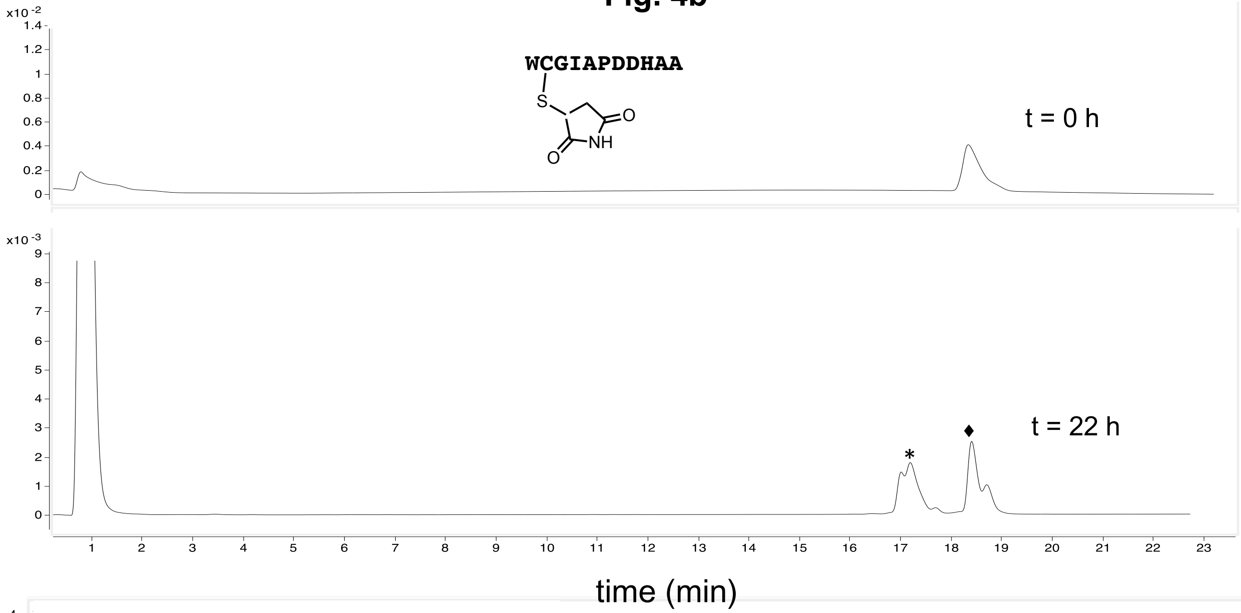


**Fig. 4b**

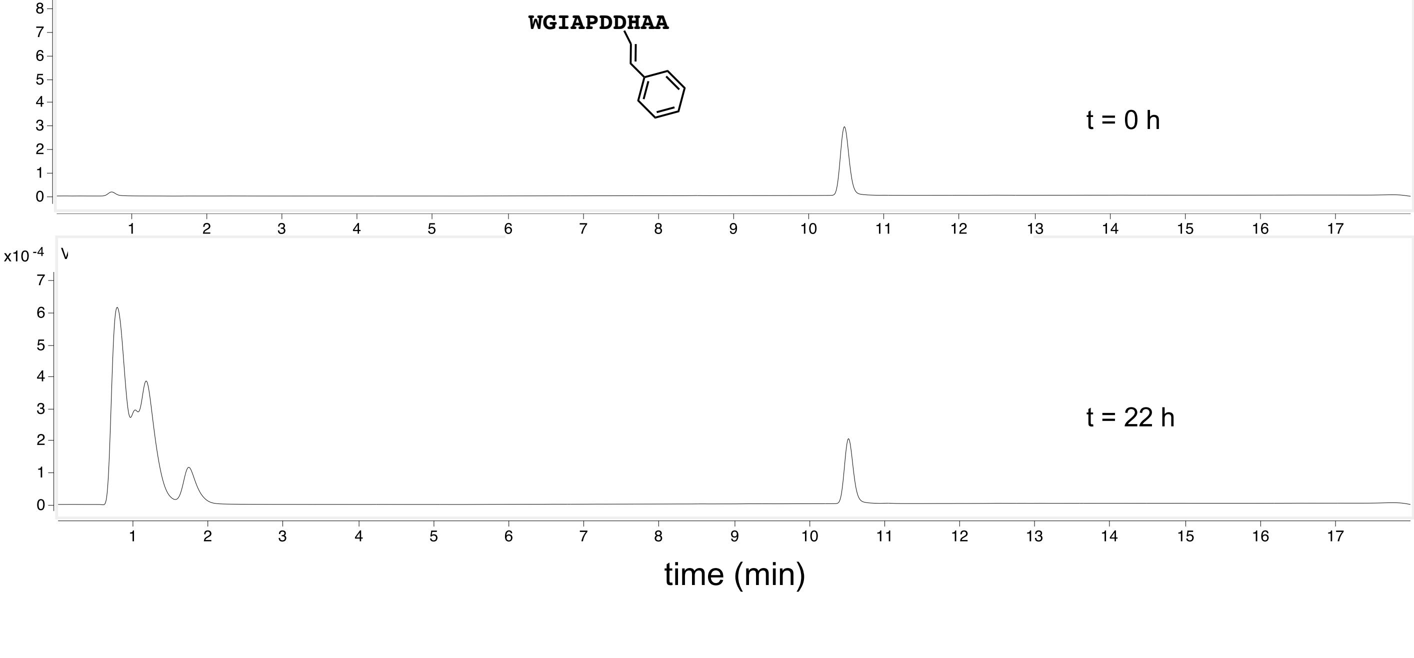

## Supplementary Figure 31

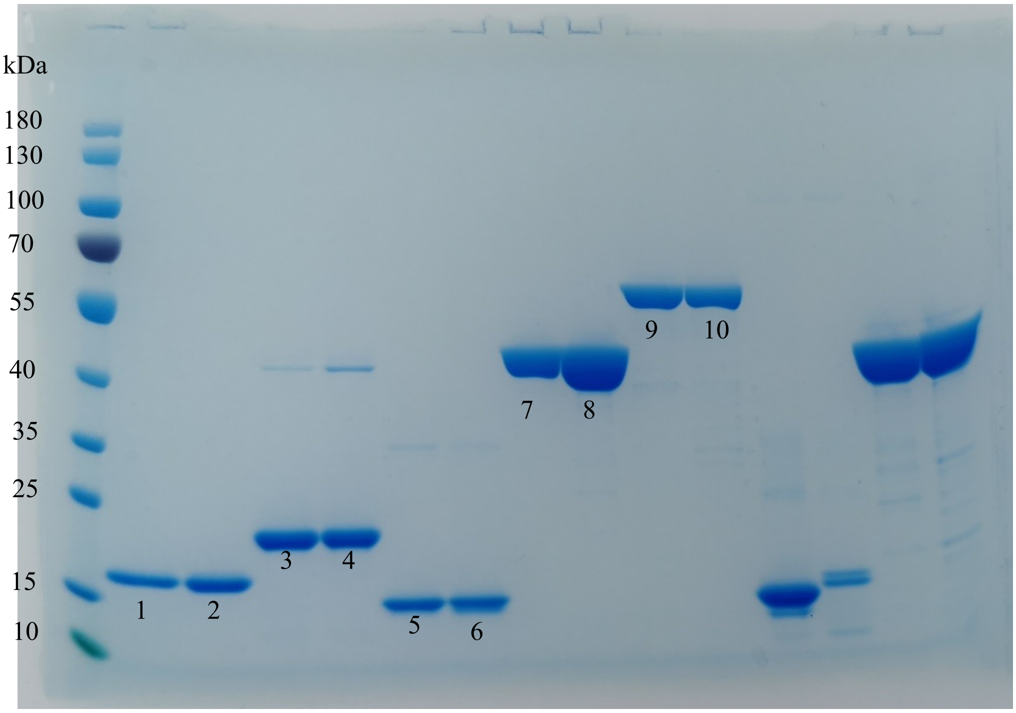


Lane1:SMT3 (C-***CAST)***
Lane2:SMT3(C-***CAST)*** after modification with **1**
Lane3:maileimide modified soratse (C-***CAST)***
Lane4:maileimide modified soratse (C-***CAST)*** after modification with **1**
Lane5:nanobody (C-***CAST)***
Lane6:nanobody (C-***CAST)*** after modification with **1**
Lane7:MBP (C-***CAST)***
Lane8:MBP (C-***CAST)*** after modification with **1**
Lane9:Trigger factor (C-***CAST)***
Lane10:Trigger factor (C-***CAST)*** after modification with **1**

**Supplementary Figure 50a.**

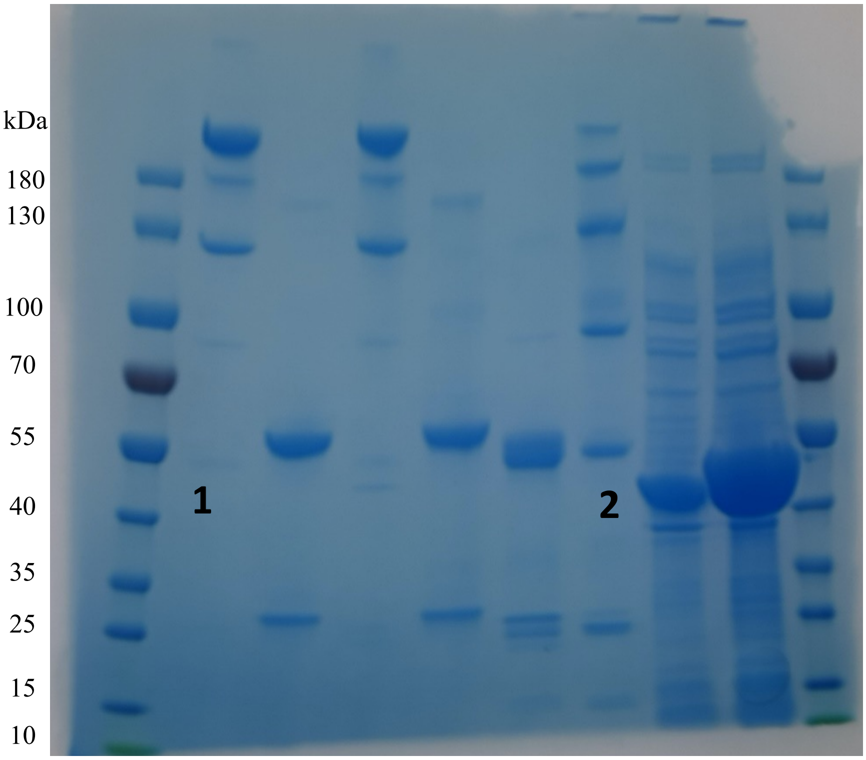


Lane1:**Tra-*CASTi*** , boiling 5min
Lane2:**Tra-*CASTi***+0.6 mM TCEP +5.6 equiv. bis-sulfone-$PEG_3$-azide

**Supplementary Figure 50b.**

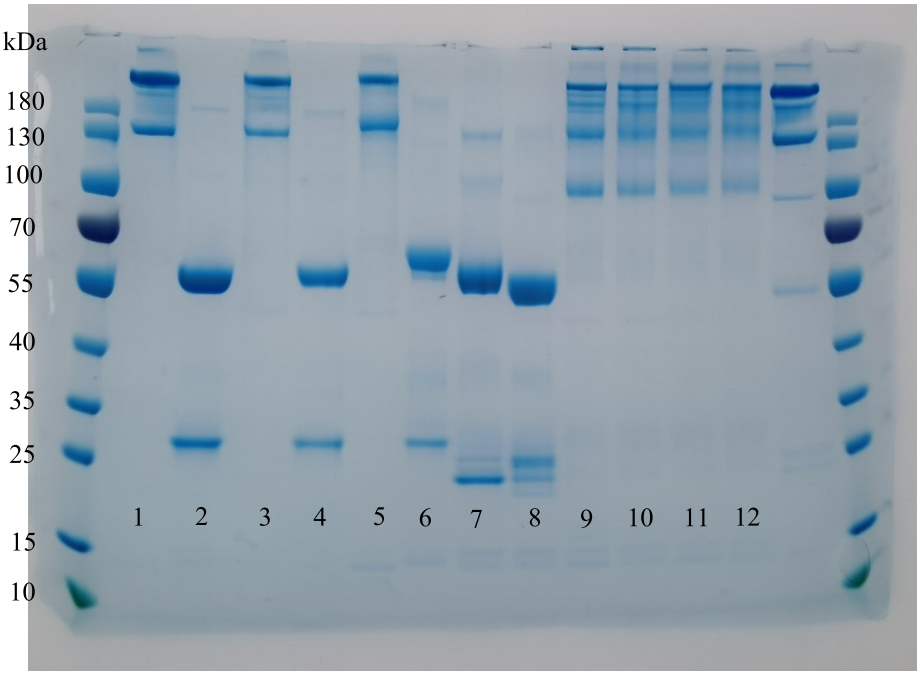


Lane1:**Tra-*CASTi***
Lane2:**Tra-*CASTi***+10 mM DTT boiling 5 min
Lane3:**Tra-*CASTi*** modified with **1B**
Lane4:**Tra-*CASTi*** modified with **1B**+10 mM DTT boiling 5 min
Lane5:**Tra-*CASTi***-MMAE
Lane6:**Tra-*CASTi***-MMAE+10 mM DTT boiling 5 min
Lane7:**Tra-*CASTi***+0.6 mM TCEP
Lane8:**Tra-*CASTi***+0.6 mM TCEP boiling 5 min
Lane9:**Tra-*CASTi***+0.6 mM TCEP +10 equiv. bis-sulfone-PEG$_3$-azide
Lane10:**Tra-*CASTi***+0.6 mM TCEP +20 equiv. bis-sulfone-PEG$_3$-azide
Lane11:**Tra-*CASTi***+0.6 mM TCEP +50 equiv. bis-sulfone-PEG$_3$-azide
Lane12:**Tra-*CASTi***+0.6 mM TCEP +100 equiv. bis-sulfone-PEG$_3$-azide

## Supplementary Figure 51

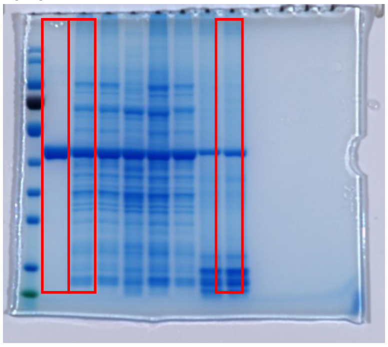


## Supplementary Figure 51

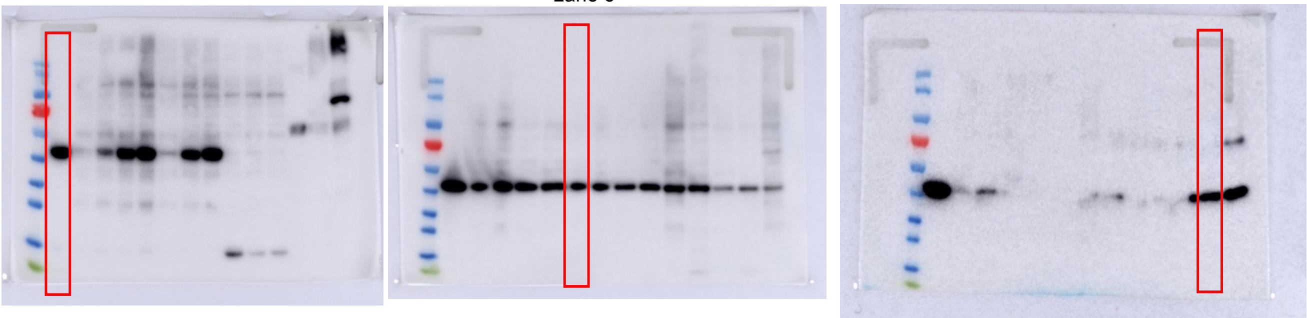


## Supplementary Figure 51

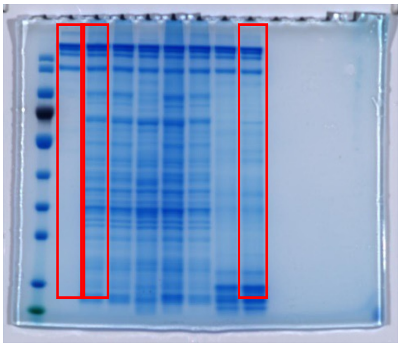


## Supplementary Figure 51

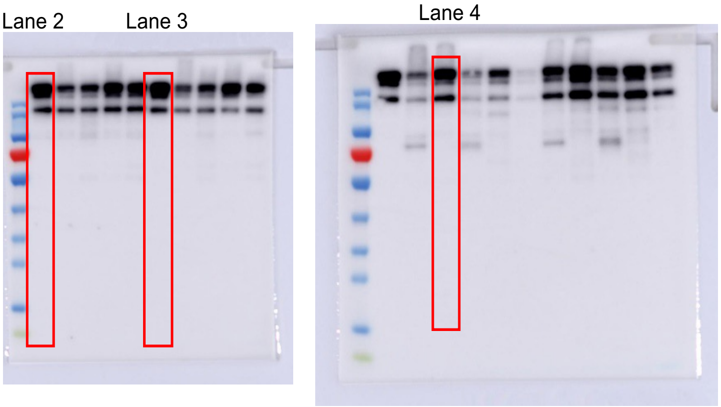

Supplement: Supplementary file 3 — Source Data [file 41467_2023_43753_MOESM3_ESM.zip › Source Data.pdf]
